# Proteomic Analyses Reveal Common Promiscuous Patterns of Cell Surface Proteins on Human Embryonic Stem Cells and Sperms

**DOI:** 10.1371/journal.pone.0019386

**Published:** 2011-05-03

**Authors:** Bin Gu, Jiarong Zhang, Ying Wu, Xinzong Zhang, Zhou Tan, Yuanji Lin, Xiao Huang, Liangbiao Chen, Kangshou Yao, Ming Zhang

**Affiliations:** 1 The Institute of Genetics, College of Life Sciences, Zhejiang University, Hangzhou, China; 2 Zhejiang Institute of Planned Parenthood Research and Zhejiang Human Sperm Bank, Hangzhou, China; 3 The Institute of Cell and Developmental Biology, College of Life Sciences, Zhejiang University, Hangzhou, China; 4 The Institute of Genetics and Developmental Biology, Chinese Academy of Sciences, Beijing, China; Rockefeller University, United States of Amercia

## Abstract

**Background:**

It has long been proposed that early embryos and reproductive organs exhibit similar gene expression profiles. However, whether this similarity is propagated to the protein level remains largely unknown. We have previously characterised the promiscuous expression pattern of cell surface proteins on mouse embryonic stem (mES) cells. As cell surface proteins also play critical functions in human embryonic stem (hES) cells and germ cells, it is important to reveal whether a promiscuous pattern of cell surface proteins also exists for these cells.

**Methods and Principal Findings:**

Surface proteins of hES cells and human mature sperms (hSperms) were purified by biotin labelling and subjected to proteomic analyses. More than 1000 transmembrane or secreted cell surface proteins were identified on the two cell types, respectively. Proteins from both cell types covered a large variety of functional categories including signal transduction, adhesion and transporting. Moreover, both cell types promiscuously expressed a wide variety of tissue specific surface proteins, and some surface proteins were heterogeneously expressed.

**Conclusions/Significance:**

Our findings indicate that the promiscuous expression of functional and tissue specific cell surface proteins may be a common pattern in embryonic stem cells and germ cells. The conservation of gene expression patterns between early embryonic cells and reproductive cells is propagated to the protein level. These results have deep implications for the cell surface signature characterisation of pluripotent stem cells and germ cells and may lead the way to a new area of study, i.e., the functional significance of promiscuous gene expression in pluripotent and germ cells.

## Introduction

At the beginning of life, terminally differentiated germ cells fuse to generate a totipotent stem cell, the fertilised egg. After a series of cleavages, the last stem cell type that can form any cell type, pluripotent stem cells, forms at the blastocyst stage [1], [2]. A small group of pluripotent stem cells, the germline stem cells, are set aside at this stage and will ultimately derive the germ cells of the next generation and sustain the life of the species[3], [4]. Therefore, the terminally differentiated germ cells and highly plastic pluripotent stem cells are two critical points in the circle of life. The relationship between these two cell types, distinct from the point of view of differentiation potential, is a basic question of life science.

It has been postulated that pluripotent stem cells have similar gene expression profiles compared to germ cells [5]. For example, many transcription factors that are critical for pluripotency maintenance like OCT4 and DPPA3 are also expressed through primordial germ cells to mature gametes [6]. A distinctive characteristic of gene expression profiles is that the promiscuous expression of functional and tissue specific genes is not supposed to exist in pluripotent and reproductive cells [7], [8]. However, this characteristic has largely been demonstrated at the mRNA level [5], [7], [9], [10]. As pluripotent stem cells and germline stem cells have loose chromatin structures and/or express transcription factors that promote promiscuous gene expression, such as Aire, promiscuous gene expression may be leaky expression and never lead to the translation of functional proteins [11], [12], [13], [14], [15], [16]. Determining whether pluripotent stem cells and germ cells have similar promiscuous expression at the protein level is important for the establishment of a functional relationship between pluripotent stem cells and germ cells.

Cell surface proteins exercise critical functions in both pluripotent stem cells and germ cells [17], [18]. Our previous study showed that mES cells, pluripotent stem cells derived from mouse blastocyst inner cells mass, promiscuously express a large variety of functional and tissue specific cell surface proteins through proteomic methods [19]. We also demonstrated that hES cells, pluripotent stem cells derived from human blastocyst inner cell masses, express some tissue specific surface proteins [19]. Whether the cell surface proteome of hES cells have a similar promiscuous characteristic compared to mES cells and whether this similarity extends to human germ cells are important questions.

In this study, we used an earlier described biotin-labelling coupled streptavidin affinity purification method and purified cell surface proteins from hES cells and normal mature human sperm. More than 1000 surface proteins were identified from both cell types by LC-MS/MS analysis. A bioinformatic analysis showed that hES and hSperm both promiscuously expressed diverse functional and tissue specific cell surface proteins. Comparative analyses indicated that mES, hES and hSperm cells show a similar surface proteomic pattern. Our results indicate that promiscuous gene expression might be a conserved property of pluripotent stem cells and germ cells and its functional significance deserve further study.

## Results

### Proteomic analyses of cell surface proteins on hES cells and hSperm

**Figure 1:**
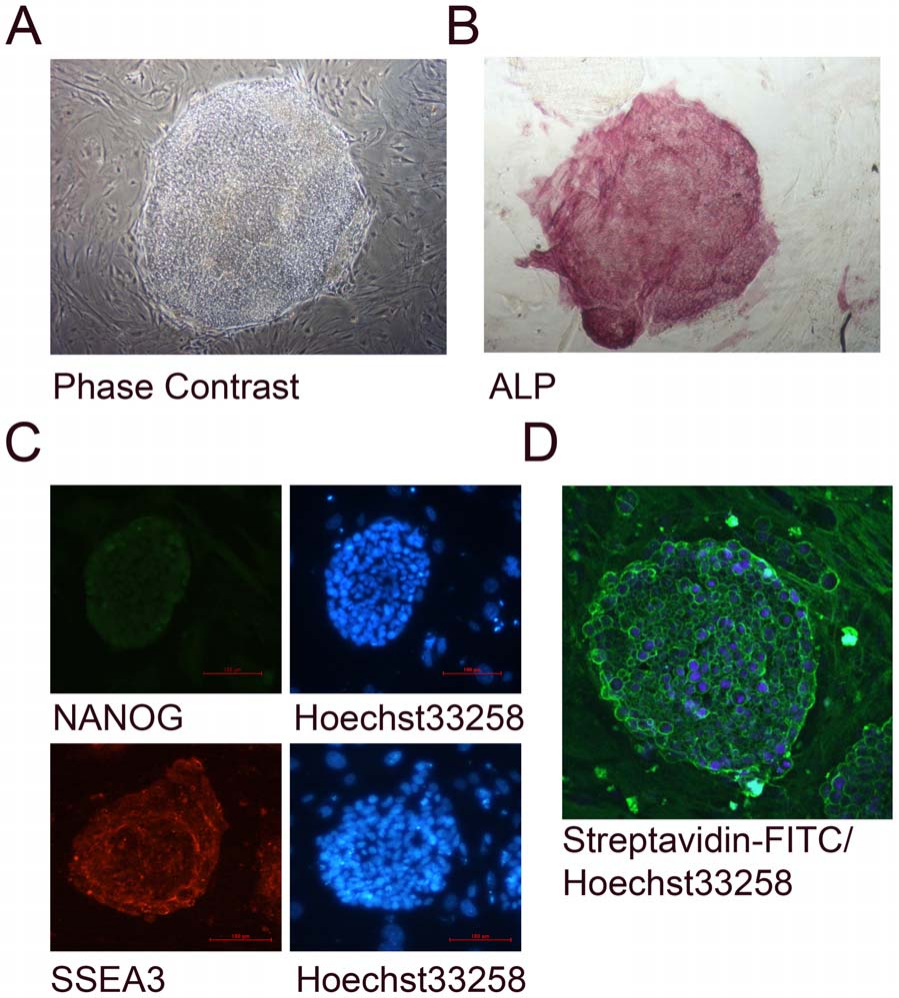
Labelling of hES cell surface proteins. A. hES cells exhibit a typical flattened colony morphology. B. hES cells express alkaline phosphatase (ALP). C. ICC staining shows that hES cells express the pluripotency markers NANOG and SSEA-3. D. Biotin labelling of hES cell surface proteins. Streptavidin-FITC staining shows that the biotin was labelled on the cell surface proteins.

**Figure 2:**
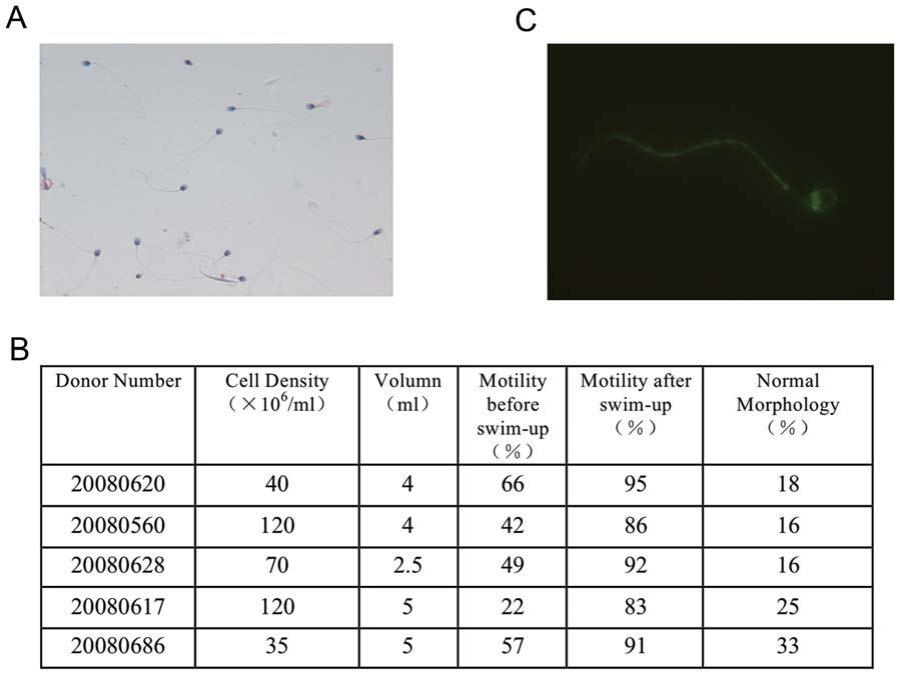
Labelling of hSperm cell surface proteins. A. Sperm showed a normal morphology after a modified Papanicolaou stain. B. Sperm quantifications used in this study. As shown here, swim-up efficiently enriched motile sperm to more than 85%. C. Biotin labelling of sperm cell surface proteins. Streptavidin-FITC staining showed that most of the biotin was labelled on the cell surface protein.

To explore the expression patterns of hES and hSperm surface proteins, we purified cell surface proteins from these cell types by biotin labelling and identified the proteins by LC-MS/MS. Before labelling, the quality of hES cells and hSperm was evaluated. As shown in Fig. 1A, B and C, the hES cells used in this study grew with a typical flattened colony morphology and homogeneously expressed alkaline phosphatase (ALP), NANOG and SSEA3 [20]. Moreover, we mechanically isolated hES cells with an undifferentiated morphology for proteomic study. Therefore, most hES cells used in this study were undifferentiated. hES cell surface proteins were labelled with membrane-impermeable biotin reagents. Labelling efficiency was monitored by streptavidin-FITC staining. As shown in Fig. 1D, most cells were labelled with biotin on the cell surface, although some intracellular labelling was observed, which can be explained by the staining of apoptotic cells that is common in hES populations. As shown in Fig. 2A, the sperm cells displayed a normal morphology, and the swim-up technique efficiently enriched our sample for motile sperm cells (Fig. 2B). The surface proteins of the hSperm were then labelled with membrane-impermeable biotin reagents, and the labelling efficiency was monitored by streptavidin-FITC staining. As shown in Fig. 2C, the majority of the biotin signal was located on the cell surface.

**Figure 3:**
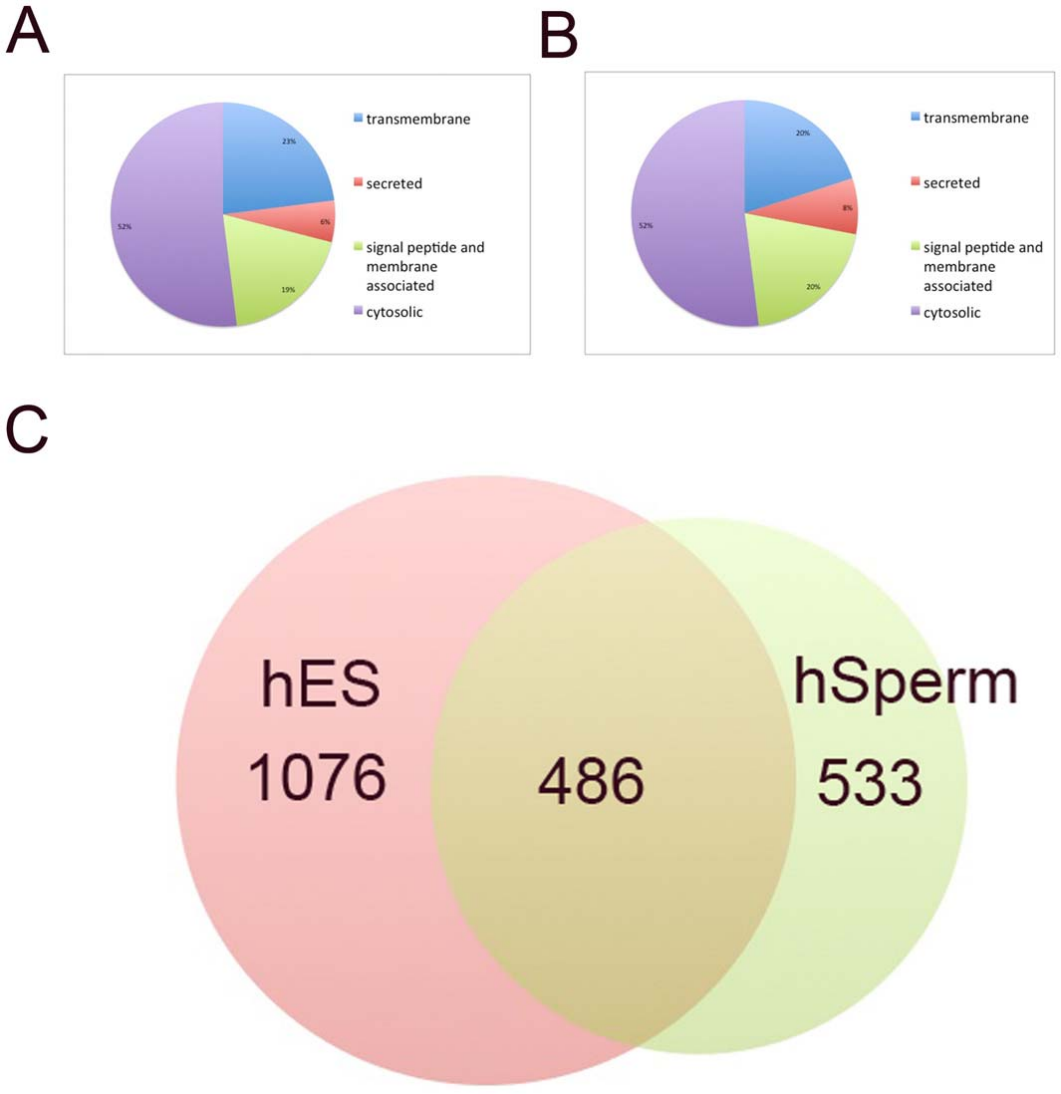
Proteomic identification of hES and hSperm proteins. A. Subcellular distribution of hES proteins. B. Subcellular distribution of hSperm proteins. C. Cross comparison of hES and hSperm cell surface proteins.

The biotin-labelled proteins were resolved by SDS-PAGE and analysed by LC-MS/MS. On hES cells, 5405 proteins were identified, and 3468 proteins were identified on hSperm. The transmembrane structure and signal peptides were predicted using SOSUI software [21]. Proteins annotated as ‘membrane’ in UniProt Database or those predicted to contain transmembrane domains or signal peptides were annotated as general membrane proteins. As shown in Figs. 3A and 3B, about 50% of the proteins identified on both cell types are general membrane proteins, which is consistent with other reports that used these same methods[22]. Transmembrane proteins and secreted proteins were annotated as cell surface proteins for further analysis. To this end, 1560 and 1019 cell surface proteins were identified on hES and hSperm cells, respectively (Tables S1, S2). We first evaluated the expression of 400 randomly selected surface proteins by RT-PCR on hES cells, and 328 of them were confirmed to be expressed. Therefore, our results should be at least 82% accurate when considering hES cells. As we performed protein purification and identification under the same experimental conditions to characterise hSperm, the accuracy should be similar. A direct comparison of protein identifiers yielded 487 identical surface proteins between the two cell types (Fig. 3C). It indicated that from the point of view of the exact protein identity about half of the hSperm surface proteins were identical to the hES cells.

**Figure 4:**
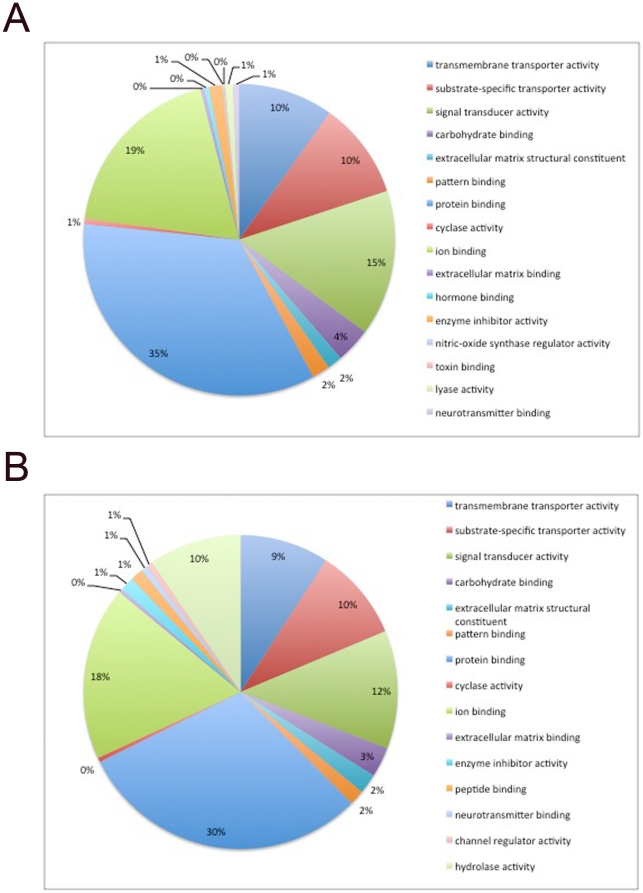
Functional categorisation of hES and hSperm cell surface proteins. A. Functional categorisation of hES cell surface proteins. B. Functional categorisation of hSperm cell surface proteins.

Thereafter, we performed gene ontology analyses according to the Molecular Function annotations using DAVID software [23], [24]. As shown in Figs. 4A and 4B, the cell surface proteins of hES and hSperm cells performed wide varieties of molecular functions, and each functional category included many functional surface proteins. The three functional categories that included the largest fraction of cell surface proteins in hES and hSperm were both ‘transmembrane transporter activity’, ‘signal transduction activity’ and ‘ion binding’, and the general distribution of cell surface protein functions was similar. These data indicate that the cell surface proteins of hES cells and hSperm possess a common functional pattern.

### ES cells and sperm both express diverse signal molecules

Signal ligands and receptors play critical roles in the self-renewal and differentiation of ES cells, and they also play critical roles in sperm function [25], [26], [27]. Consistently, surface proteins annotated to be ‘signal transducers’ are significantly enriched in both hES cells and hSperm (By Molecule Function enrichment study by DAVID, Data not shown). As we have previously shown that mES cells express signal receptors and ligands from 48 different signalling pathways, we surveyed hES and hSperm cell data for signalling receptors and ligands from these pathways and compared the results with mES[19]. As shown in Table 1, except for the AXL signal pathway and the vomeronasal receptors, receptors and ligands from all these signal pathways were present on the cell surfaces of hES cells and hSperm. As no obvious ortholog of the vomeronasal organ is present in humans, it is reasonable that no vomeronasal receptors are present on human cells. Among these signal pathways, some including the Wnt, FGF, TGF/Activin, Notch, natriuretic peptide and EGF pathways have been characterised as functional in hES cells and sperm [25], [26], [27], [28], [29], [30]. However, functions of most other signal pathways like olfactory receptor pathways, semaphorin pathways, the Slit signal pathway and the TRP channel pathway on hES and hSperm cells remain to be characterised. These data indicate that mES cells, hES cells and hSperm cells also possess much more versatile signal transforming abilities than ever thought.

**Table 1:** Comparison of signal pathways on mES, hES and hSperm cells.

signal pathway	mES	hES	hSperm
Acetylcholine	+	+	+
angiopoietin	+	+	+
AXL	+	-	-
BMP	+	+	+
cannabinoid	+	+	+
chemokine	+	+	+
cholecystokinin	+	+	+
Cytokine	+	+	+
EGF	+	+	+
Eph	+	+	+
FGF	+	+	+
Flt	+	+	+
GABA	+	+	+
GDF	+	+	+
Glutamate	+	+	+
Glycine	+	+	+
Orphan GPCR	+	+	+
growth hormone	+	+	+
hedgehog	+	+	+
HGF	+	+	+
hormone	+	+	+
IGF	+	+	+
Insulin	+	+	+
interferon	+	+	+
interleukin	+	+	+
LIF	+	+	+
LPA	+	+	+
natriuretic peptide	+	+	+
netrin	+	+	+
neuropeptide	+	+	+
Neurotrophic factor	+	+	+
Nogo	+	+	+
Notch	+	+	+
olfactory	+	+	+
PCP	+	+	+
progestin	+	+	+
prolactin	+	+	+
prostaglandin	+	+	+
PTPR	+	+	+
relaxin	+	+	+
semaphorin	+	+	+
Sphingosine	+	+	+
Slit	+	+	+
Taste	+	+	+
TGF/Activin	+	+	+
TNF	+	+	+
Toll like receptor	+	+	+
TRP Channels	+	+	+
vomeronasal	+	+	+
Wnt	+	+	+

**Figure 5:**
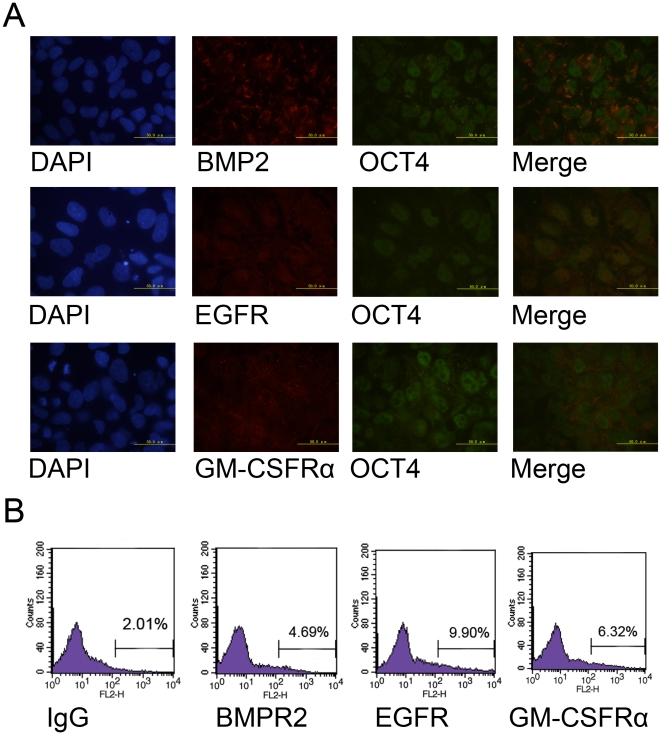
Signal molecules on hES and hSperm cells. A. Immunocytochemistry staining showed that hES cells expressed BMP2, EGFR and GM-CSFRα. First panel from the left, DAPI staining. Second panel, ICC staining of cell surface proteins on hES cells. Third panel, ICC staining of OCT4 on hES cells. Fourth panel, merge of surface proteins and OCT4 staining, bars indicate 50 µm. B. Flow cytometry analysis showed that hSperms heterogeneously expressed BMPR2, EGFR and GM-CSFRα.

Besides proteomic characterisations, we also examined the expression of some signalling molecules *in situ* by immunocytochemistry (ICC) and flow cytometry. As hES cells are vulnerable during single cell separation, we reasoned that flow cytometry analysis might introduce some artefacts considering the expression pattern of signal molecules[31]. In order to show that undifferentiated hES cells in the highly compacted colonies expressed the signalling molecules, we costained the signalling molecules with OCT4 and examined the staining samples under high magnificent microscope(1000X). As shown in Fig. 5A, hES cells expressed BMP2, EGFR and GM-CSFRa at the protein level. Co-staining of the signal molecules with the pluripotent marker OCT4 demonstrated that the signalling molecules were expressed on undifferentiated hES cells. ICC staining also showed that the staining strength of the signalling molecules varied among OCT4 positive cells, which indicates that hES cells heterogeneously express cell surface signalling molecules. Moreover, it is also shown that the signalling molecules were not homogeneously expressed on the cell surface of hES cells, but formed foci like structures, which might indicate the existence of subcellular functional complexes. For hSperm cells, we examined the expression of signalling molecules by flow cytometry. As shown in Fig. 5B, hSperm expressed EGFR, GM-CSFRa and c-Kit receptors. However, only a subset of hSperm strongly expressed these receptors. These results indicate that hSperm heterogeneously express these cell surface signalling molecules. As we have previously described that mES cells globally express signal molecules, the global expression of signal molecules might be a common characteristic of ES cells and sperms.

### hES cells and hSperm express diverse tissue specific cell surface proteins

**Figure 6:**
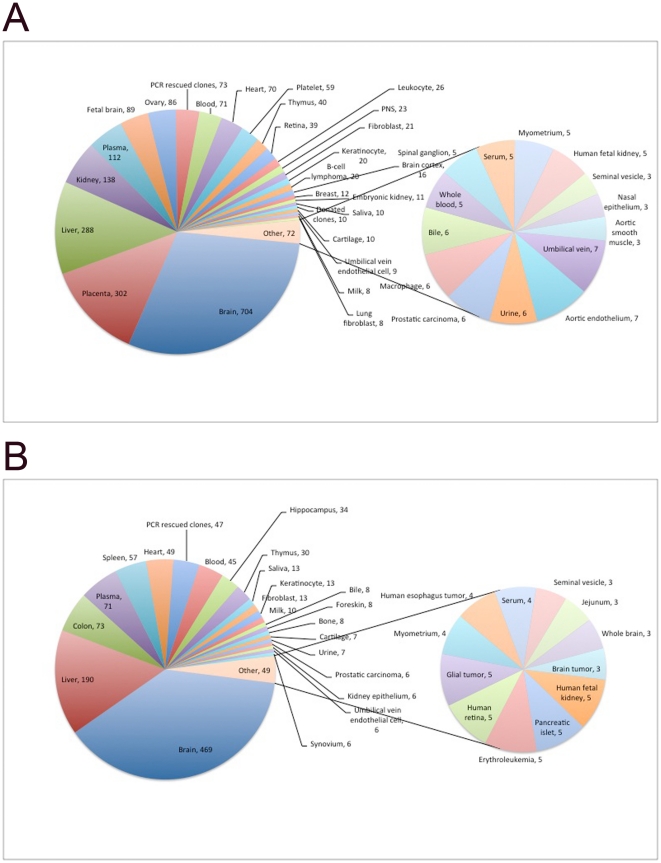
Tissue specificity of hES and hSperm cell surface proteins. A. hES cells expressed tissue specific cell surface proteins of a wide variety of tissue types. B. hSperm cells expressed tissue specific cell surface proteins of a wide variety of tissue types.

It has been reported that hES cells promiscuously express tissue specific genes at the mRNA level [9]. It has also been shown that hES cells and mouse spermatogonial cells express the core regulator of promiscuous expression of tissue specific genes in medullary thymic epithelial cells, the Aire gene [8], [15]. Therefore, it is interesting to examine whether hES cells and hSperm promiscuously express tissue specific cell surface proteins. To this end, we analysed the tissue specificity of cell surface proteins from hES and hSperm cells according to UniProt tissue specificity annotations using DAVID software. To our surprise, of the 1560 hES cell surface proteins, 1441 were annotated as tissue specific. Of the 1019 hSperm cell surface proteins, 958 were annotated as tissue specific. As shown in Figs. 6A and B, both hES cells and hSperm express a large variety of tissue specific cell surface proteins. Brain specific surface proteins predominated the cell surface proteins of both hES and hSperm cells, which may indicate a common gene expression pattern between immunoprivileged entities like the brain and early embryo. Both hES and hSperm cells also express a large variety of liver specific genes. As many of these proteins are involved in *de novo* synthesis processes, this may indicate some extension of the self-sustenance of ES cells and germ cells. A significant difference between hES cells and hSperms is that hES cells express diverse placenta-specific cell surface proteins while hSperms do not. This might be a consequence of hES cells having the potential to derive extraembryonic tissue including placenta while hSperms do not. Besides these predominate tissues, both hES and hSperm cells also expressed tissue specific proteins of several other tissues and distribution is fairly even. We also compared data from hES and hSperm cells to data from previously obtained mouse ES cell surface proteins. The results indicate that brain and liver specific cell surface proteins predominated all three cell types and that all three cell types expressed tissue specific cell surface proteins from many tissues. These results further indicate an interspecies conservation of the expression of tissue specific cell surface proteins in embryonic stem cells and germ cells.

**Figure 7:**
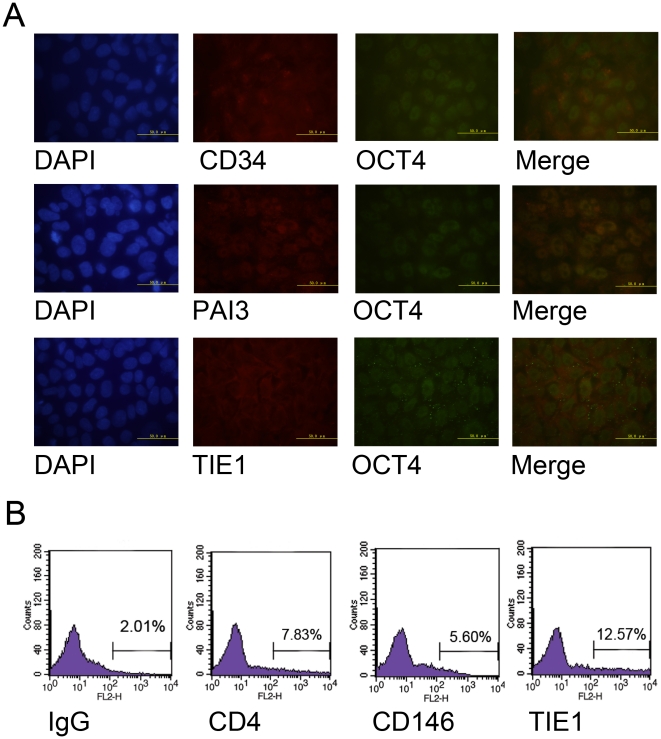
Tissue specific cell surface proteins expressed on hES and hSperm. A. Immunocytochemistry staining showed that hES cells expressed CD34, PAI3 and TIE1. First panel from the left, DAPI staining. Second panel, ICC staining of cell surface proteins on hES cells. Third panel, ICC staining of OCT4 on hES cells. Fourth panel, merge of surface proteins and OCT4 staining, bars indicate 50 µm B. Flow cytometry analysis showed that hSperms heterogeneously expressed CD4, CD146 and TIE1.

Besides proteomic analyses, we also examined the expression of tissue specific cell surface proteins in hES cells and sperm *in situ* by ICC and FC. As shown in Fig. 7A, hES cells express hematopoietic tissue specific surface protein CD34, liver specific surface protein PAI3 and endothelium specific surface protein TIE1. Co-staining with the pluripotent marker OCT4 demonstrated that tissue specific cell surface proteins were expressed on undifferentiated hES cells. The results also showed that the staining strength of tissue specific surface proteins among OCT4 positive hES cells varied, which indicates that the hES cells heterogeneously express tissue specific cell surface proteins like mES cells. Then, we analysed the expression of tissue specific cell surface proteins on hSperm by flow cytometry. As shown in Fig. 7B, hSperm heterogeneously express T-cell specific surface protein CD4, melanocyte specific surface protein CD146 and endothelium specific protein TIE1. These results indicate that the global expression of tissue specific cell surface proteins might be a common characteristic of ES cells and sperms.

## Discussion

### A common pattern of promiscuous expression of cell surface proteins on ES cells and germ cells

It is known that pluripotent stem cells from different species employ a similar core transcriptional circuit that consists of Oct4, Sox2 and Nanog to sustain pluripotent identity [17], [32], [33]. It is also known that germline cells from different developmental stages express some pluripotent specific transcription factors including OCT4 and DPPA3[6], [34], [35], [36], [37]. It has recently been proposed that pluripotent embryonic and pluripotent germline stem cells possess an open chromatin structure, and many functional and tissue specific genes in the genome are poised for expression [14]. Since we previously demonstrated that mES cells promiscuously express a large variety of functional and tissue specific cell surface proteins at the protein level [19], it is interesting to ask whether this promiscuous pattern is conserved between pluripotent stem cells and germ cells from different species. Some previous studies using whole cell proteomics have indicated that mouse multipotent germline stem cells have similar proteomic patterns compared to pluripotent stem cells [38], [39]. However, whether this similarity also exists in humans, whether it is propagated to differentiated gametes and whether it exists for cell surface proteins are important questions. Here, we demonstrate that like mES cells, hES cells and hSperm promiscuously express functional and tissue specific cell surface proteins in a heterogeneous manner. These results indicate that the similarity of the transcription regulating network and the epigenetic characteristics between pluripotent stem cells and germ cells are translated to a similar surface protein pattern.

### Complex signal network controls the behaviour of pluripotent stem cells and germ cells

Some signal pathways have been demonstrated to play critical functions in pluripotent stem cells and germ cells [26], [40], [41], [42]. However, our results indicate that both pluripotent stem cells and germ cells express a large variety of signal receptors and ligands of different signal pathways heterogeneously at the protein level. Many of these have never been reported to function in these cells types. These results indicate that the behaviour of pluripotent stem cells and germ cells might be regulated by much more complex signalling networks than previously thought, and the interaction between different subpopulations of pluripotent stem cells and germ cells might be important. The heterogeneous expression of cell surface proteins on hSperm cells might especially contribute to the competition of sperm for fertilisation.

### Implications into the differentiation potency determination of stem cells

What determines the differentiation potency of different stem cell types is a basic question in the biological science [43]. Previously, scientists preferred a model that defined transcription circuits consisting of a small number of stem cell type specific transcription factors that determined and maintained differentiation potency [17], [32], [43]. However, recent studies have indicated that some stem cell types express genes thought to be specific to their putative differentiation descendants. Two examples are embryonic stem cells and hematopoietic stem cells. It has been shown that both human and mouse pluripotent stem cells promiscuously express many tissue specific genes at low levels [7], [9], [10]. It has also been shown that many genes specific to differentiated hematopoietic lineages are expressed in hematopoietic stem cells [44]. Therefore, it is hypothesised that the extent of gene expression plasticity may contribute to the differentiation potency determination and maintenance of stem cells [14], [44]. Our results that both embryonic stem cells and sperm promiscuously express functional and tissue specific cell surface proteins add several important lines of evidence to this hypothesis. First, as sperm are generally transcriptionally inert, it is reasonable to infer that sperm may inherit their promiscuous expression of cell surface proteins from their progeny with a plastic differentiation potential[45]. Therefore it's rational to imply that besides pluripotent embryonic stem cells, pluripotent germline stem cells may also promiscuously express cell surface proteins. This indicates that promiscuous expression may be a characteristic not restricted to pluripotent embryonic stem cells but also present in other pluripotent stem cells like germline stem cells. Second, as cell surface proteins are the major mediator of extracellular stimuli that affect cells, the versatile expression of cell surface proteins may endow stem cells the ability to differentiate in response to diverse stimuli during developmental or regeneration processes.

### Implications to the cell surface signature of pluripotent stem cells and germ cells

Cell surface markers and signatures are important for the identity characterisation of pluripotent stem cells and germ cells [46]. There have been many efforts to identify specific markers for pluripotent stem cells and germ cells. For example, the SSEA antigens, Tra antigens and some other cell surface proteins like Podocalyxin-like have been thought to be specific markers for pluripotent stem cells and germline cells [47], [48], [49]. However, most of these markers have been demonstrated to not be strictly specific for pluripotent stem cells and germ cells [50], [51], [52]. Our results indicate that a conserved promiscuous cell surface protein signature, rather than the expression of any specific markers, may mark the identity of pluripotent stem cells and germ cells. Therefore, a global view may be more important to identify pluripotent stem cells and germ cells than some specific markers.

## Materials and Methods

### Ethnical Statements

All the semen specimen donors signed a written Informed Consent Form approved by the Ministry of Health (P.R. China) for the donation of semen for scientific research use. The experiments involving semen donors and semen samples in this article have been conducted according to the principles expressed in the Declaration of Helsinki and have been approved by the review board of the Zhejiang Institute of Planned Parenthood Research & Zhejiang Human Sperm Bank (Hangzhou, China).

### Cell lines and cell culture

Gamma irradiation inactivated mouse embryonic fibroblast (MEF) feeder cells isolated from the embryos of ICR mice at gestational day 13.5 were purchased from Invitrogen (Carlsbad, CA). MEFs were thawed in DMEM supplemented with 10% foetal bovine serum (Invitrogen) at 37°C and plated at a density of 4×10^4^ cells/cm^2^ for ES culture.

Human embryonic stem cells HUES3 were provided by Harvard University (Cambridge, MA) and cultured on gamma irradiation inactivated MEFs in Knockout DMEM supplemented with 20% KOSR (Invitrogen) and 1000 ng/ml bFGF (Millipore) at 37°C in a 5% CO_2_ atmosphere [46]. The pluripotency of the hES cells was routinely analysed using ALP staining (Sigma), SSEA-3 staining and teratoma formation. In addition, the karyotype was routinely checked.

### Semen sample collection and processing

Semen specimens were obtained from five donors 22–32 years old with normal sperm quality. Sperm from each sample were stained by modified Papanicolaou stain and evaluated manually for normal morphology. After liquefaction, semen samples were subjected to swim-up as previously described[53]. Briefly, semen samples were mixed with Quinn's 1023 culture medium at a ratio of 1∶3 and then centrifuged at 200 *g* for 10 min. Then, the supernatant was discarded and 0.75 ml Quinn's 1023 culture medium supplemented with 10% human serum was gently added. The samples were then incubated for 30 min in a 5% CO_2_ incubator at 37°C to allow motile sperm to swim-up. The supernatants were collected and pooled together for proteomic analysis. Sperm motility was analysed using a Hamilton CASA IVOS Integrated Visual Optical System.

Cell surface protein labelling and affinity purification:

For biotin labelling, hES cells cultured on 50 10 cm tissue culture dishes pre-seeded with MEF feeders were incubated with 1 mg/ml Sulfo-NHS-SS-Biotin (Pierce, USA) in PBS for 30 min. Excess biotin was quenched using 10 mM glycine. Colonies showing undifferentiated morphologies were then mechanically separated from the culture under a phase contrast microscope. Next, the separated colonies were lysed by homogenisation in ice cold lysis buffer (50 mM Tris-HCl (pH 7.4), 1% NP-40 substitute (Sigma), 150 mM NaCl, 1 mM EDTA, 1 mM PMSF) using a dounce homogeniser. The homogenate was placed on ice for 1 h with gentle vortexing to extract membrane proteins. Then, the homogenate was centrifuged at 12,000 *g* to remove nuclei, unbroken cells and cell fragments. The supernatant was mixed with streptavidin-coupled LATEX (300 nm diameter) beads and vortexed at 4°C for 1 h. Contaminant proteins were excluded by harsh washing as previously described [22], and purified proteins were eluted with 100 mM DTT. About 200 µg of membrane proteins could be purified from a preparation. Labelling efficiency was monitored using FITC-streptavidin staining.

For biotin labelling of hSperm, 5×10^7^ motile sperm were incubated with 1 mg/ml Sulfo-NHS-SS-Biotin (Pierce, USA) in PBS for 30 min. Then the cell surface proteins were purified as hES cells. About 50 µg of membrane protein could be purified from 5×10^7^ cells. Labelling efficiency was monitored using FITC-streptavidin staining.

### SDS-PAGE

Purified proteins were separated by 12.5% SDS-PAGE. Following electrophoresis, gels were stained with Coomassie Blue. Gels were then dissected and subjected to LC-MS/MS analysis.

### Enzyme digestion, LC-MS/MS analysis and database searching

Enzyme digestion was performed as previously described [54]. Peptides from each band were separated on a Paradigm MS4N Nano/Capillary HS MDLC (Michrom Bioresources, Inc., USA) using a 100 µm ×150 mm C-18 reversed phase column. LC separation was conducted on a linear gradient of 5–35% buffer B for 50 min, followed by 35–90% buffer B for 10 min and 90% buffer B for 10 min (buffer A: 0.1% formic acid in a 2% acetonitrile solution, buffer B: 0.1% formic acid in a 98% acetonitrile solution) at a flow rate of 500 nl/min. Separated peptides were then analysed on an LTQ-MS (Thermol, USA) coupled to a Michrome Advanced nanospray apparatus (Microm). Peak list files were generated using Bioworks software (Applied Biosystems) using the default parameters. They were searched against databases for protein identification using the Sequest software. Search parameters were: for bi or tri valent ions, Xcorr ≥ 2; for monovalent ion, Xcorr ≥ 1.5; Deltacn ≥ 0.1. Two non-redundant peptides were identified in each unique protein.

### Antibodies

The following antibodies were used: Oct-4 (R&D, Minneapolis, USA), SSEA-3 (R&D), Nanog (Abcam, Cambridge, UK), BMP2 (HUABIO, Hangzhou, China), BMPR2 (HUABIO), CD34 (HUABIO), CD146(Huabio), c-KIT (HUABIO), EGFR (HUABIO), GM-CSFRα (HUABIO), CD4 (HUABIO), TIE-1 (HUABIO), PAI-3 (HUABIO), CD9(Huabio), R-PE-conjugated goat anti-rabbit IgG (Proteintech Chicago, USA), Alexa 488-conjugated goat anti-rat IgG and Alexa 555-conjugated goat anti-rabbit IgG (Invitrogen).

### Immunocytochemistry

For double staining, hES cells cultured on coverslips pre-seeded with feeder cells were fixed using 4% paraformaldehyde according to a standard protocol, blocked with blocking/permeating buffer (PBS with 10% goat serum and 0.3% Triton X-100) and then incubated with rat anti-human OCT4 monoclonal antibody overnight at 4°C. After washing, cells were incubated with an Alexa 488-conjugated goat anti-rat antibody for 1 h at 37°C. After washing, cells were incubated with rabbit polyclonal antibodies against cell surface molecules for 1 h at 37°C. After washing, cells were incubated with an Alexa 555-conjugated goat anti-rabbit antibody for 1 h at room temperature and then observed under a Fluorescent Microscope (Olympus, Japan). For single staining, cells were fixed using 4% paraformaldehyde according to a standard protocol, blocked with blocking/permeating buffer (PBS with 10% goat serum and 0.3% Triton X-100) and then incubated with primary antibodies for 1 h at 37°C. After washing, cells were incubated with Alexa 488-conjugated secondary antibodies for 1 h at 37°C and then observed under a fluorescent microscope (Olympus).

Biotin-labelled hES cells and hSperms were fixed with 4% paraformaldehyde overnight at 4°C and then stained with FITC-conjugated streptavidin (Sigma) for 30 min to monitor surface labelling.

### Flow cytometry

Human sperms were washed with PBS containing 3% FBS. Cells were then incubated with a primary antibody for 1 h on ice. After thorough washing, cells were incubated with fluorescent secondary antibodies for 30 min on ice. Cells were then washed with PBS and analysed by flow cytometry (BDLSR).

### RT-PCR

RT-PCR was performed as previously described [15]. Total RNA was extracted using the Trizol Reagent (Takara, Japan), retro-transcribed and then PCR-amplified. Primers were designed using the PRIMER PREMIER 5 software.

### ALP staining

ALP staining was performed with an ALP assay kit (Sigma).

### Bioinformatic analyses

The subcellular localisations of the proteins were annotated according to Swiss-Prot annotation, SOSUI prediction software and the literature. Proteins containing transmembrane domains, secreted proteins and proteins annotated as cell surface proteins by either Swiss-Prot or the literature were all considered cell surface proteins. A gene ontology (GO) analysis was performed using the DAVID software and database [23], [24]. Tissue specificity of the surface proteins was annotated according to UniProt annotations.

## Supporting Information

Table S1 A list of cell surface proteins on hES cells identified in this study.
(DOC)

Table S2 A list of cell surface proteins on hSperm cells identified in this study.
(DOC)
