# Supplementary material for: Proteomic Analyses Reveal Common Promiscuous Patterns of Cell Surface Proteins on Human Embryonic Stem Cells and Sperms
Source: PLoS One. 2011 May 3;6(5):e19386. doi: 10.1371/journal.pone.0019386 (PMC3086920; doi:10.1371/journal.pone.0019386)
Supplement: Table S1 — A list of cell surface proteins on hES cells identified in this study. (DOC) [file pone.0019386.s001.doc]

Table S1: hES cell surface proteins.

| **Protein Name** | **NCBI protein GI** |
| --- | --- |
| 1-acylglycerol-3-phosphate O-acyltransferase 5 | 61743952 |
| 5-hydroxytryptamine (serotonin) receptor 1D | 4504535 |
| 5T4 oncofetal trophoblast glycoprotein | 5729718 |
| a disintegrin and metalloproteinase domain 15 isoform 1 prepro | 46909592 |
| a disintegrin-like and metalloprotease (reprolysin type) with | 41281882 |
| AAA-ATPase TOB3 | 75677353 |
| aarF domain containing kinase 1 | 40254938 |
| aarF domain containing kinase 2 | 32261307 |
| acetyl-Coenzyme A carboxylase alpha isoform 1 | 38679960 |
| achalasia, adrenocortical insufficiency, alacrimia (Allgrove, | 12962937 |
| acid sphingomyelinase-like phosphodiesterase 3B isoform 1 | 57242798 |
| activated leukocyte cell adhesion molecule | 68163411 |
| activating NK receptor precursor | 16418407 |
| activin A receptor, type IIA precursor | 4501897 |
| acyl-CoA synthetase long-chain family member 3 | 42794752 |
| acyl-CoA synthetase long-chain family member 4 isoform 2 | 12669909 |
| acyl-Coenzyme A dehydrogenase, very long chain isoform 1 | 4557235 |
| acyl-malonyl condensing enzyme | 16876447 |
| ADAM metallopeptidase domain 10 | 4557251 |
| ADAM metallopeptidase domain 12 isoform 2 preproprotein | 73747887 |
| ADAM metallopeptidase domain 17 preproprotein | 73747889 |
| ADAM metallopeptidase domain 2 proprotein | 55743080 |
| ADAM metallopeptidase domain 21 preproprotein | 11497040 |
| ADAM metallopeptidase domain 22 isoform 4 preproprotein | 4757722 |
| ADAM metallopeptidase domain 23 preproprotein | 4501913 |
| ADAM metallopeptidase domain 28 isoform 1 preproprotein | 98985828 |
| ADAM metallopeptidase domain 29 preproprotein | 73765552 |
| ADAM metallopeptidase domain 30 preproprotein | 31881770 |
| ADAM metallopeptidase with thrombospondin type 1 motif, 12 | 51558724 |
| ADAM metallopeptidase with thrombospondin type 1 motif, 17 | 110611170 |
| ADAM metallopeptidase with thrombospondin type 1 motif, 5 | 195539372 |
| ADAM metallopeptidase with thrombospondin type 1 motif, 6 | 64276808 |
| ADAM metallopeptidase with thrombospondin type 1 motif, 7 | 38683827 |
| ADAM metallopeptidase with thrombospondin type 1 motif, 8 | 153792351 |
| ADAM metallopeptidase with thrombospondin type 1 motif, 9 | 33624896 |
| adenylate cyclase 3 | 148536830 |
| adenylate cyclase 4 | 24497587 |
| adenylate cyclase 6 isoform a | 10181096 |
| adenylate cyclase 8 | 4557257 |
| adenylate cyclase 9 | 50959205 |
| pituitary adenylate cyclase-activating polypeptide precursor | 153266795 |
| adipocyte-specific adhesion molecule | 13376115 |
| matrix-remodeling-associated protein 5 precursor | 139948432 |
| afamin precursor | 4501987 |
| A-gamma globin | 28302131 |
| alanine-glyoxylate aminotransferase | 4557289 |
| albumin precursor | 4502027 |
| ALEX2 protein | 29540562 |
| alpha 1 type I collagen preproprotein | 110349772 |
| collagen alpha-1(II) chain isoform 2 precursor | 111118974 |
| alpha 1 type IX collagen isoform 2 precursor | 73486664 |
| alpha 1 type V collagen preproprotein | 89276751 |
| alpha 1 type VII collagen precursor | 4502961 |
| alpha 1 type XI collagen isoform B preproprotein | 98985810 |
| alpha 1 type XIX collagen precursor | 47778921 |
| alpha 1 type XV collagen precursor | 116008152 |
| alpha 1 type XVI collagen precursor | 100913220 |
| alpha 1 type XVIII collagen isoform 3 precursor | 206597445 |
| alpha 1,2-mannosidase | 218749883 |
| alpha 1,4-galactosyltransferase | 8392830 |
| alpha 1B-glycoprotein | 21071030 |
| alpha 2 globin | 4504345 |
| alpha 2 type I collagen | 48762934 |
| alpha 2 type V collagen preproprotein | 89363017 |
| alpha 2 type VI collagen isoform 2C2a precursor | 115527070 |
| alpha 3 type IX collagen | 119508426 |
| alpha 3 type VI collagen isoform 2 precursor | 240255542 |
| alpha 3 type VI collagen isoform 4 precursor | 240255535 |
| alpha 4 type IV collagen precursor | 116256356 |
| alpha 5 type IV collagen isoform 2, precursor | 15890086 |
| alpha2,3-sialyltransferase VI | 5174697 |
| alpha-2A-adrenergic receptor | 194353970 |
| alpha-2-glycoprotein 1, zinc | 4502337 |
| alpha-2-plasmin inhibitor | 11386143 |
| amphoterin induced gene 2 | 32469517 |
| anaplastic lymphoma kinase Ki-1 | 29029632 |
| angiopoietin 1 | 20532340 |
| angiopoietin-like 1 precursor | 4757752 |
| angiopoietin-like 2 precursor | 6912236 |
| angiopoietin-like 3 precursor | 7656888 |
| angiopoietin-like 4 protein isoform a precursor | 21536398 |
| angiotensin I converting enzyme 2 precursor | 11225609 |
| angiotensin I converting enzyme isoform 1 precursor | 4503273 |
| angiotensin II receptor, type 1 | 6715583 |
| annexin 5 | 4502107 |
| annexin A2 isoform 2 | 4757756 |
| annexin A3 | 4826643 |
| anti-Mullerian hormone receptor, type II | 10198656 |
| apolipoprotein A-I preproprotein | 4557321 |
| apolipoprotein B precursor | 105990532 |
| apolipoprotein B-100 receptor | 119220598 |
| apolipoprotein C-III precursor | 4557323 |
| apolipoprotein C-IV | 4502161 |
| apolipoprotein D precursor | 4502163 |
| apolipoprotein E precursor | 4557325 |
| apolipoprotein L1 isoform a precursor | 21735614 |
| apolipoprotein L2 | 22035653 |
| archaemetzincins-2 isoform 1 | 75812966 |
| arginine-rich, mutated in early stage tumors | 54873600 |
| arginyl aminopeptidase (aminopeptidase B) | 40316915 |
| ATP binding cassette, sub-family A (ABC1), member 13 | 31657092 |
| ATP binding cassette, sub-family A (ABC1), member 13 | 31657092 |
| ATP citrate lyase isoform 1 | 38569421 |
| ATP synthase mitochondrial F1 complex assembly factor 2 | 21735485 |
| ATP synthase, H+ transporting, mitochondrial F0 complex, subunit d | 5453559 |
| ATP synthase, H+ transporting, mitochondrial F0 complex, subunit F6 | 18644883 |
| ATP synthase, H+ transporting, mitochondrial F0 complex, subunit s (factor B) | 51558774 |
| ATP synthase, H+ transporting, mitochondrial F0 complex, subunit E | 6005717 |
| ATP synthase, H+ transporting, mitochondrial F1 complex, alpha subunit 1, cardiac muscle | 50345984 |
| ATP synthase, H+ transporting, mitochondrial F1 complex, beta polypeptide | 32189394 |
| ATP synthase, H+ transporting, mitochondrial F1 complex, delta subunit | 4502297 |
| ATP synthase, H+ transporting, mitochondrial F1 complex, gamma polypeptide 1 | 50345988 |
| ATPase type 13A1 | 170016077 |
| ATPase type 13A4 | 66932949 |
| ATPase type 13A5 | 66730421 |
| ATPase, aminophospholipid transporter (APLT), class I, type 8A | 17978471 |
| ATPase, aminophospholipid transporter-like, Class I, type 8A, | 117168245 |
| ATPase, Ca++ transporting, fast twitch 1 isoform a | 27886529 |
| ATPase, Class I, type 8B, member 1 | 5031697 |
| ATPase, Class I, type 8B, member 2 isoform b | 55743077 |
| ATPase, Class V, type 10D | 222352161 |
| ATPase, Class VI, type 11B | 62632750 |
| ATPase, H+ transporting, lysosomal 50/57kDa, V1 subunit H | 47717100 |
| ATPase, H+ transporting, lysosomal 70kD, V1 subunit A | 19913424 |
| ATPase, H+ transporting, lysosomal accessory protein 1 precurs | 17136148 |
| ATPase, H+ transporting, lysosomal accessory protein 2 | 15011918 |
| ATPase, H+ transporting, lysosomal, V0 subunit d1 | 19913432 |
| ATPase, H+ transporting, lysosomal, V0 subunit d1 | 19913432 |
| ATPase, H+/K+ exchanging, beta polypeptide | 4557339 |
| ATPase, H+/K+ transporting, nongastric, alpha polypeptide | 83700225 |
| ATP-binding cassette protein C12 | 89111135 |
| ATP-binding cassette, sub-family A , member 5 | 27262624 |
| ATP-binding cassette, sub-family A member 1 | 21536376 |
| ATP-binding cassette, sub-family A member 3 | 116734710 |
| ATP-binding cassette, sub-family A member 4 | 105990541 |
| ATP-binding cassette, sub-family A, member 10 | 153792144 |
| ATP-binding cassette, sub-family A, member 12 isoform b | 27881501 |
| ATP-binding cassette, sub-family A, member 9 | 27477115 |
| ATP-binding cassette, sub-family B (MDR/TAP), member 11 | 21536378 |
| ATP-binding cassette, sub-family B, member 10 | 171184400 |
| ATP-binding cassette, subfamily B, member 4 isoform A | 4505771 |
| ATP-binding cassette, sub-family C (CFTR/MRP), member 2 | 4557481 |
| ATP-binding cassette, sub-family C, member 5 isoform 1 | 66529005 |
| ATP-binding cassette, sub-family C, member 9 isoform SUR2B | 110832837 |
| ATP-binding cassette, sub-family D, member 2 | 9945308 |
| ATP-binding cassette, sub-family D, member 3 | 4506341 |
| attractin-like 1 | 46410931 |
| B- and T-lymphocyte attenuator isoform 1 | 145580621 |
| basal cell adhesion molecule isoform 2 precursor | 61742797 |
| basigin isoform 2 | 38372925 |
| basigin isoform 4 | 38372923 |
| B-cell receptor-associated protein 31 | 32171186 |
| BCSC-1 isoform 1 | 38195080 |
| beta 3-glycosyltransferase-like | 154689817 |
| beta adrenergic receptor kinase 1 | 148539876 |
| beta(1,6)-N-acetylglucosaminyltransferase V isoform 1 | 39812181 |
| beta-1,3-glucuronyltransferase 2 | 18152775 |
| beta-1,4-mannosyltransferase | 41350216 |
| beta1,4-N-acetylgalactosaminyltransferases IV | 40789265 |
| beta-2-microglobulin precursor | 4757826 |
| beta-galactoside alpha-2,6-sialyltransferase II | 26190610 |
| beta-galactoside-binding lectin precursor | 4504981 |
| beta-neoendorphin-dynorphin preproprotein | 13270473 |
| biglycan preproprotein | 4502403 |
| BM88 antigen | 30795225 |
| BMP-binding endothelial regulator precursor protein | 37693519 |
| bone marrow stromal cell antigen 2 | 4757876 |
| bone morphogenetic protein 1 isoform 1, precursor | 4502421 |
| bone morphogenetic protein 1 isoform 3, precursor | 5453579 |
| bone morphogenetic protein 10 preproprotein | 7656928 |
| bone morphogenetic protein 6 precursor | 4502425 |
| brain adenylate cyclase 1 | 31083193 |
| brain link protein 2 | 30794472 |
| brain-specific angiogenesis inhibitor 2 | 115387099 |
| brain-specific angiogenesis inhibitor 3 | 4502359 |
| brevican isoform 1 | 38372935 |
| BRI3-binding protein | 19923665 |
| bromodomain and WD repeat domain containing 2 | 13324688 |
| brother of CDO | 15147240 |
| butyrophilin, subfamily 2, member A1 isoform 2 precursor | 17975772 |
| butyrophilin, subfamily 2, member A2 isoform a | 6453813 |
| butyrophilin, subfamily 3, member A3 isoform a | 5901908 |
| butyrophilin-like 9 | 60460897 |
| C1q and tumor necrosis factor related protein 4 | 148233912 |
| C1q domain containing 1 isoform 2 | 23503235 |
| C3 and PZP-like alpha-2-macroglobulin domain-containing protein 8 | 118600977 |
| cadherin 1, type 1 preproprotein | 4757960 |
| cadherin 10, type 2 preproprotein | 16306530 |
| cadherin 11, type 2 preproprotein | 16306532 |
| cadherin 13 preproprotein | 4502719 |
| cadherin 15 preproprotein | 4826669 |
| cadherin 16 precursor | 4757956 |
| cadherin 17 precursor | 221316593 |
| cadherin 2, type 1 preproprotein | 14589889 |
| cadherin 3, type 1 preproprotein | 14589891 |
| cadherin 4, type 1 preproprotein | 14589893 |
| cadherin 6, type 2 preproprotein | 4826673 |
| cadherin EGF LAG seven-pass G-type receptor 1 | 7656967 |
| cadherin EGF LAG seven-pass G-type receptor 2 | 13325064 |
| cadherin EGF LAG seven-pass G-type receptor 3 | 145309304 |
| cadherin related 23 isoform 1 precursor | 189571674 |
| cadherin related 23 isoform 2 precursor | 16507964 |
| calcium activated chloride channel 2 | 5729769 |
| calcium channel, voltage-dependent, alpha 1F subunit | 53832007 |
| calcium channel, voltage-dependent, alpha 1H subunit isoform a | 53832009 |
| calcium channel, voltage-dependent, alpha 2/delta subunit 1 | 54112390 |
| calcium channel, voltage-dependent, alpha 2/delta subunit 2 | 54112392 |
| calcium channel, voltage-dependent, beta 1 subunit isoform 2 | 40804470 |
| calcium channel, voltage-dependent, beta 3 subunit | 40316928 |
| calcium-activated potassium channel beta 3 subunit isoform a | 25952099 |
| caldesmon 1 isoform 2 | 4826657 |
| calnexin precursor | 10716563 |
| carbohydrate (chondroitin) synthase 1 | 31542309 |
| carbohydrate (N-acetylgalactosamine 4-0) sulfotransferase 8 | 20127609 |
| carbohydrate (N-acetylglucosamine 6-O) sulfotransferase 6 | 11055976 |
| carbonic anhydrase IX precursor | 169636420 |
| carbonic anhydrase XI precursor | 9951923 |
| carcinoembryonic antigen-related cell adhesion molecule 6 | 40255013 |
| carnitine palmitoyltransferase 1A isoform 1 | 73623030 |
| cartilage oligomeric matrix protein precursor | 40217843 |
| cat eye syndrome critical region protein 1 isoform a precursor | 29029550 |
| cation-dependent mannose-6-phosphate receptor precursor | 4505061 |
| cauxin | 21450749 |
| CD109 | 115529484 |
| CD163 antigen isoform b | 44889963 |
| CD19 antigen | 91105174 |
| CD1A antigen precursor | 110618224 |
| CD2 antigen (p50), sheep red blood cell receptor | 156071472 |
| CD200 antigen isoform b | 90903245 |
| CD22 antigen | 157168355 |
| CD276 antigen isoform a | 67188443 |
| CD27-binding (Siva) protein isoform 1 | 11277468 |
| CD2-associated protein | 11321634 |
| CD44 antigen isoform 3 precursor | 48255939 |
| CD5 antigen (p56-62) | 166197668 |
| CD58 antigen, (lymphocyte function-associated antigen 3) | 4502677 |
| CD6 antigen | 194018748 |
| CD79B antigen isoform 1 precursor | 11038674 |
| CD81 antigen | 4757944 |
| CD9 antigen | 4502693 |
| CD97 antigen isoform 2 precursor | 17978489 |
| CD99 antigen | 4505183 |
| CD99 antigen-like 2 isoform E4 | 41281656 |
| CDC14 homolog A isoform 2 | 15451931 |
| choline transporter-like protein 1 | 18034692 |
| cell recognition molecule Caspr2 precursor | 7662350 |
| cell recognition molecule CASPR3 | 47519929 |
| ceramide kinase isoform a | 20336726 |
| cerberus 1 | 4885135 |
| Charcot-Leyden crystal protein | 20357559 |
| chemokine (C-C motif) ligand 20 | 4759076 |
| chemokine (C-C motif) receptor-like 2 | 68215224 |
| chemokine (C-X-C motif) ligand 12 | 10834988 |
| chemokine (C-X-C motif) ligand 2 | 4504155 |
| chemokine (C-X-C motif) ligand 5 precursor | 4506849 |
| chemokine (C-X-C motif) ligand 6 | 4506851 |
| chemokine-like factor superfamily 1 isoform 12 | 31563424 |
| chemokine-like factor superfamily 2 | 21389567 |
| chemokine-like factor superfamily 4 isoform 2 | 31657096 |
| chloride channel 6 isoform ClC-6a | 4502873 |
| cholecystokinin preproprotein | 4502605 |
| cholesteryl ester transfer protein, plasma precursor | 169636439 |
| cholinergic receptor, muscarinic 1 | 37622910 |
| cholinergic receptor, muscarinic 2 | 54792115 |
| cholinergic receptor, nicotinic, alpha 9 precursor | 70995130 |
| cholinergic receptor, nicotinic, alpha polypeptide 5 | 33589827 |
| chondrocyte protein with a poly-proline region | 224994160 |
| chondroitin beta1,4 N-acetylgalactosaminyltransferase 2 | 24429592 |
| chondroitin sulfate glucuronyltransferase | 48717495 |
| chondroitin sulfate proteoglycan 2 (versican) | 21361116 |
| chondroitin sulfate proteoglycan 6 (bamacan) | 4885399 |
| chondroitin sulfate synthase 3 | 74271903 |
| chordin-like 1 | 34147715 |
| chromosome 22 open reading frame 5 | 63259329 |
| chromosome 6 open reading frame 10 | 116256485 |
| chromosome 6 open reading frame 71 | 42794271 |
| chromosome 9 open reading frame 36 | 153791826 |
| chromosome 9 open reading frame 79 | 155029550 |
| ciliary neurotrophic factor | 4758020 |
| class-I MHC-restricted T cell associated molecule | 51593098 |
| claudin 12 | 6912312 |
| claudin 6 | 153792768 |
| coagulation factor C homolog, cochlin precursor | 4758022 |
| coagulation factor II precursor | 4503635 |
| coagulation factor V precursor | 105990535 |
| coagulation factor VII precursor, isoform a | 4503645 |
| coagulation factor VIII isoform a precursor | 4503647 |
| coagulation factor XIII A1 subunit precursor | 119395709 |
| coagulation factor XIII B subunit precursor | 110611237 |
| codanin 1 | 57222570 |
| coiled-coil domain containing 109A | 24308400 |
| collagen triple helix repeat containing 1 | 19923989 |
| collagen, type VI, alpha 1 precursor | 87196339 |
| collagen, type X, alpha 1 precursor | 18105032 |
| collagen, type XXI, alpha 1 precursor | 18780273 |
| collagen, type XXII, alpha 1 | 40805823 |
| collagen, type XXIII, alpha 1 | 29725624 |
| collagen alpha-1(XXIV) chain precursor | 115392133 |
| collagen, type XXV, alpha 1 isoform 2 | 38570073 |
| collagen, type XXVII, alpha 1 | 32140760 |
| collectin sub-family member 12 isoform I | 18641360 |
| colony stimulating factor 1 receptor precursor | 27262659 |
| colony stimulating factor 2 receptor, beta, low-affinity | 4559408 |
| complement component 1 inhibitor precursor | 73858568 |
| complement component 1, q subcomponent binding protein precursor | 4502491 |
| complement component 1, s subcomponent | 4502495 |
| complement component 2 precursor | 14550407 |
| complement component 4A preproprotein | 67190748 |
| complement component 7 precursor | 45580688 |
| complement component 8, beta polypeptide preproprotein | 4557391 |
| complement component 9 | 4502511 |
| complement factor B preproprotein | 67782358 |
| complement factor H isoform b precursor | 62739188 |
| connexin 43 | 4504001 |
| gap junction delta-4 protein | 145699105 |
| contactin 2 precursor | 4827022 |
| contactin 3 | 75709184 |
| contactin 4 isoform a precursor | 28373122 |
| contactin 4 isoform c precursor | 28373126 |
| corticotropin releasing hormone binding protein | 47080099 |
| cortistatin preproprotein | 41327683 |
| COX15 homolog isoform 1 precursor | 17921985 |
| coxsackie virus and adenovirus receptor precursor | 4503173 |
| crumbs homolog 1 precursor | 41327708 |
| crumbs homolog 2 | 112420992 |
| C-terminal binding protein 1 isoform 2 | 61743967 |
| CTL2 protein | 222831612 |
| C-type lectin domain family 14, member A | 28269707 |
| C-type lectin, superfamily member 13 | 47778940 |
| C-type lectin, superfamily member 6 isoform 3 | 148536836 |
| CUB and Sushi multiple domains 1 | 259013213 |
| CUB and Sushi multiple domains 3 isoform 3 | 205277354 |
| CUB domain-containing protein 1 isoform 1 | 30410805 |
| cyclic nucleotide gated channel alpha 3 | 4502917 |
| cyclic nucleotide gated channel beta 3 | 116642889 |
| cysteine-rich motor neuron 1 | 10092639 |
| cystic fibrosis transmembrane conductance regulator | 90421313 |
| cytochrome c oxidase subunit VIIc precursor | 4502993 |
| cytochrome c-1 | 21359867 |
| cytoskeleton-associated protein 4 | 19920317 |
| cytosolic ovarian carcinoma antigen 1 isoform a | 32528293 |
| dachsous 1 precursor | 16933557 |
| death-inducing-protein | 67763814 |
| defender against cell death 1 | 4503253 |
| defensin, beta 130 | 83699414 |
| degenerative spermatocyte homolog 1, lipid desaturase | 4505193 |
| netrin receptor DCC precursor | 110431348 |
| delta-like 4 protein precursor | 9506545 |
| dermatan 4 sulfotransferase 1 | 18497304 |
| dermcidin preproprotein | 16751921 |
| desert hedgehog preproprotein | 19482158 |
| desmocollin 1 isoform Dsc1b preproprotein | 4826702 |
| desmocollin 2 isoform Dsc2b preproprotein | 13435366 |
| desmoglein 1 preproprotein | 119703744 |
| desmoglein 2 preproprotein | 116534898 |
| DHHC1 protein | 7706133 |
| dipeptidyl peptidase 10 isoform long | 52426756 |
| dipeptidylpeptidase IV | 18765694 |
| discoidin domain receptor family, member 1 isoform c | 83977452 |
| discoidin domain receptor family, member 2 precursor | 62420884 |
| discoidin, CUB and LCCL domain containing 1 | 27735143 |
| dispatched A | 25952134 |
| dispatched B | 25121980 |
| alpha-1,2-mannosyltransferase ALG9 isoform a | 118026921 |
| dolichyl-diphosphooligosaccharide-protein glycosyltransferase | 20070197 |
| dolichyl-phosphate mannosyltransferase polypeptide 3 isoform | 19424120 |
| dopamine receptor interacting protein | 119943096 |
| doublecortin and CaM kinase-like 1 | 4758128 |
| Down syndrome cell adhesion molecule isoform CHD2-42 precursor | 20127422 |
| Down syndrome cell adhesion molecule like 1 | 21359935 |
| down-regulated in adenoma protein | 4557535 |
| dysferlin | 4503431 |
| dystroglycan 1 precursor | 294997282 |
| ectonucleoside triphosphate diphosphohydrolase 7 | 9966821 |
| ectonucleotide pyrophosphatase/phosphodiesterase 3 | 111160296 |
| ectonucleotide pyrophosphatase/phosphodiesterase 4 (putative f | 7662358 |
| ectonucleotide pyrophosphatase/phosphodiesterase 6 | 23503267 |
| ectonucleotide pyrophosphatase/phosphodiesterase 7 | 45545421 |
| EGF-like-domain, multiple 4 | 145701025 |
| EGF-like-domain, multiple 7 | 7705889 |
| elastin | 126352440 |
| elastin microfibril interfacer 1 | 5901944 |
| elastin microfibril interfacer 2 | 60498978 |
| elastin microfibril interfacer 3 | 45433501 |
| electron-transferring-flavoprotein dehydrogenase | 119703746 |
| enamelin | 189491628 |
| endoglin precursor | 4557555 |
| endoglycan | 7657465 |
| endomucin | 20070284 |
| endoplasmic reticulum to nucleus signalling 1 isoform 1 | 153946421 |
| endothelial cell growth factor 1 (platelet-derived) | 4503445 |
| lysophosphatidic acid receptor 3 | 6912348 |
| sphingosine-1-phosphate receptor 3 | 38788193 |
| endothelin converting enzyme 1 | 4503443 |
| endothelin converting enzyme 2 isoform A | 153945761 |
| endothelin converting enzyme-like 1 | 157426891 |
| EPH receptor A8 isoform 2 precursor | 55770892 |
| ephrin receptor EphA1 | 221316650 |
| ephrin receptor EphA2 | 32967311 |
| ephrin receptor EphA4 | 4758280 |
| ephrin receptor EphB4 precursor | 32528301 |
| ephrin-B1 precursor | 4758248 |
| epidermal growth factor receptor isoform a | 29725609 |
| erbB-2 isoform b | 54792098 |
| erbB-3 isoform 1 precursor | 54792100 |
| erythrocyte membrane protein band 4.2 | 166362735 |
| transmembrane channel-like protein 6 | 187608784 |
| exostoses (multiple)-like 1 | 112382285 |
| F11 receptor isoform a precursor | 21464113 |
| factor for adipocyte differentiation 158 | 19923729 |
| FAD-dependent oxidoreductase domain containing 1 | 8923708 |
| family 3, member A protein | 283945606 |
| family with sequence similarity 20, member B | 7662150 |
| family with sequence similarity 62 (C2 domain containing), member B | 45387945 |
| FAT tumor suppressor 1 precursor | 66346693 |
| FAT tumor suppressor homolog 4 | 165932370 |
| fatty acid desaturase 2 | 4758334 |
| fatty acid synthase | 41872631 |
| Fc alpha receptor isoform f | 19743865 |
| Fc fragment of IgE, high affinity I, receptor for, gamma polypeptide | 4758344 |
| Fc receptor-like 4 | 14550416 |
| fibrillin 1 precursor | 281485550 |
| fibrillin 2 precursor | 66346695 |
| fibrillin 3 precursor | 56237021 |
| fibroblast growth factor 20 | 9789947 |
| fibroblast growth factor receptor 1 isoform 2 precursor | 13186251 |
| fibroblast growth factor receptor 2 isoform 2 precursor | 221316638 |
| fibroblast growth factor receptor-like 1 precursor | 51988914 |
| fibrocystin L | 126116589 |
| fibromodulin precursor | 71040111 |
| fibronectin 1 isoform 3 preproprotein | 16933542 |
| fibronectin leucine rich transmembrane protein 1 | 34577057 |
| fibronectin leucine rich transmembrane protein 3 precursor | 7019383 |
| ficolin 2 isoform a precursor | 61744445 |
| flavin containing monooxygenase 1 | 4503755 |
| fms-related tyrosine kinase 4 isoform 2 | 103472027 |
| follistatin-like 1 precursor | 5901956 |
| follistatin-like 4 | 54792136 |
| follistatin-like 5 | 190358520 |
| Fraser syndrome 1 | 256000767 |
| frizzled 1 | 4503825 |
| frizzled 10 | 6005762 |
| frizzled 4 | 22547161 |
| frizzled 5 | 27894385 |
| fucosyltransferase 11 (alpha (1,3) fucosyltransferase) | 145580617 |
| fucosyltransferase 7 (alpha (1,3) fucosyltransferase) | 4758406 |
| furin preproprotein | 4505579 |
| FXYD domain-containing ion transport regulator 6 | 11612655 |
| G protein-coupled receptor 101 | 16876435 |
| G protein-coupled receptor 107 | 56711308 |
| G protein-coupled receptor 126 beta 1 | 50355941 |
| G protein-coupled receptor 139 | 50897278 |
| G protein-coupled receptor 147 | 11545887 |
| G protein-coupled receptor 149 | 84662753 |
| G protein-coupled receptor 152 | 46243671 |
| G protein-coupled receptor 157 | 93204873 |
| G protein-coupled receptor 18 | 15029528 |
| G protein-coupled receptor 45 | 31083315 |
| melatonin-related receptor | 150170722 |
| G protein-coupled receptor 51 | 40255245 |
| G protein-coupled receptor 64 | 119943129 |
| G protein-coupled receptor 78 | 36951034 |
| G protein-coupled receptor, family C, group 5, member A | 4506403 |
| G protein-coupled receptor, family C, group 6, member A | 112807234 |
| G4 protein | 57165366 |
| galactose-3-O-sulfotransferase 2 | 124256491 |
| galactosidase, beta 1-like | 40255043 |
| galectin 3 | 115430223 |
| galectin 8 isoform b | 42544191 |
| GalNAc alpha-2, 6-sialyltransferase I | 21264332 |
| gamma-aminobutyric acid (GABA) A receptor, alpha 5 precursor | 4503861 |
| gamma-aminobutyric acid (GABA) A receptor, beta 1 precursor | 194097327 |
| gamma-aminobutyric acid (GABA) B receptor 1 isoform b precursor | 11497612 |
| gamma-aminobutyric acid A receptor, alpha 3 precursor | 4557603 |
| gamma-aminobutyric acid A receptor, alpha 4 precursor | 34452723 |
| gamma-aminobutyric acid A receptor, gamma 1 precursor | 31742490 |
| gamma-glutamyltransferase 2 | 62079287 |
| ganglioside-induced differentiation-associated protein 1 | 108773797 |
| gap junction protein, alpha 7, 45kDa (connexin 45) | 69122473 |
| gap junction protein, alpha 8, 50kDa (connexin 50) | 55953076 |
| GCN1 general control of amino-acid synthesis 1-like 1 | 54607053 |
| germ cell associated 1 isoform 2 | 123317861 |
| glial cell derived neurotrophic factor isoform 1 preproprotein | 4503975 |
| glucosaminyl (N-acetyl) transferase 2, I-branching enzyme | 85790495 |
| glucose phosphate isomerase | 18201905 |
| glutamate receptor 7 precursor | 28605145 |
| glutamate receptor KA1 precursor | 29029595 |
| glutamate receptor, ionotrophic | 164419734 |
| glutamate receptor, ionotropic, delta 1 | 55770852 |
| glutamate receptor, ionotropic, kainate 1 isoform 2 precursor | 28416444 |
| glutamate receptor, ionotropic, N-methyl-D-aspartate 3B | 62988346 |
| glutamate receptor, metabotropic 4 | 4504141 |
| glutamate receptor, metabotropic 5 precursor | 4504143 |
| glutamate receptor, metabotropic 6 precursor | 187960067 |
| glutamate receptor, metabotropic 8 precursor | 4504149 |
| glutamyl aminopeptidase (aminopeptidase A) | 132814467 |
| glutathione reductase | 50301238 |
| glycerophosphodiester phosphodiesterase domain containing 5 | 189571657 |
| glycophorin C isoform 1 | 4504229 |
| glycoprotein M6A isoform 3 | 42476105 |
| glycoprotein M6B isoform 1 | 50263048 |
| glycoprotein, synaptic 2 | 24475816 |
| glypican 1 precursor | 167001141 |
| glypican 2 | 22749459 |
| glypican 3 | 4758462 |
| glypican 4 | 21614525 |
| glypican 6 precursor | 5031719 |
| golgi apparatus protein 1 | 54633312 |
| Golgi autoantigen, golgin subfamily a, 5 | 30260188 |
| golgi autoantigen, golgin subfamily b, macrogolgin | 148596984 |
| golgi phosphoprotein 2 | 29550838 |
| golgi phosphoprotein 4 | 7657138 |
| golgi reassembly stacking protein 2 | 29826294 |
| golgin 97 | 4504063 |
| GPI ethanolamine phosphate transferase 2 isoform 2 | 187608438 |
| GPI-anchored metastasis-associated protein homolog | 93004088 |
| phosphatidylinositol-glycan biosynthesis class X protein isoform 2 precursor | 261490706 |
| GPR158-like 1 | 93352554 |
| G-protein coupled receptor 112 | 59710093 |
| G-protein coupled receptor 115 | 116517328 |
| G-protein coupled receptor 116 | 148719673 |
| G-protein coupled receptor 173 | 9507143 |
| granzyme A precursor | 5453676 |
| granzyme H | 15529990 |
| GREB1 protein isoform a | 23397642 |
| growth arrest-specific 1 | 167466169 |
| growth differentiation factor 6 | 48475062 |
| growth differentiation factor 7 preproprotein | 61835232 |
| growth differentiation factor 8 | 4885259 |
| growth hormone 1 isoform 4 | 13027818 |
| growth hormone inducible transmembrane protein | 118200356 |
| GTPase, IMAP family member 1 | 18594498 |
| guanylate cyclase 2C (heat stable enterotoxin receptor) | 222080083 |
| guanylate cyclase 2D, membrane (retina-specific) | 4504217 |
| retinal guanylyl cyclase 2 precursor | 134152694 |
| heat shock 105kD | 42544159 |
| heat shock 10kDa protein 1 (chaperonin 10) | 4504523 |
| heat shock 70kD protein 12B | 31317303 |
| heat shock 70kDa protein 1A | 194248072 |
| heat shock 70kDa protein 1-like | 124256496 |
| heat shock 70kDa protein 2 | 13676857 |
| heat shock 70kDa protein 4-like | 31541941 |
| heat shock 70kDa protein 5 (glucose-regulated protein, 78kDa) | 16507237 |
| heat shock 70kDa protein 6 (HSP70B') | 34419635 |
| heat shock 70kDa protein 8 isoform 1 | 5729877 |
| heat shock 70kDa protein 9B precursor | 24234688 |
| heat shock 90kDa protein 1, beta | 20149594 |
| heat shock protein 90kDa alpha (cytosolic), class A member 1 I | 153792590 |
| hedgehog-interacting protein | 20143973 |
| hematopoietic protein 1 | 34485727 |
| hemicentin 1 | 118572606 |
| hemochromatosis protein isoform 1 precursor | 4504377 |
| hemopexin | 11321561 |
| heparan sulfate 2-O-sulfotransferase 1 | 6912420 |
| heparan sulfate proteoglycan 2 | 126012571 |
| heparin cofactor II precursor | 73858566 |
| heparin-binding EGF-like growth factor | 4503413 |
| HGF activator preproprotein | 4504383 |
| HLA-B associated transcript 5 | 15100151 |
| HNK-1 sulfotransferase | 4758540 |
| hornerin | 57864582 |
| HtrA serine peptidase 4 | 24308541 |
| human epididymis-specific 3 beta precursor | 11641279 |
| hyaluronan and proteoglycan link protein 3 | 30102948 |
| hyaluronan synthase 1 | 167466171 |
| hyaluronan mediated motility receptor isoform c | 217416398 |
| hyaluronoglucosaminidase 1 isoform 1 | 24497564 |
| hyaluronoglucosaminidase 4 | 166235888 |
| hydroxysteroid (17-beta) dehydrogenase 13 | 210032110 |
| hyperpolarization activated cyclic nucleotide-gated potassium | 116325989 |
| potassium/sodium hyperpolarization-activated cyclic nucleotide-gated channel 2 | 156071470 |
| protein furry homolog | 117606355 |
| coiled-coil domain-containing protein 136 | 115511012 |
| leucine-rich repeat and fibronectin type-III domain-containing protein 6 | 116268101 |
| hypothetical protein LOC115939 | 47777330 |
| nogo-B receptor precursor | 20270243 |
| hypothetical protein LOC116254 | 20302038 |
| putative sodium-coupled neutral amino acid transporter 10 isoform a | 83921602 |
| netrin-5 precursor | 24308065 |
| lysozyme g-like protein 1 precursor | 28372527 |
| fibulin-7 isoform 1 | 189491645 |
| transmembrane protein 182 precursor | 188528658 |
| mitochondrial sodium/hydrogen exchanger NHA2 | 47271479 |
| hypothetical protein LOC134145 | 154240720 |
| williams-Beuren syndrome chromosomal region 28 protein | 153281226 |
| hypothetical protein LOC136263 | 21687171 |
| putative transporter SVOPL isoform 2 | 33457322 |
| hypothetical protein LOC145407 | 56711320 |
| transmembrane and coiled-coil domain-containing protein 5A | 34303932 |
| germ cell-specific gene 1-like protein isoform 2 | 21389571 |
| hypothetical protein LOC147685 | 22748999 |
| transmembrane protein 190 precursor | 21040263 |
| WD repeat-containing protein 65 | 269847282 |
| tetratricopeptide repeat protein 30B | 125988411 |
| hypothetical protein LOC153830 | 21389515 |
| hypothetical protein LOC160335 | 22749211 |
| DENN domain-containing protein 5B | 122891862 |
| hypothetical protein LOC162073 | 77798169 |
| consortin isoform 1 | 213021160 |
| eucine-rich repeat neuronal protein 4 precursor | 188536110 |
| hypothetical protein LOC166929 | 22749271 |
| hypothetical protein LOC196446 | 32698898 |
| hypothetical protein LOC201158 | 205277366 |
| hypothetical protein LOC202018 | 130977756 |
| hypothetical protein LOC205717 | 114431248 |
| hypothetical protein LOC221786 | 21495178 |
| protein sel-1 homolog 3 | 154689719 |
| hypothetical protein LOC23302 | 54792094 |
| hypothetical protein LOC23732 | 115648140 |
| collagen alpha-5(VI) chain | 183583553 |
| hypothetical protein LOC256710 | 22749527 |
| hypothetical protein LOC25851 | 32698704 |
| hypothetical protein LOC25871 isoform a | 68299770 |
| transmembrane protein 186 | 218749843 |
| hypothetical protein LOC283417 | 93277105 |
| carboxylesterase 8 | 115529439 |
| transmembrane protein C16orf54 | 148233642 |
| hypothetical protein LOC284114 | 30425460 |
| hypothetical protein LOC285093 | 134288863 |
| hypothetical protein LOC286148 | 167466219 |
| protein SCAI isoform 1 | 116256473 |
| hypothetical protein LOC286464 | 27734891 |
| hypothetical protein LOC340061 | 38093659 |
| leucine-rich repeat, immunoglobulin-like domain and transmembrane domain-containing protein 3 | 193083136 |
| hypothetical protein LOC346689 | 38348332 |
| hypothetical protein LOC347365 | 38348336 |
| hypothetical protein LOC347862 | 32699045 |
| hypothetical protein LOC374819 | 75677612 |
| hypothetical protein LOC374872 | 38348368 |
| hypothetical protein LOC374977 | 223278410 |
| hypothetical protein LOC387104 | 59806361 |
| hypothetical protein LOC387597 | 40786420 |
| hypothetical protein LOC388595 | 61966731 |
| family with sequence similarity 69, member A | 165932391 |
| hypothetical protein LOC389763 | 48717285 |
| probable glutathione peroxidase 8 | 192455698 |
| hypothetical protein LOC51300 | 59710109 |
| hypothetical protein LOC54621 | 40254891 |
| GRAM domain-containing protein 1C isoform 1 | 187936935 |
| major facilitator superfamily domain-containing protein 6 | 144953907 |
| hypothetical protein LOC54947 | 47086907 |
| hypothetical protein LOC54964 | 20149646 |
| hypothetical protein LOC54978 | 31542711 |
| hypothetical protein LOC54991 | 192447436 |
| ecto-NOX disulfide-thiol exchanger 1 | 189027046 |
| hypothetical protein LOC55129 | 94536803 |
| serine palmitoyltransferase 3 | 119220554 |
| hypothetical protein LOC55379 | 40254924 |
| hypothetical protein LOC55780 | 62988331 |
| hypothetical protein LOC57003 | 171906582 |
| hypothetical protein LOC57020 | 31543159 |
| hypothetical protein LOC574028 | 68563517 |
| protein unc-79 homolog | 150456444 |
| hypothetical protein LOC57609 | 55749758 |
| hypothetical protein LOC57653 | 55741661 |
| hypothetical protein LOC57655 | 92110010 |
| hypothetical protein LOC57719 | 55741655 |
| hypothetical protein LOC60492 | 20149663 |
| hypothetical protein LOC60686 | 195233774 |
| fer-1-like protein 6 | 119120884 |
| hypothetical protein LOC79714 | 94536784 |
| hypothetical protein LOC79794 | 13376060 |
| hypothetical protein LOC79805 | 31982921 |
| hypothetical protein LOC79820 | 51339295 |
| coiled-coil domain-containing protein 102B | 148233522 |
| hypothetical protein LOC79883 | 226342931 |
| hypothetical protein LOC80018 | 38679914 |
| PGAP2-interacting protein | 156151386 |
| hypothetical protein LOC80167 | 89363030 |
| hypothetical protein LOC80208 | 93204888 |
| hypothetical protein LOC80228 | 14249538 |
| transmembrane protein 177 | 157419118 |
| hypothetical protein LOC84077 | 68163572 |
| hypothetical protein LOC84179 | 31542731 |
| hypothetical protein LOC84216 | 14149981 |
| hypothetical protein LOC84293 | 148596959 |
| hypothetical protein LOC84803 | 21362092 |
| transmembrane and TPR repeat-containing protein 4 isoform 1 | 118766330 |
| hypothetical protein LOC84910 | 14249524 |
| hypothetical protein LOC84975 | 283046669 |
| hypothetical protein LOC90231 | 25286703 |
| lipase maturation factor 2 | 255918129 |
| hypothetical protein LOC91775 | 21450781 |
| hypothetical protein LOC92126 | 23943787 |
| hypothetical protein LOC9671 | 55749667 |
| hypothetical protein LOC9703 | 57242774 |
| hypothetical protein LOC9813 | 7662160 |
| leucine-rich repeat-containing protein 37A precursor | 289547512 |
| immunoglobin superfamily, member 21 | 281306830 |
| immunoglobulin superfamily, member 1 isoform 1 | 45505167 |
| immunoglobulin superfamily, member 10 | 38490688 |
| immunoglobulin superfamily, member 2 | 157694489 |
| immunoglobulin superfamily, member 3 isoform 2 | 55953135 |
| immunoglobulin superfamily, member 4C | 21686977 |
| cell adhesion molecule 1 isoform 1 | 148664190 |
| immunoglobulin superfamily, member 8 | 16445029 |
| Indian hedgehog homolog | 119392086 |
| inducible T-cell co-stimulator precursor | 15029518 |
| inositol polyphosphate-5-phosphatase, 75kDa | 113722125 |
| insulin receptor | 119395736 |
| insulin-like growth factor 1 receptor precursor | 4557665 |
| insulin-like growth factor 2 receptor | 119964726 |
| integral membrane protein 1 | 22749415 |
| integral membrane protein 2A | 4758224 |
| integral membrane protein 2B | 11527402 |
| integrin alpha 3 isoform a precursor | 4504747 |
| integrin alpha 4 precursor | 67191027 |
| integrin alpha 5 precursor | 56237029 |
| integrin alpha 7 precursor | 222418611 |
| integrin alpha chain, alpha 6 | 119395742 |
| integrin alpha FG-GAP repeat containing 3 | 14042970 |
| integrin alpha M precursor | 88501734 |
| integrin alpha-V precursor | 4504763 |
| integrin beta 1 isoform 1A precursor | 19743813 |
| integrin beta 1 isoform 1C-2 precursor | 19743821 |
| integrin beta 4 isoform 1 precursor | 54607035 |
| integrin beta 4 isoform 3 precursor | 54607033 |
| integrin, alpha 8 | 49170034 |
| integrin, alpha D precursor | 62548866 |
| integrin, alpha E | 148728188 |
| integrin, beta 5 | 20127446 |
| integrin, beta 6 | 9625002 |
| integrin, beta 8 precursor | 4504779 |
| inter-alpha (globulin) inhibitor H4 | 31542984 |
| inter-alpha globulin inhibitor H2 polypeptide | 70778918 |
| intercellular adhesion molecule 1 precursor | 167466198 |
| intercellular adhesion molecule 3 precursor | 167466207 |
| interferon gamma receptor 1 | 4557880 |
| interferon induced transmembrane protein 1 (9-27) | 150010589 |
| interferon, alpha 1 | 13128950 |
| interferon, alpha 2 | 11067751 |
| interferon-alpha receptor 1 precursor | 46488932 |
| interleukin 1 receptor accessory protein isoform 2 | 19882209 |
| interleukin 12 receptor, beta 2 precursor | 4504643 |
| interleukin 12A precursor | 24430219 |
| interleukin 16 isoform 2 | 148833504 |
| interleukin 17 receptor E isoform 2 | 24430206 |
| interleukin 17 receptor E | 132014 |
| interleukin 17A receptor precursor | 23238208 |
| interleukin 17B receptor isoform 1 precursor | 27477074 |
| interleukin 17D precursor | 19923715 |
| interleukin 21 receptor precursor | 31083174 |
| interleukin 5 receptor, alpha isoform 1 precursor | 28559021 |
| interleukin 6 receptor isoform 2 precursor | 31317249 |
| interleukin 8 receptor beta | 4504683 |
| interphotoreceptor matrix proteoglycan 2 | 57242793 |
| patatin-like phospholipase domain containing 8 | 48525351 |
| jagged 1 precursor | 4557679 |
| JAW1-related protein isoform b | 148728182 |
| JM4 protein | 6005794 |
| junctional adhesion molecule 2 precursor | 10864029 |
| junctional adhesion molecule 3 precursor | 21361905 |
| kallikrein 11 isoform 1 preproprotein | 5803199 |
| kelch-like 11 | 8922528 |
| Kell blood group, metalloendopeptidase | 4557691 |
| keratocan | 5901992 |
| KIAA0319 | 134304840 |
| transmembrane protein 131-like isoform 2 | 187608809 |
| KIAA1913 | 29789291 |
| killer cell immunoglobulin-like receptor, two domains, long cytoplasmic tail, 5B; killer cell immunoglobulin-like receptor, two domains, long cytoplasmic tail, 5A | 11968154 |
| killer cell lectin-like receptor subfamily C, member 3 | 75709175 |
| kin of IRRE like | 166295173 |
| kin of IRRE like 3 | 26006461 |
| kinase D-interacting substance of 220 kDa | 55741641 |
| kinase insert domain receptor | 11321597 |
| kinectin 1 | 33620775 |
| klotho beta like | 28376633 |
| L1 cell adhesion molecule isoform 2 precursor | 13435353 |
| lactotransferrin | 54607120 |
| ladinin 1 | 20070205 |
| LAG1 longevity assurance homolog 2 isoform 1 | 32455256 |
| laminin alpha 2 subunit precursor | 28559088 |
| laminin alpha 3 subunit isoform 1 | 38045910 |
| laminin alpha 5 | 21264602 |
| laminin subunit beta 3 precursor | 62868215 |
| laminin, alpha 1 precursor | 38788416 |
| laminin, beta 1 precursor | 167614504 |
| laminin, beta 2 precursor | 119703755 |
| laminin, gamma 1 precursor | 145309326 |
| laminin, gamma 2 isoform a precursor | 157419138 |
| laminin, gamma 3 precursor | 110611156 |
| potassium large conductance calcium-activated channel, subfamily M, alpha member 1 | 62388890 |
| latent transforming growth factor beta binding protein 1 | 261337165 |
| latent transforming growth factor beta binding protein 2 | 4557733 |
| latent transforming growth factor beta binding protein 3 | 18497288 |
| latrophilin 1 isoform 2 precursor | 41281557 |
| latrophilin 2 precursor | 6912464 |
| lecithin-cholesterol acyltransferase precursor | 4557892 |
| lectin, mannose-binding, 1 precursor | 5031873 |
| leishmanolysin-like (metallopeptidase M8 family) | 110556638 |
| LEM domain containing 3 | 7706607 |
| leptin receptor isoform 2 | 51093379 |
| leucine rich repeat and fibronectin type III domain containing 3 | 13375646 |
| leucine rich repeat and fibronectin type III domain containing 5 | 31542244 |
| leucine rich repeat and sterile alpha motif containing 1 | 53729363 |
| leucine rich repeat containing 33 | 38348406 |
| leucine rich repeat neuronal 1 | 153791330 |
| leucine rich repeat neuronal 3 | 153792227 |
| leucine rich repeat neuronal 6C | 22749183 |
| leucine zipper-EF-hand containing transmembrane protein 1 | 6912482 |
| leucine-rich repeat LGI family, member 2 | 21313638 |
| leucine-rich repeat-containing G protein-coupled receptor 4 | 157694513 |
| leucine-rich repeat-containing G protein-coupled receptor 6 | 62912472 |
| leucine-rich repeats and immunoglobulin-like domains 2 | 7662320 |
| leucine-rich, glioma inactivated 1 precursor | 4826816 |
| leucyl/cystinyl aminopeptidase isoform 1 | 61742777 |
| leukemia inhibitory factor receptor precursor | 4504993 |
| leukocyte immunoglobulin-like receptor subfamily A member 4 | 47519953 |
| leukocyte immunoglobulin-like receptor subfamily A member 3 isoform 1 | 289547631 |
| leukocyte tyrosine kinase isoform 2 | 46249416 |
| leukocyte-derived arginine aminopeptidase | 11641261 |
| limbic system-associated membrane protein | 45594240 |
| hepatic triacylglycerol lipase precursor | 194097335 |
| lipoprotein lipase precursor | 4557727 |
| lipoprotein, Lp(a) | 116292750 |
| LISCH protein isoform 3 | 45593136 |
| LMBR1 domain containing 2 | 56790897 |
| low density lipoprotein receptor precursor | 4504975 |
| low density lipoprotein receptor-related protein 11 | 239787919 |
| low density lipoprotein receptor-related protein 5 | 119709832 |
| low density lipoprotein receptor-related protein 8 isoform 3 p | 61744467 |
| low density lipoprotein receptor-related protein associated pr | 4505021 |
| low density lipoprotein-related protein 1 | 126012562 |
| low density lipoprotein-related protein 1B | 93102379 |
| low density lipoprotein-related protein 2 | 126012573 |
| LPLUNC1 protein precursor | 40807482 |
| lymphocyte antigen 6 complex G5B | 50845399 |
| lymphocyte antigen 75 | 144446030 |
| lymphocyte-activation protein 3 precursor | 167614500 |
| lymphocyte-specific protein tyrosine kinase | 112789546 |
| LYRIC/3D3 | 223555917 |
| LysM, putative peptidoglycan-binding, domain containing 3 | 84370276 |
| lysophosphatidylglycerol acyltransferase 1 | 7661996 |
| lysosomal acid phosphatase 2 precursor | 4557010 |
| lysosomal-associated membrane protein 1 | 112380628 |
| lysosomal-associated membrane protein 2 precursor | 7669503 |
| lysozyme precursor | 4557894 |
| lysozyme-like 4 | 21389465 |
| major histocompatibility complex, class I, A precursor | 24797067 |
| major histocompatibility complex, class I, B | 17986001 |
| major histocompatibility complex, class I, C precursor | 52630342 |
| major histocompatibility complex, class I, F precursor | 149158698 |
| maltase-glucoamylase | 221316699 |
| mannan-binding lectin serine protease 1 isoform 1 precursor | 21264357 |
| mannose receptor, C type 1-like 1 | 57546917 |
| C-type mannose receptor 2 | 110624774 |
| mannosyl (alpha-1,3-)-glycoprotein beta-1,2-N-acetylglucosaminyltransferase | 167857778 |
| mannosyl (alpha-1,6-)-glycoprotein beta-1,2-N-acetylglucosaminyltransferase | 4505163 |
| mannosyl (beta-1,4-)-glycoprotein beta-1,4-N-acetylglucosaminyltransferase | 148539888 |
| MAS-related GPR, member X2 | 16876451 |
| mast cell carboxypeptidase A3 precursor | 221316749 |
| matrilin 4 isoform 3 precursor | 13699836 |
| matrix metalloproteinase 10 preproprotein | 4505205 |
| matrix metalloproteinase 13 preproprotein | 4505209 |
| matrix metalloproteinase 14 preproprotein | 4826834 |
| matrix metalloproteinase 20 preproprotein | 45359865 |
| matrix metalloproteinase 27 | 73808268 |
| matrix metalloproteinase 3 preproprotein | 4505217 |
| matrix metalloproteinase 7 preproprotein | 4505219 |
| matrix, extracellular phosphoglycoprotein with ASARM motif | 9910430 |
| McLeod syndrome-associated, Kell blood group protein | 10835267 |
| megalencephalic leukoencephalopathy with subcortical cysts 1 g | 14589896 |
| melanoma cell adhesion molecule | 71274107 |
| melanotransferrin isoform 1 precursor | 134244281 |
| melanoma-associated chondroitin sulfate proteoglycan 4 | 47419930 |
| membrane associated guanylate kinase, WW and PDZ domain containing 1; CNKSR family member 3 | 74272282 |
| membrane component chromosome 11 surface marker 1 isoform 2 | 42734503 |
| membrane metallo-endopeptidase-like 1 | 239049391 |
| membrane protein, palmitoylated 5 | 38570142 |
| membrane protein, palmitoylated 6 | 21361598 |
| met proto-oncogene precursor | 42741655 |
| metaxin 1 isoform 1 | 38569475 |
| microfibrillar-associated protein 1 | 50726968 |
| microfibrillar-associated protein 3-like isoform 1 | 153792329 |
| mitochondrial import receptor Tom22 | 9910382 |
| monoamine oxidase A | 4557735 |
| monooxygenase, DBH-like 1 isoform 1 | 118421087 |
| motile sperm domain containing 2 | 22749197 |
| mucin 16 | 83367077 |
| mucin 17 | 91982772 |
| mucolipin 1 | 10092597 |
| mucolipin 2 | 54792073 |
| multiple C2-domains with two transmembrane regions 1 isoform S | 50582996 |
| myelin protein zero-like 1 isoform a | 4506357 |
| myocilin | 4557779 |
| N system amino acid transporter NAT-1 | 289577102 |
| Na+/K+ -ATPase alpha 1 subunit isoform a proprotein | 21361181 |
| Na+/K+ -ATPase alpha 2 subunit proprotein | 4502271 |
| Na+/K+ -ATPase alpha 4 subunit isoform 1 | 153946397 |
| Na+/K+ -ATPase beta 3 subunit | 4502281 |
| N-acetylated alpha-linked acidic dipeptidase 2 | 126157508 |
| N-acetylglucosamine-1-phosphate transferase | 38202211 |
| N-acetylglucosamine-1-phosphodiester alpha-N-acetylglucosamini | 66346700 |
| NADH dehydrogenase (ubiquinone) 1 beta subcomplex, 4, 15kDa | 6041669 |
| NADH dehydrogenase (ubiquinone) 1 beta subcomplex, 8, 19kDa | 4826854 |
| NADH dehydrogenase (ubiquinone) 1 beta subcomplex, 9, 22kDa | 6274550 |
| NADH dehydrogenase (ubiquinone) 1, subcomplex unknown, 1, 6kDa | 4505367 |
| NADPH oxidase, EF hand calcium-binding domain 5 | 20127624 |
| natriuretic peptide precursor C | 13249346 |
| natriuretic peptide receptor A/guanylate cyclase A | 167830411 |
| natriuretic peptide receptor B precursor | 4580422 |
| N-deacetylase/N-sulfotransferase (heparan glucosaminyl) 2 | 4505353 |
| N-deacetylase/N-sulfotransferase (heparan glucosaminyl) 4 | 12007650 |
| nectin 3 | 11386199 |
| Nedd4 family interacting protein 1 | 13386480 |
| neogenin homolog 1 | 157311649 |
| nephrin | 4758822 |
| nephronectin | 75709198 |
| nerve growth factor receptor precursor | 4505393 |
| nerve growth factor, beta polypeptide precursor | 70995319 |
| nesprin 1 isoform beta | 154277118 |
| nesprin 1 isoform longer | 23097308 |
| netrin 4 | 93204871 |
| netrin-G1 ligand | 51317373 |
| neural cell adhesion molecule 1 isoform 1 | 94420689 |
| neural stem cell-derived dendrite regulator | 27262632 |
| pro-neuregulin-1, membrane-bound isoform isoform HRG-beta1 | 116006955 |
| neuregulin 1 isoform HRG-gamma | 116006965 |
| neuregulin 2 isoform 2 | 7669528 |
| neurexin 1 isoform alpha precursor | 14149613 |
| neurexin 2 isoform alpha-1 precursor | 14211536 |
| neurexophilin 3 | 217330576 |
| neurofascin precursor | 89903008 |
| neuromedin B isoform 1 | 45505143 |
| neuronal pentraxin II | 28195384 |
| neuropilin 1 isoform a | 182508169 |
| neuroplastin isoform a precursor | 9257240 |
| choline transporter-like protein 4 isoform 1 | 148612887 |
| NG37 protein | 153945852 |
| NHL repeat containing 2 | 42476013 |
| nicastrin precursor | 24638433 |
| nicotinamide nucleotide transhydrogenase | 122939153 |
| nidogen-1 precursor | 115298674 |
| Niemann-Pick disease, type C1 | 255652944 |
| nitric oxide synthase 1 (neuronal) | 10835173 |
| N-methyl-D-aspartate receptor subunit 2B precursor | 167003331 |
| nodal modulator 3 | 51944969 |
| notch 2 preproprotein | 24041035 |
| Notch homolog 3 | 134244285 |
| notch4 preproprotein | 55770876 |
| Numb-interacting protein | 46485465 |
| nurim | 25282391 |
| occludin | 4505487 |
| odorant binding protein 2A | 7657405 |
| odorant binding protein 2A | 7657405 |
| olfactomedin-like 2A | 116014339 |
| olfactomedin-like 3 | 9910270 |
| olfactory receptor, family 1, subfamily M, member 1 | 52218828 |
| olfactory receptor, family 10, subfamily X, member 1 | 52317184 |
| olfactory receptor, family 2, subfamily B, member 11 | 52317192 |
| olfactory receptor, family 2, subfamily T, member 35 | 49226830 |
| olfactory receptor, family 4, subfamily C, member 11 | 52317232 |
| olfactory receptor, family 4, subfamily D, member 1 | 50897264 |
| olfactory receptor, family 5, subfamily A, member 2 | 50080195 |
| olfactory receptor, family 5, subfamily AT, member 1 | 50233854 |
| olfactory receptor, family 51, subfamily G, member 2 | 52546689 |
| olfactory receptor, family 51, subfamily Q, member 1 | 52353941 |
| olfactory receptor, family 52, subfamily E, member 8 | 52353326 |
| olfactory receptor, family 52, subfamily N, member 4 | 221219017 |
| olfactory receptor, family 56, subfamily B, member 1 | 52353340 |
| olfactory receptor, family 6, subfamily C, member 4 | 53828708 |
| olfactory receptor, family 6, subfamily K, member 2 | 52627155 |
| olfactory receptor, family 6, subfamily M, member 1 | 52693923 |
| olfactory receptor, family 7, subfamily C, member 2 | 13624325 |
| olfactory receptor, family 8, subfamily J, member 1 | 52353298 |
| olfactory receptor, family 9, subfamily Q, member 1 | 52627203 |
| opsin 3 isoform 1 | 71999131 |
| opsin 4 isoform 1 | 15150803 |
| orexin receptor 2 | 222080109 |
| solute carrier family 22 member 14 | 157426885 |
| ORM1-like 3 | 27544927 |
| osteoblast differentiation promoting factor protein | 21361649 |
| otoancorin isoform 1 | 77404409 |
| otoferlin isoform a | 34740331 |
| ovochymase 1 | 110815798 |
| oxysterol binding protein 2 isoform a | 13540513 |
| oxysterol-binding protein-like 1A isoform B | 19718741 |
| P protein | 157266326 |
| protein CIP2A | 190194355 |
| pad-1-like | 45827701 |
| MAGUK p55 subfamily member 7 | 111154074 |
| pancreatic carboxypeptidase B1 preproprotein | 54607080 |
| pannexin 1 | 39995064 |
| pannexin 2 | 163659920 |
| papilin | 145309328 |
| paralemmin 2 isoform a | 290491128 |
| paraoxonase 3 | 29788996 |
| paraplegin isoform 1 | 4507173 |
| protein patched homolog 1 isoform L | 134254446 |
| patched domain containing 1 | 148271104 |
| PDZ domain containing 2 | 87196343 |
| pecanex homolog | 126723564 |
| penumbra | 30425534 |
| peropsin | 5730019 |
| peroxisomal biogenesis factor 11A | 4505717 |
| peroxisomal biogenesis factor 12 | 4505721 |
| PERP, TP53 apoptosis effector | 31377722 |
| phosphatidate cytidylyltransferase 1 | 22035624 |
| phosphatidylinositol glycan, class L | 4758922 |
| phosphatidylinositol glycan, class T precursor | 23397653 |
| phospholemman precursor | 11612672 |
| phospholipase A2, group III precursor | 142976884 |
| phospholipase A2, group III precursor | 7657126 |
| phospholipase A2, group VI isoform b | 52486251 |
| phospholipase B1 | 283436112 |
| phospholipid scramblase 3 | 31543417 |
| phospholipid transfer protein isoform a precursor | 5453914 |
| phosphorylase b kinase regulatory subunit alpha, skeletal muscle isoform isoform 1 | 169881273 |
| phosphorylase kinase, alpha 2 (liver) | 4505781 |
| piggyBac transposable element derived 5 | 25777748 |
| placental alkaline phosphatase preproprotein | 94721246 |
| plasma membrane calcium ATPase 1 isoform 1b | 48255945 |
| plasma membrane calcium ATPase 2 isoform b | 48255949 |
| plasma membrane calcium ATPase 4 isoform 4b | 48255957 |
| plasmalemma vesicle associated protein | 13775238 |
| plasminogen activator, urokinase receptor isoform 2 precursor | 53829379 |
| plasticity related gene 1 | 33636722 |
| pleckstrin homology domain containing, family H | 55741447 |
| plexin A1 | 262118282 |
| plexin B1 | 40254442 |
| plexin B3 | 29336063 |
| plexin C1 | 5032223 |
| plexin D1 | 157694524 |
| podocalyxin-like precursor isoform 1 | 66277202 |
| podocin | 7657615 |
| poliovirus receptor | 209413726 |
| poliovirus receptor-related 1 (herpesvirus entry mediator C) | 42560237 |
| poliovirus receptor-related 1 (herpesvirus entry mediator C) | 42560231 |
| poliovirus receptor-related 2 (herpesvirus entry mediator B) | 5360210 |
| polycystic kidney disease 2-like 2 | 116812588 |
| polycystin 1 isoform 2 precursor | 205360962 |
| polycystin 2 | 4505835 |
| polypeptide N-acetylgalactosaminyltransferase 2 | 4758412 |
| polypeptide N-acetylgalactosaminyltransferase 3 | 153266878 |
| polyposis locus protein 1-like 1 | 19923919 |
| potassium channel regulator isoform 2 | 40807368 |
| potassium channel tetramerisation domain containing 12 | 19923973 |
| potassium channel, subfamily K, member 1 | 4504847 |
| potassium channel, subfamily K, member 10 isoform 1 | 10863961 |
| potassium channel, subfamily K, member 2 isoform b | 14589851 |
| potassium channel, subfamily K, member 3 | 4504849 |
| potassium channel, subfamily K, member 5 | 4504851 |
| potassium chloride cotransporter KCC3 | 4826780 |
| potassium family, subfamily K, member 15 | 11641275 |
| potassium intermediate/small conductance calcium-activated cha | 25777643 |
| potassium inwardly-rectifying channel J11 | 62388888 |
| potassium inwardly-rectifying channel J16 | 8923823 |
| potassium inwardly-rectifying channel J5 | 24797141 |
| potassium inwardly-rectifying channel J6 | 4504843 |
| potassium inwardly-rectifying channel J8 | 4826802 |
| potassium voltage-gated channel KQT-like protein 2 isoform a | 26051264 |
| potassium voltage-gated channel KQT-like protein 3 | 4758630 |
| potassium voltage-gated channel KQT-like protein 4 isoform a | 26638653 |
| potassium voltage-gated channel, Shab-related subfamily, member 2 | 27436974 |
| potassium voltage-gated channel, Shab-related subfamily, member 1 | 4826784 |
| potassium voltage-gated channel, shaker-related subfamily, beta member 2 | 4504825 |
| potassium voltage-gated channel, shaker-related subfamily, member 5 | 25952087 |
| potassium voltage-gated channel, Shal-related subfamily, member 1 | 27436981 |
| potassium voltage-gated channel, subfamily H, member 5 | 22024390 |
| potassium voltage-gated channel, subfamily H, member 7 | 27886653 |
| transmembrane protein C12orf51 | 292781435 |
| probable phospholipid-transporting ATPase VB | 149944474 |
| sushi domain-containing protein 5 precursor | 150378552 |
| ATP-grasp domain-containing protein 1 isoform 2 | 148839342 |
| hypothetical protein LOC653567 | 150378481 |
| paralemmin-3 | 222537747 |
| hypothetical protein LOC643155 | 115392144 |
| PREDICTED: leucine rich repeat containing 38 | 239747236 |
| macrophage expressed gene 1 precursor | 133505169 |
| mucin-6 | 151301154 |
| WD repeat-containing protein 87 | 221219020 |
| PREDICTED: otogelin | 239744300 |
| plexin-B2 precursor | 149363636 |
| transmembrane protein 131 | 150456424 |
| SCO-spondin precursor | 134031945 |
| thrombospondin type-1 domain-containing protein 7A precursor | 150170672 |
| collagen alpha-6(VI) chain precursor | 156616290 |
| PREDICTED: chromosome 10 open reading frame 112 | 239744011 |
| protein NYNRIN | 126723547 |
| transmembrane protein 181 | 153792042 |
| patched domain-containing protein 2 | 149274653 |
| transmembrane protein 150B precursor | 148232433 |
| protein GPR108 isoform 2 | 130489591 |
| immunoglobulin superfamily, member 9B | 148886752 |
| leucine-rich repeat and fibronectin type III domain-containing protein 1 precursor | 149773484 |
| E3 ubiquitin-protein ligase MARCH11 | 156523244 |
| olfactory receptor 56A5 | 225637544 |
| T cell-interacting, activating receptor on myeloid cells 1 | 208879429 |
| PREDICTED: similar to transmembrane protease, serine 9 | 169163549 |
| PREDICTED: similar to trophoblast glycoprotein | 113423021 |
| transmembrane channel-like protein 3 | 122937502 |
| pregnancy specific beta-1-glycoprotein 1 | 21361392 |
| pregnancy specific beta-1-glycoprotein 11 isoform 2 | 164663824 |
| pregnancy specific beta-1-glycoprotein 4 isoform 1 | 42560235 |
| pregnancy-associated plasma protein A preproprotein | 38045915 |
| pregnancy-induced growth inhibitor isoform 2 | 33695099 |
| pregnancy-zone protein | 162809334 |
| prenylcysteine oxidase 1 | 166795301 |
| progesterone membrane binding protein | 291621647 |
| progesterone receptor membrane component 1 | 5729875 |
| prohibitin | 4505773 |
| prolactin-induced protein | 4505821 |
| proline rich Gla (G-carboxyglutamic acid) 1 | 4506135 |
| proline rich Gla (G-carboxyglutamic acid) 4 | 13129074 |
| prolyl 4-hydroxylase, beta subunit | 20070125 |
| prominin 1 | 5174387 |
| pro-oncosis receptor inducing membrane injury precursor | 100913218 |
| proopiomelanocortin preproprotein | 80861463 |
| proprotein convertase subtilisin/kexin type 5 preproprotein | 20336246 |
| proprotein convertase subtilisin/kexin type 9 preproprotein | 31317307 |
| prostaglandin E receptor 3, subtype EP3 isoform 5 | 38505180 |
| prostaglandin F2 receptor negative regulator | 41152506 |
| prostaglandin I2 (prostacyclin) synthase | 13699859 |
| protease inhibitor 16 precursor | 70780384 |
| protease, serine, 2 preproprotein | 4506147 |
| PRotein Associated with Tlr4 | 22749479 |
| protein C (inactivator of coagulation factors Va and VIIIa) | 4506115 |
| protein disulfide isomerase-associated 2 | 94966757 |
| protein disulfide isomerase-associated 3 precursor | 21361657 |
| protein disulfide isomerase-associated 4 | 4758304 |
| protein disulfide isomerase-associated 5 | 5803121 |
| protein disulfide isomerase-associated 6 | 5031973 |
| protein tyrosine phosphatase, receptor type, B precursor | 157952213 |
| protein tyrosine phosphatase, receptor type, C isoform 3 precusor | 18641364 |
| protein tyrosine phosphatase, receptor type, D isoform 4 precusor | 283484022 |
| protein tyrosine phosphatase, receptor type, f polypeptide | 55769554 |
| protein tyrosine phosphatase, receptor type, G precursor | 194097398 |
| protein tyrosine phosphatase, receptor type, H precursor | 241896924 |
| protein tyrosine phosphatase, receptor type, J precursor | 148728162 |
| protein tyrosine phosphatase, receptor type, K precursor | 18860902 |
| protein tyrosine phosphatase, receptor type, N precursor | 4506321 |
| protein tyrosine phosphatase, receptor type, sigma isoform 4 p | 104487611 |
| protein tyrosine phosphatase, receptor type, T isoform 1 | 148539858 |
| protein tyrosine phosphatase-like | 38257153 |
| protocadherin 1 isoform 1 precursor | 27754771 |
| protocadherin 17 | 94538350 |
| protocadherin 21 precursor | 16933565 |
| protocadherin 7 isoform b precursor | 14589933 |
| protocadherin 8 isoform 1 precursor | 6631102 |
| protocadherin 9 isoform 2 precursor | 9966883 |
| protocadherin alpha 1 isoform 2 precursor | 14165400 |
| protocadherin alpha 2 isoform 2 precursor | 14165405 |
| protocadherin alpha 8 isoform 2 precursor | 14165422 |
| protocadherin beta 1 precursor | 14195607 |
| protocadherin beta 10 precursor | 9256602 |
| protocadherin beta 15 precursor | 9256610 |
| protocadherin beta 4 precursor | 9256616 |
| protocadherin beta 8 precursor | 11276081 |
| protocadherin gamma subfamily A, 1 isoform 1 precursor | 11056032 |
| protocadherin gamma subfamily A, 4 isoform 2 precursor | 14196468 |
| protocadherin gamma subfamily B, 3 isoform 2 precursor | 14270496 |
| protocadherin gamma subfamily C, 4 isoform 2 precursor | 14277682 |
| cadherin-related family member 2 | 285002216 |
| protogenin | 83281200 |
| proto-oncogene c-ros-1 protein precursor | 19924165 |
| PTK7 protein tyrosine kinase 7 isoform b precursor | 22902126 |
| PTK7 protein tyrosine kinase 7 isoform d precursor | 22902130 |
| PTPRF interacting protein alpha 2 | 29171755 |
| PTPRF interacting protein alpha 3 | 32189362 |
| purinergic receptor P2X1 | 4505545 |
| purinergic receptor P2X2 isoform B | 28416923 |
| collagen alpha-1(XXVI) chain | 19263347 |
| putative G protein coupled receptor | 6005772 |
| putative membrane protein | 24308133 |
| putative protein O-mannosyltransferase | 32455271 |
| quiescin Q6 isoform a | 13325075 |
| rabphilin 3A homolog | 45267837 |
| urea transporter 1 isoform 2 | 289802991 |
| RAS guanyl releasing protein 2 isoform 2 | 24797103 |
| ras homolog gene family, member U | 11034843 |
| RAS protein activator like 1 | 4759026 |
| Ras protein-specific guanine nucleotide-releasing factor 1 | 24797101 |
| Ras-related GTP binding D | 11034847 |
| receptor-type protein tyrosine phosphatase O isoform a precurs | 13677214 |
| recoverin | 4506459 |
| reelin isoform b | 27436940 |
| Reg receptor | 4503617 |
| regulator of G-protein signaling 10 isoform a | 52694755 |
| regulator of G-protein signalling 14 | 21361304 |
| regulator of G-protein signalling 20 isoform a | 41281805 |
| regulator of G-protein signalling 7 | 156627563 |
| relaxin 1 preproprotein | 5902052 |
| ret proto-oncogene isoform c | 10862701 |
| reticulon 1 isoform A | 10863935 |
| reticulon 1 isoform B | 45827778 |
| reticulon 3 isoform c | 41393604 |
| reticulon 4 isoform A | 24431935 |
| reticulon 4 isoform B | 24431933 |
| reticulon 4 receptor-like 1 | 30425553 |
| reticulon 4 receptor-like 2 | 30425563 |
| peripherin-2 | 118572596 |
| repulsive guidance molecule A isoform 3 | 261878452 |
| ammonium transporter Rh type A | 156627565 |
| Rho-associated, coiled-coil containing protein kinase 2 | 41872583 |
| rhodopsin kinase | 4506529 |
| rhomboid domain containing 1 | 33300639 |
| rhomboid, veinlet-like 6 isoform 1 | 93352556 |
| rhomboid-related protein 2 | 54144654 |
| ribophorin I precursor | 4506675 |
| ribophorin II precursor | 35493916 |
| ring finger protein 130 | 29788758 |
| ring finger protein 144 | 38045938 |
| ring finger protein 149 | 284447287 |
| ring finger protein 153 | 8923415 |
| ring finger protein 170 | 237858654 |
| roundabout homolog 4, magic roundabout | 17511435 |
| R-spondin1 | 84490388 |
| ryanodine receptor 1 (skeletal) | 113204615 |
| ryanodine receptor 2 | 112799847 |
| ryanodine receptor 3 | 126032338 |
| RYK receptor-like tyrosine kinase isoform 1 | 54607020 |
| S100 calcium binding protein A11 (calgizzarin) | 5032057 |
| S100 calcium-binding protein A10 | 4506761 |
| S100 calcium-binding protein A7 | 115298657 |
| S100 calcium-binding protein A8 | 21614544 |
| S100 calcium-binding protein A9 | 4506773 |
| Sad1 and UNC84 domain containing 1 | 71834868 |
| sarco/endoplasmic reticulum Ca2+ -ATPase isoform e | 28373105 |
| sarcolemma associated protein | 56550043 |
| sarcoma antigen NY-SAR-41 | 62243484 |
| scavenger receptor class A, member 3 isoform 2 | 33598922 |
| scavenger receptor class B, member 2 | 5031631 |
| secreted frizzled-related protein 5 | 188528609 |
| secreted modular calcium-binding protein 2 | 24308277 |
| secreted protein, acidic, cysteine-rich (osteonectin) | 4507171 |
| secretoglobin family 1D member 4 | 46240866 |
| secretogranin II precursor | 68160947 |
| seizure related 6 homolog | 148839280 |
| sel-1 suppressor of lin-12-like | 19923669 |
| selenoprotein N, 1 isoform 1 precursor | 47578099 |
| selenoprotein N, 1 isoform 1 precursor | 47578099 |
| sema domain, transmembrane domain (TM), and cytoplasmic domain | 11991660 |
| semaphorin 4C | 157388902 |
| semaphorin 4D | 214010218 |
| semaphorin 5A | 147904700 |
| semaphorin 5B isoform 1 | 91982767 |
| semaphorin 6B precursor | 19718780 |
| semaphorin 6C | 16306552 |
| semenogelin II precursor | 4506885 |
| serpin peptidase inhibitor, clade A (alpha-1 antiproteinase, antitrypsin), member 1 | 50363221 |
| serpin peptidase inhibitor, clade A (alpha-1 antiproteinase, antitrypsin), member 12 | 27777657 |
| serpin peptidase inhibitor, clade A (alpha-1 antiproteinase, antitrypsin), member 10 | 7705879 |
| serine (or cysteine) proteinase inhibitor, clade A, member 7 | 205277441 |
| serpin peptidase inhibitor, clade F (alpha-2 antiplasmin, pigment epithelium derived factor), member 1 | 39725934 |
| serpin peptidase inhibitor, clade H (heat shock protein 47), member 1, (collagen binding protein 1) | 32454741 |
| serine palmitoyltransferase, long chain base subunit 1 | 5454084 |
| serine peptidase inhibitor, Kazal type 2 (acrosin-trypsin inhibitor) | 10863911 |
| serine protease inhibitor, Kazal type 4 | 7657453 |
| serum amyloid P component precursor | 4502133 |
| shugoshin-like 1 isoform A1 | 60302875 |
| sialic acid binding Ig-like lectin 10 | 31377639 |
| sialic acid binding Ig-like lectin 6 isoform 2 precursor | 193794819 |
| sialic acid binding immunoglobulin-like lectin-like protein | 16506828 |
| sialophorin | 71979937 |
| sialyltransferase 1 isoform a | 4506949 |
| sialyltransferase 9 | 109633044 |
| SID1 transmembrane family, member 1 | 116812584 |
| sidekick homolog 1 | 119220552 |
| sideroflexin 2 | 30578418 |
| signal peptide peptidase-like 2B isoform 2 | 41281782 |
| single Ig IL-1R-related molecule | 205277445 |
| six transmembrane epithelial antigen of the prostate 2 | 100913194 |
| skeletal muscle receptor tyrosine kinase | 5031927 |
| SLIT and NTRK-like family, member 2 | 33504581 |
| slit and trk like 1 protein | 40217817 |
| slit and trk like 6 | 40217825 |
| slit homolog 2 | 4759146 |
| slit homolog 3 | 11321571 |
| slit-like 2 | 88702793 |
| small conductance calcium-activated potassium channel protein | 25777647 |
| small inducible cytokine B11 precursor | 4885589 |
| electrogenic sodium bicarbonate cotransporter 4 isoform a | 125987596 |
| sodium channel, nonvoltage-gated 1 alpha | 4506815 |
| sodium channel, nonvoltage-gated 1, gamma | 42476333 |
| sodium channel, voltage-gated, type IX, alpha | 4506813 |
| sodium channel, voltage-gated, type X, alpha | 110835710 |
| sodium channel, voltage-gated, type XI, alpha | 115583667 |
| sodium potassium chloride cotransporter 2 | 134254459 |
| soluble adenylyl cyclase | 209976994 |
| solute carrier family 1 (glial high affinity glutamate transporter), member 3 | 169790839 |
| solute carrier family 1 (high affinity aspartate/glutamate transporter), member 6 | 4827012 |
| solute carrier family 1 (neutral amino acid transporter), member 5 | 5032093 |
| solute carrier family 1 (neutral amino acid transporter), memb | 5032093 |
| solute carrier family 1 (glial high affinity glutamate transporter), member 2 | 40254478 |
| solute carrier family 1, member 4 | 21314632 |
| solute carrier family 10 (sodium/bile acid cotransporter family), member 4 | 24308414 |
| solute carrier family 12 (potassium/chloride transporters), member 9 | 31881740 |
| solute carrier family 12 (potassium/chloride transporters), me | 5730043 |
| solute carrier family 12 (sodium/chloride transporters), member 3 | 186910315 |
| solute carrier family 12 (sodium/potassium/chloride transporters), member 2 | 4506975 |
| solute carrier family 12 (potassium-chloride transporter), member 5 | 11968148 |
| solute carrier family 12 (potassium/chloride transporters), member 8 | 38569457 |
| solute carrier family 13 (sodium-dependent dicarboxylate transporter), member 2 | 4506979 |
| solute carrier family 14 (urea transporter), member 2 | 157694503 |
| solute carrier family 15 (H+/peptide transporter), member 2 | 226371746 |
| solute carrier family 15 (oligopeptide transporter), member 1 | 4827008 |
| monocarboxylate transporter 1 | 115583685 |
| solute carrier family 16, member 2 | 5730045 |
| solute carrier family 16, member 3 | 4759112 |
| solute carrier family 2 (facilitated glucose transporter), member 12 | 21553331 |
| solute carrier family 2 (facilitated glucose transporter), member 1 | 166795299 |
| solute carrier family 2 (facilitated glucose transporter), member 8 | 21361449 |
| solute carrier family 22 (organic cation transporter), member 18 antisense | 198041703 |
| solute carrier family 22 member 11 | 8923870 |
| solute carrier family 24 (sodium/potassium/calcium exchanger), member 2 | 9966787 |
| solute carrier family 25 (mitochondrial carrier; adenine nucleotide translocator), member 31 | 13775208 |
| solute carrier family 25 (mitochondrial carrier; adenine nucleotide translocator), member 4 | 55749577 |
| solute carrier family 25 (mitochondrial carrier; adenine nucle | 55749577 |
| solute carrier family 25 (mitochondrial carrier; oxoglutarate carrier), member 11 | 21361114 |
| solute carrier family 25 (mitochondrial carrier; phosphate carrier), member 23 | 48476342 |
| solute carrier family 25 member 24 isoform 1 | 148491091 |
| solute carrier family 25 member 3 isoform b precursor | 4505775 |
| solute carrier family 25, member 30 | 58197562 |
| solute carrier family 25, member 5 | 156071459 |
| solute carrier family 26, member 7 isoform b | 20336282 |
| solute carrier family 26, member 8 isoform a | 16418457 |
| solute carrier family 27 (fatty acid transporter), member 4 | 40807357 |
| solute carrier family 27 (fatty acid transporter), member 6 | 13325055 |
| solute carrier family 29 (nucleoside transporters), member 1 | 4826716 |
| solute carrier family 3 (activators of dibasic and neutral amino acid transport), member 2 | 61744481 |
| solute carrier family 30 (zinc transporter), member 9 | 57164948 |
| solute carrier family 32, member 1 | 17999520 |
| solute carrier family 34 (sodium phosphate), member 1 | 156627569 |
| solute carrier family 35 (UDP-glucuronic acid/UDP-N-acetylgala | 14028875 |
| solute carrier family 35, member B3 | 21361503 |
| solute carrier family 35, member E1 | 164607128 |
| solute carrier family 35, member F1 | 189027121 |
| solute carrier family 35, member F5 | 21361959 |
| solute carrier family 37 member 1 | 49619231 |
| solute carrier family 38, member 2 | 21361602 |
| solute carrier family 38, member 4 | 18482385 |
| solute carrier family 39 (zinc transporter), member 10 | 55741750 |
| zinc transporter ZIP6 isoform 1 | 153252201 |
| solute carrier family 39 (zinc transporter), member 7 | 117553608 |
| kanadaptin | 155722990 |
| solute carrier family 4, anion exchanger, member 2 | 156071474 |
| solute carrier family 4, anion exchanger, member 3 isoform 2 | 157671953 |
| sodium bicarbonate cotransporter 3 | 134288865 |
| solute carrier family 4, sodium bicarbonate transporter-like, | 155722998 |
| solute carrier family 41, member 2 | 207444687 |
| solute carrier family 5 (inositol transporters), member 3 | 110835708 |
| solute carrier family 5 (sodium/glucose cotransporter), member | 17941285 |
| solute carrier family 6, member 15 isoform 1 | 33354281 |
| solute carrier family 7 (cationic amino acid transporter, y+ s | 71979932 |
| cationic amino acid transporter 3 | 114326544 |
| solute carrier family 8 (sodium/calcium exchanger), member 1 | 10863913 |
| solute carrier family 9 (sodium/hydrogen exchanger), isoform 3 | 194239733 |
| solute carrier family 9 (sodium/hydrogen exchanger), isoform 5 | 4759144 |
| solute carrier family 9 (sodium/hydrogen exchanger), member 4 | 148727259 |
| solute carrier organic anion transporter family, member 1B1 | 225543525 |
| solute carrier organic anion transporter family, member 2B1 | 6005820 |
| solute carrier organic anion transporter family, member 3A1 | 222831575 |
| solute carrier organic anion transporter family, member 4C1 | 38679890 |
| solute carrier organic anion transporter family, member 6A1 | 93277099 |
| sortilin 1 preproprotein | 17149834 |
| sortilin-related receptor containing LDLR class A repeats | 4507157 |
| sorting and assembly machinery component 50 homolog | 225543166 |
| source of immunodominant MHC-associated peptides | 30578410 |
| sperm acrosome associated 3 | 27777653 |
| sperm associated antigen 9 isoform 1 | 27436920 |
| cation channel sperm-associated protein 1 | 221316609 |
| sperm-associated cation channel 2 isoform 2 | 26051223 |
| spermatogenesis associated 6 | 9506603 |
| spermatogenesis-associated protein 9 | 50659066 |
| sperm-specific sodium proton exchanger | 56786136 |
| SPFH domain family, member 2 isoform 1 | 6005721 |
| sphingomyelin phosphodiesterase 3, neutral membrane | 8923946 |
| sphingosine-1-phosphate lyase 1 | 31982936 |
| ST8 alpha-N-acetyl-neuraminide alpha-2,8-sialyltransferase 1 | 4506953 |
| stabilin 2 precursor | 61743980 |
| stanniocalcin 2 precursor | 4507267 |
| stearoyl-CoA desaturase | 53759151 |
| sterile alpha and TIR motif containing 1 | 154090976 |
| sterile alpha motif domain containing 1 | 39930517 |
| steroid dehydrogenase homolog | 7705855 |
| steroid-sensitive protein 1 | 41152074 |
| sterol O-acyltransferase 1 | 49533617 |
| stratum corneum chymotryptic enzyme preproprotein | 4826950 |
| sucrase-isomaltase (alpha-glucosidase) | 157364974 |
| sulfatase 2 isoform b precursor | 240255478 |
| suppressin | 38016945 |
| sushi domain containing 2 | 10092665 |
| SWAP-70 protein | 93102364 |
| synaptic vesicle protein 2C | 73695465 |
| synaptogyrin 1 isoform 1a | 22035696 |
| synaptogyrin 2 | 4759202 |
| synaptotagmin I | 5032139 |
| synaptotagmin II | 31543670 |
| synaptotagmin V | 92859638 |
| synaptotagmin VII | 38570146 |
| synaptotagmin-like 2 isoform b | 15011900 |
| synaptotagmin-like 4 (granuphilin-a) | 193804860 |
| syndecan 1 precursor | 55749480 |
| syndecan 2 precursor | 40548378 |
| syndecan 4 precursor | 38201675 |
| syntaxin 10 | 4507285 |
| syntaxin 2 isoform 1 | 37577287 |
| syntaxin 5 | 94400932 |
| syntaxin 8 | 4759188 |
| TAO kinase 2 isoform 2 | 45505130 |
| taste receptor, type 2, member 48 | 28882035 |
| T-cell lymphoma invasion and metastasis 1 | 115583670 |
| tectorin alpha precursor | 134268640 |
| tenascin precursor | 153946395 |
| tenascin N | 62988324 |
| tenascin XB isoform 1 | 188528648 |
| tensin | 66529407 |
| testis enhanced gene transcript (BAX inhibitor 1) | 148746209 |
| testis expressed sequence 10 | 8923269 |
| testis expressed sequence 2 | 38679909 |
| tetracycline transporter-like protein | 225703102 |
| thioredoxin-related transmembrane protein 1 precursor | 151101292 |
| thioredoxin domain containing 13 | 40254947 |
| thrombomodulin precursor | 4507483 |
| thrombospondin 3 precursor | 6005902 |
| thrombospondin type I domain-containing 1 isoform 1 | 8923894 |
| thrombospondin, type I, domain containing 6 | 226442878 |
| Thy-1 cell surface antigen | 19923362 |
| thymopoietin isoform alpha | 4507555 |
| thymopoietin isoform beta | 73760405 |
| thyroglobulin | 55770862 |
| thyrotropin-releasing hormone degrading enzyme | 7019561 |
| tight junction protein 2 (zona occludens 2) isoform 1 | 42518070 |
| tissue non-specific alkaline phosphatase precursor | 116734717 |
| TLC domain containing 1 | 19923999 |
| toll-like receptor 10 precursor | 62865618 |
| toll-like receptor 5 | 16751843 |
| toll-like receptor 7 | 7706093 |
| tolloid-like 1 | 22547221 |
| transcobalamin II precursor | 21071010 |
| transferrin | 4557871 |
| transferrin receptor | 189458817 |
| transforming growth factor, beta 3 | 4507465 |
| transforming growth factor, beta receptor II isoform B precurs | 67782324 |
| transforming growth factor, beta receptor III | 56682966 |
| trans-golgi network protein 2 | 42518068 |
| transient receptor potential 4 | 7706747 |
| transient receptor potential cation channel, subfamily C, member 5 | 6912736 |
| transient receptor potential cation channel, subfamily M, member 3 | 154091320 |
| transient receptor potential cation channel, subfamily M, member 2 | 4507689 |
| transient receptor potential cation channel, subfamily M, member 7 | 148612863 |
| transient receptor potential cation channel, subfamily V, member 1 | 74315352 |
| translocase of outer mitochondrial membrane 40 homolog (yeast)-like | 21362030 |
| translocase of outer mitochondrial membrane 70 homolog A (S. cerevisiae) | 54607135 |
| translocation associated membrane protein 1 | 7657655 |
| translocation protein 1 | 4507525 |
| transmembrane 4 superfamily member 6 | 4507541 |
| transmembrane 4 superfamily member 8 isoform 1 | 5032201 |
| transmembrane 9 superfamily member 1 isoform a | 21361315 |
| transmembrane 9 superfamily protein member 4 | 164519076 |
| transmembrane anchor protein 1 isoform 1 | 56549131 |
| transmembrane and coiled-coil domains 4 | 194239659 |
| transmembrane emp24 protein transport domain containing 9 | 39725636 |
| transmembrane protease, serine 11A | 167466250 |
| transmembrane protease, serine 4 isoform 1 | 15451940 |
| transmembrane protein 109 | 13129092 |
| transmembrane protein 132A isoform a | 30089935 |
| transmembrane protein 132B | 89111953 |
| transmembrane protein 132E | 46560555 |
| transmembrane protein 146 | 91598777 |
| anoctamin-2 | 209862785 |
| transmembrane protein 16G isoform NGEP long | 145207958 |
| transmembrane protein 2 | 7019555 |
| transmembrane protein 25 | 31377650 |
| transmembrane protein 30A | 8922720 |
| transmembrane protein 39B | 169234667 |
| transmembrane protein 4 | 7657176 |
| transmembrane protein 53 | 42734434 |
| transmembrane protein 57 | 31542661 |
| transmembrane protein 63B | 55769589 |
| Meckelin isoform 1 | 187281580 |
| transmembrane protein 7 | 13899263 |
| transmembrane protein 8 (five membrane-spanning domains) | 157676334 |
| transmembrane protein SHREW1 | 9055278 |
| triadin | 104526627 |
| CMRF35-like molecule 7 | 154759271 |
| tryptophan rich basic protein | 21536428 |
| tumor endothelial marker 8 isoform 1 precursor | 14149904 |
| tumor necrosis factor alpha | 25952111 |
| tumor necrosis factor receptor superfamily, member 10b | 224494019 |
| tumor necrosis factor receptor superfamily, member 11a | 4507565 |
| tumor necrosis factor receptor superfamily, member 18 | 23238194 |
| tumor necrosis factor receptor superfamily, member 19-like | 21361873 |
| tumor necrosis factor receptor superfamily, member 25 | 23200025 |
| tumor necrosis factor receptor superfamily, member 6 isoform 2 | 23510421 |
| tumor necrosis factor receptor superfamily, member 8 isoform 1 | 68348711 |
| tumor rejection antigen (gp96) 1 | 4507677 |
| epithelial cell adhesion molecule precursor | 218505670 |
| tumor-associated calcium signal transducer 2 precursor | 166795236 |
| tweety 1 isoform 1 | 10257437 |
| type 1 tumor necrosis factor receptor shedding aminopeptidase | 94818891 |
| type I hair keratin 1 | 14917115 |
| type II transmembrane protein DCAL1 | 25188187 |
| type IV alpha 6 collagen isoform A precursor | 148536823 |
| TYRO3 protein tyrosine kinase | 27597078 |
| ubiquitin protein ligase E3 component n-recognin 1 | 28372497 |
| ubiquitin protein ligase E3A isoform 2 | 19718766 |
| ubiquitin specific protease 9, X-linked isoform 4 | 145309311 |
| ubiquitin specific protease 9, Y-linked | 74319833 |
| ubiquitin-activating enzyme E1 | 23510338 |
| UDP glycosyltransferase 1 family, polypeptide A6 | 45827765 |
| UDP glycosyltransferase 2 family, polypeptide B11 | 4507823 |
| UDP glycosyltransferase 2 family, polypeptide B17 | 4507821 |
| UDP glycosyltransferase 2 family, polypeptide B7 | 190194389 |
| UDP-GalNAc:betaGlcNAc beta 1,3-galactosaminyltransferase, poly | 22749021 |
| UDP-GalNAc:polypeptide N-acetylgalactosaminyltransferase-like | 22538495 |
| UDP-GlcNAc:betaGal beta-1,3-N-acetylglucosaminyltransferase 2 | 9845238 |
| UDP-GlcNAc:betaGal beta-1,3-N-acetylglucosaminyltransferase 4 | 13540527 |
| UDP-GlcNAc:betaGal beta-1,3-N-acetylglucosaminyltransferase 6 | 118918426 |
| UDP-GlcNAc:betaGal beta-1,3-N-acetylglucosaminyltransferase-li | 57770468 |
| UDP-N-acetyl-alpha-D-galactosamine:polypeptide N-acetylgalactosaminyltransferase-like 2 | 190014583 |
| UDP-N-acetyl-alpha-D-galactosamine:polypeptide N-acetylgalactosaminyltransferase 5 (GalNAc-T5) | 32698686 |
| UDP-N-acetyl-alpha-D-galactosamine:polypeptide N-acetylgalactosaminyltransferase-like 5 | 281485547 |
| UDP-N-acetyl-alpha-D-galactosamine:polypeptide N-acetylgalactosaminyltransferase-like 1 | 270265820 |
| alpha-1,3-mannosyl-glycoprotein 4-beta-N-acetylglucosaminyltransferase C | 166197698 |
| unc-5 homolog B | 32261318 |
| serine/threonine-protein kinase ULK2 | 217330557 |
| unc5C | 16933525 |
| unc-84 homolog A | 110227866 |
| upregulated during skeletal muscle growth 5 | 14249376 |
| urocanase domain containing 1 | 21389467 |
| uroplakin 3B isoform b | 33149306 |
| uveal autoantigen with coiled-coil domains and ankyrin repeats | 59850762 |
| vang-like 1 | 20373171 |
| vascular cell adhesion molecule 1 isoform b precursor | 18201909 |
| v-erb-a erythroblastic leukemia viral oncogene homolog 4 | 4885215 |
| G-protein coupled receptor 98 precursor | 113722120 |
| vesicle-associated membrane protein 3 (cellubrevin) | 4759300 |
| vesicle-associated membrane protein 8 | 14043026 |
| vesicle-associated membrane protein-associated protein A | 94721250 |
| vitamin D-binding protein precursor | 32483410 |
| vitelliform macular dystrophy 2-like 2 | 23397576 |
| voltage gated channel like 1 | 24119274 |
| voltage-dependent calcium channel alpha 1G subunit isoform 7 | 38505274 |
| voltage-dependent T-type calcium channel alpha-1I subunit | 51093859 |
| voltage-gated potassium channel, subfamily H, member 2 | 26051273 |
| von Willebrand factor A domain containing 2 | 38348304 |
| von Willebrand factor preproprotein | 89191868 |
| V-set and immunoglobulin domain containing 4 | 6005958 |
| V-set domain containing T cell activation inhibitor 1 | 99028881 |
| wingless-type MMTV integration site family, member 2B | 13518017 |
| wingless-type MMTV integration site family, member 8A | 17505195 |
| wingless-type MMTV integration site family, member 9B | 13518017 |
| X Kell blood group precursor-related family, member 4 | 59709427 |
| xenotropic and polytropic retrovirus receptor | 19923272 |
| X-linked neuroligin 4 | 31317256 |
| X-linked phosphate regulating endopeptidase homolog | 90403592 |
| xylosyltransferase I | 28269693 |
| Yip1 interacting factor homolog B isoform 5 | 89191848 |
| zinc finger protein 261 | 4827067 |
| zinc finger protein-like 1 | 33300635 |
| zinc finger, DHHC domain containing 11 | 13376150 |
| zinc finger, DHHC domain containing 6 | 11968053 |
| zinc finger, DHHC domain containing 8 | 32698692 |
| zinc finger, DHHC domain containing 9 | 56682972 |
| zona pellucida glycoprotein 2 preproprotein | 4508045 |
| zona pellucida glycoprotein 3 preproprotein | 38327649 |
| zonadhesin isoform 6 | 27881494 |
