# Supplementary material for: Proteomic Analyses Reveal Common Promiscuous Patterns of Cell Surface Proteins on Human Embryonic Stem Cells and Sperms
Source: PLoS One. 2011 May 3;6(5):e19386. doi: 10.1371/journal.pone.0019386 (PMC3086920; doi:10.1371/journal.pone.0019386)
Supplement: Table S2 — A list of cell surface proteins on hSperm cells identified in this study. (DOC) [file pone.0019386.s002.doc]

Table S2: hSperm cell surface proteins

| **Protein Name** | **NCBI**  **Protein GI** |
| --- | --- |
| 1-acylglycerol-3-phosphate O-acyltransferase 1 | 15100175 |
| 1-acylglycerol-3-phosphate O-acyltransferase 3 | 83267874 |
| 1-acylglycerol-3-phosphate O-acyltransferase 4 isoform a | 9910392 |
| 1-acylglycerol-3-phosphate O-acyltransferase 5 | 61743952 |
| 3-hydroxy-3-methylglutaryl-Coenzyme A reductase | 4557643 |
| 3-hydroxy-3-methylglutaryl-Coenzyme A synthase 1 (soluble) | 148298764 |
| 3-hydroxyisobutyryl-Coenzyme A hydrolase isoform 1 | 37594471 |
| 5-hydroxytryptamine (serotonin) receptor 2B | 222080049 |
| A kinase (PRKA) anchor protein 8-like | 49472841 |
| AAA-ATPase TOB3 | 75677353 |
| aarF domain containing kinase 1 | 40254938 |
| aarF domain containing kinase 2 | 32261307 |
| aarF domain containing kinase 5 | 41393593 |
| absent in melanoma 2 | 4757734 |
| acetyl-Coenzyme A acyltransferase 1 | 4501853 |
| acetyl-Coenzyme A carboxylase alpha isoform 1 | 38679960 |
| activin beta E | 13899338 |
| acyl-Coenzyme A binding domain containing 5 | 203098629 |
| ADAM metallopeptidase domain 12 isoform 1 preproprotein | 73747885 |
| ADAM metallopeptidase domain 28 isoform 1 preproprotein | 98985828 |
| ADAM metallopeptidase domain 30 preproprotein | 31881770 |
| ADAM metallopeptidase with thrombospondin type 1 motif, 10 | 56121815 |
| ADAM metallopeptidase with thrombospondin type 1 motif, 17 | 110611170 |
| ADAM metallopeptidase with thrombospondin type 1 motif, 2 | 11038659 |
| ADAM metallopeptidase with thrombospondin type 1 motif, 5 | 195539372 |
| ADAM metallopeptidase with thrombospondin type 1 motif, 6 | 64276808 |
| ADAM metallopeptidase with thrombospondin type 1 motif, 7 | 38683827 |
| ADAM metallopeptidase with thrombospondin type 1 motif, 9 | 33624896 |
| ADAM-like, decysin 1 | 7657319 |
| adenosine monophosphate deaminase 2 (isoform L) isoform 1 | 21264318 |
| adenylate cyclase 2 | 115387102 |
| adenylate cyclase 9 | 50959205 |
| matrix-remodeling-associated protein 5 precursor | 139948432 |
| ADP-ribosyltransferase 3 | 21361167 |
| afamin precursor | 4501987 |
| agrin | 54873613 |
| albumin precursor | 4502027 |
| aldehyde dehydrogenase 16 family, member A1 | 223972651 |
| aldehyde dehydrogenase 3A2 isoform 1 | 73466520 |
| alpha 1 type I collagen preproprotein | 110349772 |
| alpha 1 type II collagen isoform 2, preproprotein | 111118974 |
| alpha 1 type IV collagen preproprotein | 148536825 |
| alpha 1 type VII collagen precursor | 4502961 |
| alpha 1 type XI collagen isoform A preproprotein | 98985806 |
| alpha 1 type XIX collagen precursor | 47778921 |
| alpha 2 globin | 4504345 |
| alpha 2 type IX collagen | 11386161 |
| alpha 2 type V collagen preproprotein | 89363017 |
| alpha 2 type VI collagen isoform 2C2a precursor | 115527070 |
| alpha 3 type IV collagen isoform 2 precursor | 89142733 |
| alpha 3 type IX collagen | 119508426 |
| alpha 3 type VI collagen isoform 2 precursor | 55743100 |
| alpha 4 type IV collagen precursor | 116256356 |
| alpha glucosidase II alpha subunit isoform 3 | 88900491 |
| alpha-1D-adrenergic receptor | 4501957 |
| alpha-2A-adrenergic receptor | 194353970 |
| alpha-2-glycoprotein 1, zinc | 4502337 |
| WD repeat-containing protein 81 isoform 2 | 116256466 |
| amnionless protein | 110611172 |
| amphoterin induced gene 2 | 32469517 |
| anaplastic lymphoma kinase Ki-1 | 29029632 |
| angiomotin | 19111150 |
| angiopoietin 2 | 4557315 |
| angiopoietin 4 | 7705276 |
| angiopoietin-like 2 precursor | 6912236 |
| angiopoietin-like 5 | 187960047 |
| angiotensin I converting enzyme 2 precursor | 11225609 |
| angiotensin I converting enzyme isoform 1 precursor | 4503273 |
| angiotensin II receptor, type 1 | 6715583 |
| ankylosis, progressive homolog | 16905507 |
| annexin A13 isoform a | 51895795 |
| annexin A2 isoform 2 | 4757756 |
| apolipoprotein A-I preproprotein | 4557321 |
| apolipoprotein A-IV precursor | 71773110 |
| apolipoprotein B precursor | 105990532 |
| apolipoprotein C-III precursor | 4557323 |
| apolipoprotein C-IV | 4502161 |
| apolipoprotein E precursor | 4557325 |
| apolipoprotein L domain containing 1 | 194353993 |
| aquaporin 6 | 86792455 |
| archaemetzincins-2 isoform 1 | 75812966 |
| ARG99 protein | 83415184 |
| arginyl aminopeptidase (aminopeptidase B) | 40316915 |
| asialoglycoprotein receptor 1 | 4502251 |
| astrotactin 2 isoform a | 46488915 |
| atlastin isoform b | 74024917 |
| ATP binding cassette, sub-family A (ABC1), member 13 | 31657092 |
| ATP synthase mitochondrial F1 complex assembly factor 1 | 110349780 |
| ATP synthase, H+ transporting, mitochondrial F0 complex, subunit C1 (subunit 9) | 50659069 |
| ATP synthase, H+ transporting, mitochondrial F1 complex, beta polypeptide | 32189394 |
| ATP synthase, H+ transporting, mitochondrial F1 complex, gamma polypeptide 1 | 50345988 |
| ATPase type 13A4 | 66932949 |
| ATPase type 13A5 | 66730421 |
| ATPase, Ca++ transporting, cardiac muscle, slow twitch 2 | 4502285 |
| ATPase, Class I, type 8B, member 1 | 5031697 |
| ATPase, Class I, type 8B, member 3 | 44888835 |
| ATPase, Class II, type 9B | 41327760 |
| ATPase, Class V, type 10D | 222352161 |
| ATPase, Class VI, type 11C isoform a | 40316839 |
| ATPase, Cu++ transporting, alpha polypeptide | 115529486 |
| ATPase, H+ transporting, lysosomal 42kDa, V1 subunit C1 | 4502315 |
| ATPase, H+ transporting, lysosomal 50/57kDa, V1 subunit H | 47717100 |
| ATPase, H+ transporting, lysosomal 56/58kDa, V1 subunit B1 | 19913426 |
| ATP-binding cassette sub-family B member 1 | 42741659 |
| ATP-binding cassette sub-family G member 1 isoform 5 | 46592964 |
| ATP-binding cassette, sub-family A , member 5 | 27262624 |
| ATP-binding cassette, sub-family A member 1 | 21536376 |
| ATP-binding cassette, sub-family A, member 10 | 153792144 |
| ATP-binding cassette, sub-family A, member 2 isoform a | 45446740 |
| ATP-binding cassette, sub-family A, member 9 | 27477115 |
| ATP-binding cassette, sub-family B (MDR/TAP), member 11 | 21536378 |
| ATP-binding cassette, sub-family B, member 6 | 9955963 |
| ATP-binding cassette, sub-family B, member 7 | 42490749 |
| ATP-binding cassette, sub-family C, member 5 isoform 1 | 66529005 |
| ATP-binding cassette, sub-family C, member 8 | 118582255 |
| ATP-binding cassette, sub-family C, member 9 isoform SUR2B | 110832837 |
| ATP-binding cassette, sub-family D, member 2 | 9945308 |
| BCL2-associated X protein isoform beta | 4757838 |
| BCL2-like 1 isoform 1 | 20336335 |
| BCL2-like 13 (apoptosis facilitator) | 45243501 |
| beta 1,4-N-acetylgalactosaminyltransferase-transferase-III | 71043500 |
| beta 3-glycosyltransferase-like | 154689817 |
| beta-1,3-galactosyl-O-glycosyl-glycoprotein beta-1,6-N-acetylg | 21614523 |
| beta-1,3-glucuronyltransferase 3 | 12408654 |
| beta-1,4-N-acetyl-galactosaminyl transferase 1 | 4503893 |
| beta-microseminoprotein isoform a precursor | 4557036 |
| biglycan preproprotein | 4502403 |
| blocked early in transport 1 | 5031611 |
| BMP and activin membrane-bound inhibitor precursor | 6912534 |
| bone marrow stromal cell antigen 1 precursor | 168229159 |
| bone morphogenetic protein 3 (osteogenic) precursor | 126507087 |
| bone morphogenetic protein receptor, type IA precursor | 41349437 |
| brain adenylate cyclase 1 | 31083193 |
| brain-specific angiogenesis inhibitor 2 | 115387099 |
| bromodomain and WD repeat domain containing 2 | 13324688 |
| butyrophilin, subfamily 1, member A1 | 166197658 |
| butyrophilin, subfamily 3, member A3 isoform a | 5901908 |
| butyrylcholinesterase precursor | 4557351 |
| C1q and tumor necrosis factor related protein 4 | 13994273 |
| C3 and PZP-like, alpha-2-macroglobulin domain containing 8 | 118600977 |
| Ca2+-dependent activator protein for secretion 2 isoform a | 148839294 |
| cadherin 11, type 2 preproprotein | 16306532 |
| cadherin 16 precursor | 4757956 |
| cadherin 3, type 1 preproprotein | 14589891 |
| cadherin 4, type 1 preproprotein | 14589893 |
| cadherin 9, type 2 preproprotein | 90193626 |
| cadherin EGF LAG seven-pass G-type receptor 1 | 7656967 |
| cadherin EGF LAG seven-pass G-type receptor 2 | 13325064 |
| cadherin EGF LAG seven-pass G-type receptor 3 | 145309304 |
| cadherin related 23 isoform 1 precursor | 189571674 |
| cadherin related 23 isoform 2 precursor | 16507964 |
| calcitonin isoform CGRP preproprotein | 76880478 |
| calcium activated chloride channel 1 precursor | 110611231 |
| calcium activated chloride channel 2 | 5729769 |
| calcium activated nucleotidase 1 | 20270339 |
| calcium binding atopy-related autoantigen 1 | 116256483 |
| calcium channel, voltage-dependent, alpha 2/delta subunit 3 | 54112397 |
| calcium channel, voltage-dependent, beta 2 subunit isoform 7 | 45359834 |
| calcium channel, voltage-dependent, N type, alpha 1B subunit | 4502523 |
| hypothetical protein LOC100131098; calcium channel, voltage-dependent, L type, alpha 1C subunit | 120433602 |
| calcium channel, voltage-dependent, L type, alpha 1D subunit | 4502527 |
| calcium-transporting ATPase 2C1 isoform 1d | 48762689 |
| calmegin | 4758004 |
| calnexin precursor | 10716563 |
| carbohydrate (N-acetylglucosamine-6-O) sulfotransferase 2 | 27369497 |
| carboxypeptidase A2 (pancreatic) | 217416390 |
| carboxypeptidase D precursor | 22202611 |
| inactive carboxypeptidase-like protein X2 precursor | 223005864 |
| carnitine palmitoyltransferase 1A isoform 1 | 73623030 |
| carnitine palmitoyltransferase 1C | 22748777 |
| cartilage intermediate layer protein | 192449445 |
| cartilage linking protein 1 | 4503053 |
| cat eye syndrome chromosome region, candidate 6 | 13994236 |
| cat eye syndrome critical region protein 1 isoform a precursor | 29029550 |
| cauxin | 21450749 |
| CD109 | 115529484 |
| CD163 antigen isoform b | 44889963 |
| CD1A antigen precursor | 110618224 |
| CD1B antigen, b polypeptide | 4502645 |
| CD2 antigen (p50), sheep red blood cell receptor | 156071472 |
| CD209 antigen | 10863957 |
| CD276 antigen isoform a | 67188443 |
| CD27-binding (Siva) protein isoform 1 | 11277468 |
| CD2-associated protein | 11321634 |
| CD40 ligand | 4557433 |
| CD46 antigen, complement regulatory protein isoform 1 precursor | 27502402 |
| CD97 antigen isoform 2 precursor | 17978489 |
| CDC14 homolog A isoform 2 | 15451931 |
| cell death-regulatory protein GRIM19 | 260763955 |
| cellular repressor of E1A-stimulated genes | 4503037 |
| ceramide kinase isoform a | 20336726 |
| chemokine (C-X-C motif) ligand 12 | 10834988 |
| chemokine (C-X-C motif) ligand 16 | 154816176 |
| chemokine-like factor superfamily 5 isoform a | 19923993 |
| chitinase 3-like 1 | 144226251 |
| chitinase domain containing 1 | 218083142 |
| chitotriosidase | 4502809 |
| H(+)/Cl(-) exchange transporter 4 | 153252026 |
| chloride intracellular channel 6 | 27894378 |
| cholinergic receptor, nicotinic, alpha polypeptide 5 | 33589827 |
| chondroitin sulfate proteoglycan 2 (versican) | 21361116 |
| chondroitin sulfate proteoglycan 6 (bamacan) | 4885399 |
| chordin-like 1 | 34147715 |
| chromosome 20 open reading frame 70 | 45592961 |
| chromosome 6 open reading frame 10 | 116256485 |
| chromosome 6 open reading frame 71 | 42794271 |
| chromosome 9 open reading frame 36 | 153791826 |
| claudin 18 isoform 2 | 50345279 |
| clusterin isoform 1 | 42716297 |
| coagulation factor II precursor | 4503635 |
| coagulation factor V precursor | 105990535 |
| coagulation factor XIII A1 subunit precursor | 119395709 |
| coagulation factor XIII B subunit precursor | 110611237 |
| coiled-coil domain containing 109A | 24308400 |
| collagen and calcium binding EGF domains 1 | 39930511 |
| collagen, type V, alpha 3 preproprotein | 110735435 |
| collagen, type VIII, alpha 2 | 32964830 |
| collagen, type XII, alpha 1 short isoform precursor | 93141049 |
| collagen, type XIV, alpha 1 | 55743096 |
| collagen, type XXI, alpha 1 precursor | 18780273 |
| collagen, type XXII, alpha 1 | 40805823 |
| collagen, type XXIII, alpha 1 | 29725624 |
| collagen, type XXIV, alpha 1 | 115392133 |
| collagen, type XXVII, alpha 1 | 32140760 |
| collagen, type XXVIII precursor | 154759255 |
| collectin sub-family member 10 | 256017186 |
| collectin sub-family member 12 isoform I | 18641360 |
| complement component (3d/Epstein Barr virus) receptor 2 | 42544177 |
| complement component 1, q subcomponent, A chain precursor | 7705753 |
| complement component 1, r subcomponent | 66347875 |
| complement component 1, s subcomponent | 4502495 |
| complement component 2 precursor | 14550407 |
| complement component 3 precursor | 115298678 |
| complement component 4 binding protein, alpha chain precursor | 4502503 |
| complement component 4A preproprotein | 67190748 |
| complement component 8, alpha polypeptide precursor | 4557389 |
| complement component 9 | 4502511 |
| complement receptor 1 isoform S precursor | 86793109 |
| contactin 1 isoform 1 precursor | 28373117 |
| contactin 2 precursor | 4827022 |
| contactin 4 isoform a precursor | 28373122 |
| contactin 6 | 7657361 |
| contactin associated protein 1 | 4505463 |
| corneodesmosin precursor | 67782356 |
| cortistatin preproprotein | 41327683 |
| crumbs 3 isoform a precursor | 21040241 |
| crumbs homolog 1 precursor | 41327708 |
| CUB and Sushi multiple domains 2 | 92110053 |
| cysteinyl leukotriene receptor 2 | 9966851 |
| cystic fibrosis transmembrane conductance regulator | 90421313 |
| cytokine receptor-like factor 3 | 156142186 |
| cytoskeleton-associated protein 4 | 19920317 |
| dachsous 1 precursor | 16933557 |
| DC-STAMP domain containing 1 | 219521924 |
| death-inducing-protein | 67763814 |
| decorin isoform a preproprotein | 4503271 |
| defensin, beta 116 | 83582800 |
| deoxyribonuclease I-like 2 | 4503347 |
| dermcidin preproprotein | 16751921 |
| desert hedgehog preproprotein | 19482158 |
| desmocollin 1 isoform Dsc1b preproprotein | 4826702 |
| desmocollin 2 isoform Dsc2b preproprotein | 13435366 |
| desmocollin 3 isoform Dsc3b preproprotein | 148539848 |
| desmoglein 1 preproprotein | 119703744 |
| desmoglein 3 preproprotein | 119964718 |
| dickkopf homolog 3 precursor | 40548393 |
| dipeptidyl peptidase 10 isoform long | 52426756 |
| dipeptidylpeptidase IV | 18765694 |
| dispatched A | 25952134 |
| Down syndrome cell adhesion molecule isoform CHD2-42 precursor | 20127422 |
| Down syndrome cell adhesion molecule like 1 | 21359935 |
| protein dpy-19 homolog 3 | 289666758 |
| dual oxidase 1 precursor | 20149640 |
| dysferlin | 4503431 |
| ectodysplasin A receptor | 11641231 |
| EGF-like repeats and discoidin I-like domains 3 | 31317224 |
| elafin preproprotein | 4505787 |
| electron-transferring-flavoprotein dehydrogenase | 119703746 |
| Ellis van Creveld syndrome protein | 24497531 |
| endoglin precursor | 4557555 |
| endoplasmic reticulum to nucleus signalling 2 | 145312265 |
| endothelial cell adhesion molecule | 148664205 |
| endothelin receptor type A | 4503465 |
| endothelin receptor type B isoform 1 | 4557547 |
| ephrin receptor EphA5 isoform a | 221625401 |
| ephrin receptor EphA7 | 4758282 |
| ephrin receptor EphB3 precursor | 17975768 |
| ephrin receptor EphB4 precursor | 32528301 |
| epidermal growth factor (beta-urogastrone) | 166362728 |
| epidermal growth factor receptor isoform a | 29725609 |
| epithelial membrane protein 3 | 4503563 |
| ER degradation enhancer, mannosidase alpha-like 1 | 7662002 |
| transmembrane channel-like protein 6 | 187608784 |
| extracellular matrix protein 2 precursor | 4557543 |
| factor for adipocyte differentiation 158 | 19923729 |
| family with sequence similarity 20, member B | 7662150 |
| family with sequence similarity 62 (C2 domain containing), member B | 45387945 |
| Fanconi anemia complementation group D2 isoform b | 66528888 |
| farnesyl-diphosphate farnesyltransferase 1 | 67089147 |
| FAT tumor suppressor 2 precursor | 13787217 |
| FAT tumor suppressor homolog 4 | 165932370 |
| fatty acid desaturase 2 | 4758334 |
| Fc fragment of IgG binding protein | 154146262 |
| Fc receptor-like 5 | 157694526 |
| fibrillin 1 precursor | 281485550 |
| fibrinogen, beta chain preproprotein | 70906435 |
| fibrinogen-like 1 precursor | 42544189 |
| fibroblast activation protein, alpha subunit | 16933540 |
| fibroblast growth factor 17 precursor | 4503693 |
| fibroblast growth factor 18 precursor | 4503695 |
| fibroblast growth factor binding protein 3 | 190341093 |
| fibroblast growth factor receptor 1 isoform 2 precursor | 13186251 |
| fibrocystin L | 126116589 |
| fibronectin 1 isoform 3 preproprotein | 16933542 |
| fms-related tyrosine kinase 1 | 156104876 |
| folate hydrolase 1 isoform 2 | 62548858 |
| follistatin-like 4 | 54792136 |
| FRAS1 related extracellular matrix 1 | 122056683 |
| Fraser syndrome 1 | 256000767 |
| frizzled 3 | 8393378 |
| fukutin-related protein | 13236528 |
| furin preproprotein | 4505579 |
| G protein-binding protein CRFG | 55953087 |
| G protein-coupled receptor 101 | 16876435 |
| G protein-coupled receptor 107 | 56711308 |
| G protein-coupled receptor 147 | 11545887 |
| G protein-coupled receptor 37 | 4885323 |
| G protein-coupled receptor 74 isoform 2 | 16604258 |
| G protein-coupled receptor 81 | 14211851 |
| galectin 3 | 115430223 |
| galectin 4 | 5453712 |
| gamma-aminobutyric acid (GABA) A receptor, beta 1 precursor | 194097327 |
| gamma-aminobutyric acid (GABA) A receptor, gamma 3 | 110347416 |
| gamma-aminobutyric acid (GABA) B receptor 1 isoform b precursor | 11497612 |
| gamma-glutamyl hydrolase precursor | 4503987 |
| ganglioside-induced differentiation-associated protein 1-like | 30581160 |
| gap junction protein, alpha 7, 45kDa (connexin 45) | 69122473 |
| GCN1 general control of amino-acid synthesis 1-like 1 | 54607053 |
| GDNF family receptor alpha 3 preproprotein | 22035694 |
| genethonin 1 | 4503977 |
| gliomedin | 98986321 |
| glucagon-like peptide 1 receptor | 166795283 |
| glucose transporter protein 10 isoform a | 190684655 |
| glutamate receptor KA2 precursor | 29029597 |
| glutamate receptor, ionotropic, delta 2 | 157384977 |
| glutamate receptor, ionotropic, kainate 1 isoform 2 precursor | 28416444 |
| glutamate receptor, metabotropic 2 precursor | 66529100 |
| glutamate receptor, metabotropic 3 precursor | 46358417 |
| glutamate receptor, metabotropic 4 | 4504141 |
| glutamate receptor, metabotropic 6 precursor | 110611176 |
| glycine receptor, beta | 4504023 |
| glycoprotein M6A isoform 3 | 42476105 |
| glycosyltransferase 8 domain containing 1 | 23510346 |
| glypican 1 precursor | 167001141 |
| glypican 6 precursor | 5031719 |
| golgi autoantigen, golgin subfamily b, macrogolgin (with trans 1 16.1 0.0 4758454 | 148596984 |
| golgi phosphoprotein 4 | 7657138 |
| golgi reassembly stacking protein 2 | 29826294 |
| GPI deacylase | 46240862 |
| G-protein coupled receptor 116 | 148719673 |
| G-protein-coupled receptor kinase 7 | 21166359 |
| growth and transformation-dependent protein | 49355721 |
| growth arrest-specific 2 like 1 isoform a | 23065526 |
| growth arrest-specific 6 | 4557617 |
| growth hormone 1 isoform 4 | 13027818 |
| guanylate cyclase 2D, membrane (retina-specific) | 4504217 |
| heat shock 105kD | 42544159 |
| heat shock 27kDa protein 1 | 4504517 |
| heat shock 27kDa protein family, member 7 (cardiovascular) | 7657202 |
| heat shock 70kD protein 12B | 31317303 |
| heat shock 70kDa protein 1A | 194248072 |
| heat shock 70kDa protein 2 | 13676857 |
| heat shock 70kDa protein 5 (glucose-regulated protein, 78kDa) | 7657202 |
| heat shock 70kDa protein 9B precursor | 24234688 |
| heat shock protein 90kDa alpha (cytosolic), class A member 1 I | 153792590 |
| hematopoietic protein 1 | 34485727 |
| hemicentin 1 | 118572606 |
| heparan sulfate 6-O-sulfotransferase 2 | 116295254 |
| heparan sulfate proteoglycan 2 | 126012571 |
| hepatocyte cell adhesion molecule | 121674801 |
| hephaestin isoform b | 7662254 |
| HERV-H LTR-associating 2 | 5901964 |
| HGF activator preproprotein | 4504383 |
| hornerin | 57864582 |
| hyaluronan-mediated motility receptor isoform b | 217416398 |
| hyaluronoglucosaminidase 4 | 166235888 |
| potassium/sodium hyperpolarization-activated cyclic nucleotide-gated channel 1 | 116325989 |
| protein furry homolog | 117606355 |
| coiled-coil domain-containing protein 136 | 115511012 |
| hypothetical protein LOC118663 | 50345833 |
| hypothetical protein LOC120406 | 210147446 |
| hypothetical protein LOC121793 | 22748709 |
| transmembrane protein C15orf27 | 118442841 |
| hypothetical protein LOC124220 | 94536866 |
| hypothetical protein LOC132720 | 22748853 |
| hypothetical protein LOC133558 | 154240671 |
| hypothetical protein LOC136263 | 21687171 |
| hypothetical protein LOC138311 | 22748891 |
| hypothetical protein LOC145748 | 40288201 |
| hypothetical protein LOC147744 | 21040263 |
| glycerol-3-phosphate acyltransferase 2, mitochondrial | 116812614 |
| hypothetical protein LOC150771 isoform 2 | 57164960 |
| hypothetical protein LOC153396 | 23503279 |
| fatty-acid amide hydrolase 2 | 195972892 |
| hypothetical protein LOC160335 | 22749211 |
| hypothetical protein LOC160518 | 122891862 |
| nesprin-3 | 145580592 |
| leucine-rich repeat neuronal protein 4 precursor | 188536110 |
| hypothetical protein LOC166929 | 22749271 |
| T cell immunoreceptor with Ig and ITIM domains precursor | 256600228 |
| transmembrane anterior posterior transformation protein 1 homolog | 130977756 |
| hypothetical protein LOC205717 | 114431248 |
| hypothetical protein LOC221061 | 63025206 |
| hypothetical protein LOC221481 | 31542280 |
| hypothetical protein LOC222658 | 40255182 |
| protein sel-1 homolog 3 | 154689719 |
| hypothetical protein LOC23251 | 54606888 |
| hypothetical protein LOC23281 isoform b | 57768564 |
| transmembrane protein C9orf5 | 110681719 |
| hypothetical protein LOC253012 isoform 1 | 86439957 |
| hypothetical protein LOC256471 | 22749525 |
| hypothetical protein LOC257044 | 28603838 |
| hypothetical protein LOC25871 isoform a | 68299770 |
| hypothetical protein LOC25895 isoform a | 45827721 |
| transmembrane protein C16orf54 | 148233642 |
| hypothetical protein LOC284099 | 45580704 |
| hypothetical protein LOC284114 | 30425460 |
| hypothetical protein LOC285093 | 134288863 |
| hypothetical protein LOC340061 | 38093659 |
| hypothetical protein LOC374872 | 38348368 |
| hypothetical protein LOC374977 | 223278410 |
| hypothetical protein LOC387104 | 59806361 |
| protein shisa-6 homolog isoform 1 | 211904177 |
| family with sequence similarity 69, member A | 165932391 |
| hypothetical protein LOC389763 | 48717285 |
| hypothetical protein LOC399947 | 283046807 |
| patched domain-containing protein C6orf138 | 257743475 |
| hypothetical protein LOC51300 | 59710109 |
| hypothetical protein LOC54675 | 10092647 |
| transmembrane protein 214 isoform 1 | 134152721 |
| hypothetical protein LOC54914 | 150456451 |
| hypothetical protein LOC54978 | 31542711 |
| required for meiotic nuclear division protein 1 homolog | 157388927 |
| hypothetical protein LOC55640 | 190341091 |
| hypothetical protein LOC55780 | 62988331 |
| hypothetical protein LOC57221 | 154350241 |
| hypothetical protein LOC57464 | 55742705 |
| hypothetical protein LOC57535 | 38569482 |
| hypothetical protein LOC57609 | 55749758 |
| hypothetical protein LOC60686 | 195233774 |
| family with sequence similarity 38, member B | 257900451 |
| hypothetical protein LOC651928 | 91206444 |
| fer-1-like protein 6 | 119120884 |
| prenylcysteine oxidase-like precursor | 226442763 |
| hypothetical protein LOC79794 | 13376060 |
| hypothetical protein LOC79820 | 51339295 |
| coiled-coil domain-containing protein 102B | 148233522 |
| hypothetical protein LOC79953 | 13376347 |
| hypothetical protein LOC80067 | 100816140 |
| PGAP2-interacting protein | 156151386 |
| CST complex subunit CTC1 | 155029539 |
| hypothetical protein LOC80208 | 93204888 |
| hypothetical protein LOC84187 | 93277068 |
| hypothetical protein LOC84899 | 118766330 |
| major facilitator superfamily domain-containing protein 5 isoform 2 precursor | 283046669 |
| hypothetical protein LOC91775 | 21450781 |
| hypothetical protein LOC92126 | 23943787 |
| hypothetical protein LOC9671 | 55749667 |
| hypothetical protein LOC9703 | 57242774 |
| hypothetical protein LOC9865 | 76880480 |
| hypothetical protein LOC9870 | 87116668 |
| transmembrane protein 97 | 109948302 |
| hypoxia-inducible factor prolyl 4-hydroxylase isoform c | 64085083 |
| IKK interacting protein isoform 1 | 24233517 |
| immunoglobulin J chain | 21489959 |
| immunoglobulin superfamily, member 1 isoform 1 | 45505167 |
| immunoglobulin superfamily, member 3 isoform 2 | 55953135 |
| immunoglobulin superfamily, member 6 | 93004094 |
| IMP2 inner mitochondrial membrane protease-like | 14211845 |
| importin 7 | 5453998 |
| inflammation-related G protein-coupled receptor EX33 | 9966839 |
| type II inositol-1,4,5-trisphosphate 5-phosphatase precursor | 113722125 |
| insulin-like 6 precursor | 38569396 |
| insulin-like growth factor 1 receptor precursor | 4557665 |
| insulin-like growth factor 2 receptor | 119964726 |
| insulin-like growth factor binding protein 4 precursor | 62243290 |
| insulin-like growth factor binding protein 7 | 4504619 |
| insulin-like growth factor binding protein-like 1 | 56090548 |
| integral membrane protein 1 | 22749415 |
| integrin alpha 4 precursor | 67191027 |
| integrin alpha chain, alpha 6 | 119395742 |
| integrin beta 4 isoform 1 precursor | 54607035 |
| integrin, alpha 1 precursor | 31657142 |
| integrin, beta 2 precursor | 89191865 |
| integrin, beta 6 | 9625002 |
| interferon epsilon 1 | 28882045 |
| interferon, alpha 2 | 11067751 |
| interferon, beta 1, fibroblast | 4504603 |
| interleukin 1 family, member 8 isoform 2 | 27894313 |
| interleukin 1 receptor accessory protein isoform 1 | 4504661 |
| interleukin 1 receptor accessory protein-like 1 | 7657232 |
| interleukin 1 receptor, type II precursor | 4758598 |
| interleukin 15 receptor, alpha isoform 1 precursor | 4504649 |
| interleukin 16 isoform 2 | 148833504 |
| interleukin 17F precursor | 16418375 |
| interleukin 20 precursor | 31083166 |
| interleukin 20 receptor, alpha | 31083156 |
| interleukin 5 receptor, alpha isoform 1 precursor | 28559021 |
| interphotoreceptor matrix proteoglycan 1 | 6631090 |
| interphotoreceptor matrix proteoglycan 2 | 57242793 |
| ion transporter protein | 48526516 |
| jagged 1 precursor | 4557679 |
| kallikrein 11 isoform 1 preproprotein | 5803199 |
| anosmin-1 precursor | 119395746 |
| kelch-like 11 | 8922528 |
| KIAA0319 | 134304840 |
| transmembrane protein 131-like isoform 2 | 187608809 |
| KIAA1913 | 29789291 |
| kinase insert domain receptor (a type III receptor tyrosine ki1 | 11321597 |
| kinectin 1 | 33620775 |
| KIT ligand isoform a precursor | 4580420 |
| lactase-like | 110681710 |
| lactase-phlorizin hydrolase preproprotein | 32481206 |
| lactotransferrin | 54607120 |
| laeverin | 194239713 |
| laminin alpha 2 subunit precursor | 28559088 |
| laminin alpha 3 subunit isoform 1 | 38045910 |
| laminin alpha 5 | 21264602 |
| laminin, alpha 1 precursor | 38788416 |
| laminin, alpha 4 precursor | 157419122 |
| laminin, beta 4 | 143770880 |
| laminin, gamma 1 precursor | 145309326 |
| laminin, gamma 2 isoform a precursor | 157419138 |
| latent transforming growth factor beta binding protein 3 | 18497288 |
| latent transforming growth factor beta binding protein 4 | 110347412 |
| latrophilin 2 precursor | 6912464 |
| LEM domain containing 1 | 56676324 |
| LEM domain containing 2 | 31044432 |
| lemur tyrosine kinase 2 | 38016937 |
| leptin receptor isoform 2 | 51093379 |
| leucine rich repeat and fibronectin type III domain containing | 31542244 |
| leucine rich repeat containing 22 | 62988340 |
| leucine rich repeat containing 5 | 34222199 |
| leucine-rich repeat neuronal protein 1 precursor | 153791330 |
| leucine rich repeat neuronal 5 precursor | 42544231 |
| leucine-rich repeat LGI family, member 2 | 21313638 |
| leucine-rich repeats and immunoglobulin-like domains 1 | 54607118 |
| leucine-rich repeats and immunoglobulin-like domains 2 | 7662320 |
| leucyl/cystinyl aminopeptidase isoform 1 | 61742777 |
| lipophilin A precursor | 5729907 |
| longevity assurance gene 1 isoform 1 | 11641421 |
| low density lipoprotein-related protein 1 | 126012562 |
| low density lipoprotein-related protein 2 | 126012573 |
| LPLUNC1 protein precursor | 40807482 |
| lymphocyte antigen 75 | 144446030 |
| lymphocyte-activation protein 3 precursor | 167614500 |
| LysM, putative peptidoglycan-binding, domain containing 3 | 84370276 |
| lysophosphatidylglycerol acyltransferase 1 | 7661996 |
| lysosomal acid phosphatase 2 precursor | 4557010 |
| lysosomal-associated membrane protein 2 precursor | 7669503 |
| lysozyme precursor | 4557894 |
| lysyl oxidase-like 1 preproprotein | 67782346 |
| macrophage stimulating 1 (hepatocyte growth factor-like) | 205277383 |
| maestro | 187761375 |
| major histocompatibility complex, class I, B | 17986001 |
| major histocompatibility complex, class I, C precursor | 52630342 |
| major histocompatibility complex, class II, DO beta precursor | 4504403 |
| maltase-glucoamylase | 221316699 |
| MAM domain containing glycosylphosphatidylinositol anchor 1 | 24111244 |
| matrix metalloproteinase 14 preproprotein | 4826834 |
| matrix metalloproteinase 15 preproprotein | 4505211 |
| matrix metalloproteinase 16 isoform 1 preproprotein | 13027802 |
| matrix metalloproteinase 2 preproprotein | 11342666 |
| matrix metalloproteinase 27 | 73808268 |
| matrix metalloproteinase 3 preproprotein | 4505217 |
| matrix, extracellular phosphoglycoprotein with ASARM motif (bo1 6.1 0.0 9910430 | 9910430 |
| MEGF10 protein | 14192943 |
| membralin isoform 1 | 74229025 |
| membrane associated guanylate kinase, WW and PDZ domain contai | 74272284 |
| membrane metallo-endopeptidase | 116256331 |
| membrane metallo-endopeptidase-like 1 | 239049391 |
| membrane protein, palmitoylated 5 | 38570142 |
| membrane protein, palmitoylated 6 | 21361598 |
| membrane-associated transporter protein isoform a | 61635915 |
| MER receptor tyrosine kinase precursor | 66932918 |
| mesothelin isoform 2 preproprotein | 53988380 |
| microfibrillar-associated protein 3-like isoform 1 | 153792329 |
| microsomal glutathione S-transferase 2 | 4505181 |
| mitochondrial outer membrane protein TOM40 | 5174723 |
| motile sperm domain containing 3 isoform a | 13122608 |
| motilin isoform 1 preproprotein | 4557034 |
| mucin 16 | 83367077 |
| mucin 4 isoform a | 257471027 |
| mucin 4 isoform e | 112382231 |
| multiple C2-domains with two transmembrane regions 1 isoform S | 50582996 |
| multiple C2-domains with two transmembrane regions 2 | 227496440 |
| myelin oligodendrocyte glycoprotein isoform alpha1 precursor | 56788381 |
| myeloid cell leukemia sequence 1 isoform 2 | 33519458 |
| N system amino acid transporter NAT-1 | 289577102 |
| Na+/K+ -ATPase alpha 2 subunit proprotein | 4502271 |
| Na+/K+ -ATPase alpha 4 subunit isoform 1 | 153946397 |
| Na+/K+ -ATPase beta 3 subunit | 4502281 |
| N-acetylated alpha-linked acidic dipeptidase 2 | 4885505 |
| N-acetylglucosamine-1-phosphate transferase | 38202211 |
| NADPH oxidase, EF hand calcium-binding domain 5 | 20127624 |
| nardilysin (N-arginine dibasic convertase) | 156071450 |
| natriuretic peptide receptor A/guanylate cyclase A (atrionatri | 167830411 |
| natriuretic peptide receptor B precursor | 4580422 |
| neogenin homolog 1 | 157311649 |
| nerve growth factor, beta polypeptide precursor | 70995319 |
| nesprin 1 isoform beta | 154277118 |
| nesprin 1 isoform longer | 23097308 |
| netrin 4 | 93204871 |
| netrin G2 | 190014607 |
| netrin-G1 ligand | 51317373 |
| neural cell adhesion molecule 2 precursor | 33519481 |
| neurexin 1 isoform alpha precursor | 14149613 |
| neurexin 2 isoform alpha-1 precursor | 14211536 |
| neurexin 3 isoform alpha precursor | 21070969 |
| neurofascin precursor | 89903008 |
| neuroligin 2 | 30840978 |
| neuronal cell adhesion molecule isoform B precursor | 81158224 |
| neuronal pentraxin II | 28195384 |
| neuropeptide Y | 4505449 |
| neurotrophin 3 precursor | 4505469 |
| NHL repeat containing 2 | 42476013 |
| nicotinamide nucleotide transhydrogenase | 122939153 |
| nicotinic acetylcholine receptor beta 1 subunit precursor | 41327726 |
| nicotinic acetylcholine receptor delta polypeptide precursor | 4557461 |
| Niemann-Pick disease, type C1 | 255652944 |
| NMDA receptor 1 isoform NR1-2 precursor | 11038635 |
| N-methyl-D-aspartate receptor subunit 2A precursor | 4504125 |
| nodal modulator 3 | 51944969 |
| notch4 preproprotein | 55770876 |
| odz, odd Oz/ten-m homolog 1 | 110347400 |
| olfactomedin-like 1 | 284172520 |
| olfactory receptor, family 2, subfamily L, member 2 | 52317202 |
| olfactory receptor, family 4, subfamily C, member 13 | 284172354 |
| olfactory receptor, family 5, subfamily K, member 3 | 53933278 |
| olfactory receptor, family 5, subfamily K, member 4 | 53933287 |
| olfactory receptor, family 5, subfamily M, member 9 | 52317128 |
| olfactory receptor, family 51, subfamily G, member 2 | 52546689 |
| olfactory receptor, family 52, subfamily E, member 4 | 52353252 |
| olfactory receptor, family 56, subfamily A, member 4 | 94967016 |
| olfactory receptor, family 6, subfamily C, member 4 | 53828708 |
| olfactory receptor, family 7, subfamily G, member 2 | 52353346 |
| olfactory receptor, family 8, subfamily D, member 2 | 50897292 |
| olfactory receptor, family 8, subfamily G, member 5 | 52353354 |
| olfactory receptor, family 8, subfamily J, member 1 | 52353298 |
| OMA1 homolog, zinc metallopeptidase | 21686999 |
| optic atrophy 1 isoform 6 | 18860841 |
| organic anion transporting polypeptide A isoform a | 10835099 |
| organic solute transporter beta | 269914183 |
| otoancorin isoform 1 | 77404409 |
| ovochymase 1 | 110815798 |
| protein CIP2A | 190194355 |
| pad-1-like | 45827701 |
| palate, lung and nasal epithelium carcinoma associated protein | 18765705 |
| palmitoylated membrane protein 3 | 21536464 |
| palmitoylated membrane protein 7 | 111154074 |
| pancreatic carboxypeptidase A1 precursor | 4502997 |
| pannexin 1 | 39995064 |
| PDZ domain containing 2 | 87196343 |
| peptidylprolyl isomerase A isoform 1 | 10863927 |
| peroxisomal biogenesis factor 11A | 4505717 |
| peroxisomal biogenesis factor 12 | 4505721 |
| phosphatidic acid phosphatase type 2 domain containing 2 | 66773040 |
| phosphatidic acid phosphatase type 2A isoform 2 | 29171738 |
| phosphatidylinositol glycan, class O isoform 1 | 23397648 |
| phosphatidylinositol glycan, class Q isoform 2 | 22538450 |
| phosphatidylserine synthase 2 | 13540555 |
| phospholipase A2, group VI isoform b | 52486251 |
| plasma carboxypeptidase B2 isoform b | 126273559 |
| plasma membrane calcium ATPase 1 isoform 1b | 48255945 |
| plasma membrane calcium ATPase 2 isoform b | 48255949 |
| plasma membrane calcium ATPase 3 isoform 3a | 48255953 |
| plasma membrane calcium ATPase 4 isoform 4b | 48255957 |
| plasmalemma vesicle associated protein | 13775238 |
| plasticity related gene 1 | 33636722 |
| platelet/endothelial cell adhesion molecule (CD31 antigen) | 110347451 |
| pleckstrin and Sec7 domain containing 2 | 14150035 |
| pleckstrin homology domain containing, family H | 55741447 |
| plexin A1 | 262118282 |
| plexin B3 | 29336063 |
| plexin domain containing 2 precursor | 40255005 |
| PLSC domain containing protein | 87116681 |
| polycystic kidney disease 2-like 1 | 33598944 |
| polycystic kidney disease 2-like 2 | 116812588 |
| polycystin 1 isoform 2 precursor | 205360962 |
| polycystin 2 | 4505835 |
| polymeric immunoglobulin receptor | 31377806 |
| polypeptide N-acetylgalactosaminyltransferase 1 | 13124891 |
| polypeptide N-acetylgalactosaminyltransferase 3 | 153266878 |
| polypeptide N-acetylgalactosaminyltransferase 6 | 115298684 |
| potassium channel tetramerisation domain containing 10 | 13994353 |
| potassium channel tetramerisation domain containing 12 | 19923973 |
| potassium channel tetramerisation domain containing 16 | 55926200 |
| potassium channel tetramerisation domain containing 5 | 9506651 |
| potassium channel, subfamily K, member 5 | 4504851 |
| potassium channel, subfamily T, member 1 | 240255505 |
| potassium channel, subfamily T, member 2 | 41349443 |
| potassium family, subfamily K, member 15 | 11641275 |
| potassium inwardly-rectifying channel J5 | 24797141 |
| potassium voltage-gated channel KQT-like protein 3 | 4758630 |
| potassium voltage-gated channel, Shab-related subfamily, member 2 | 27436974 |
| potassium voltage-gated channel, shaker-related subfamily, beta member 2 | 4504825 |
| potassium voltage-gated channel, Shal-related subfamily, member 1 | 27436981 |
| potassium voltage-gated channel, subfamily H, member 4 | 6912446 |
| potassium voltage-gated channel, subfamily H, member 6 | 27886651 |
| potassium voltage-gated channel, subfamily H, member 7 | 27886653 |
| sushi, nidogen and EGF-like domain-containing protein 1 precursor | 122937283 |
| transmembrane protein C12orf51 | 292781435 |
| heparan-alpha-glucosaminide N-acetyltransferase | 150378452 |
| von Willebrand factor D and EGF domain-containing protein precursor | 209571555 |
| sushi domain-containing protein 5 precursor | 150378552 |
| fragile site-associated protein | 150378498 |
| von Willebrand factor A domain-containing protein 5B2 | 222352129 |
| PREDICTED: hypothetical protein XP_374010 | 88953667 |
| macrophage expressed gene 1 precursor | 133505169 |
| PREDICTED: otogelin | 239744300 |
| SCO-spondin precursor | 134031945 |
| PREDICTED: similar to CG13409-PA | 153792042 |
| immunoglobulin superfamily, member 9B | 148886752 |
| T cell-interacting, activating receptor on myeloid cells 1 | 208879429 |
| pregnancy-associated plasma protein A preproprotein | 38045915 |
| pregnancy-zone protein | 162809334 |
| prenylcysteine oxidase 1 | 166795301 |
| proacrosin binding protein sp32 precursor | 17999524 |
| procollagen, type III, alpha 1 | 4502951 |
| prohibitin | 4505773 |
| prolactin regulatory element binding protein | 7019503 |
| prolactin-induced protein | 4505821 |
| proline rich 4 (lacrimal) | 154448886 |
| proline rich Gla (G-carboxyglutamic acid) 4 (transmembrane) | 13129074 |
| proline-rich protein BstNI subfamily 1 isoform 1 precursor | 41349482 |
| prolyl 4-hydroxylase, beta subunit | 20070125 |
| prominin 1 | 5174387 |
| proopiomelanocortin preproprotein | 80861463 |
| prostaglandin F2 receptor negative regulator | 41152506 |
| prostatic acid phosphatase precursor | 6382064 |
| PRotein Associated with Tlr4 | 22749479 |
| protein C (inactivator of coagulation factors Va and VIIIa) | 4506115 |
| protein disulfide isomerase-associated 2 | 94966757 |
| protein disulfide isomerase-associated 5 | 5803121 |
| protein disulfide isomerase-associated 6 | 5031973 |
| protein tyrosine phosphatase, receptor type, B precursor | 157952213 |
| protein tyrosine phosphatase, receptor type, H precursor | 241896924 |
| protein tyrosine phosphatase, receptor type, K precursor | 18860902 |
| protein tyrosine phosphatase, receptor type, N precursor | 4506321 |
| protein Z, vitamin K-dependent plasma glycoprotein | 4506121 |
| proteoglycan 4 | 67190163 |
| protocadherin 10 isoform 1 precursor | 14589916 |
| protocadherin 11 Y-linked isoform c | 14589946 |
| protocadherin 12 precursor | 7706113 |
| protocadherin 17 | 94538350 |
| protocadherin 7 isoform b precursor | 14589933 |
| protocadherin alpha 1 isoform 2 precursor | 14165400 |
| protocadherin alpha 10 isoform 1 precursor | 9256574 |
| protocadherin alpha 11 isoform 1 precursor | 9256576 |
| protocadherin alpha 2 isoform 2 precursor | 14165405 |
| protocadherin alpha subfamily C, 2 isoform 1 precursor | 9256600 |
| protocadherin beta 14 precursor | 9256608 |
| protocadherin beta 15 precursor | 9256610 |
| protocadherin beta 4 precursor | 9256616 |
| protocadherin beta 5 precursor | 7661662 |
| protocadherin gamma subfamily A, 1 isoform 1 precursor | 11056032 |
| protocadherin gamma subfamily A, 2 isoform 2 precursor | 14196462 |
| protocadherin gamma subfamily A, 3 isoform 2 precursor | 14196465 |
| protocadherin gamma subfamily A, 6 isoform 2 precursor | 14196474 |
| proto-oncogene c-ros-1 protein precursor | 19924165 |
| PTPRF interacting protein alpha 2 | 29171755 |
| PTPRF interacting protein alpha 3 | 32189362 |
| purinergic receptor P2X2 isoform B | 28416923 |
| purinergic receptor P2X-like 1, orphan receptor | 157419148 |
| purinergic receptor P2Y12 | 12232483 |
| putative capacitative calcium channel | 9966865 |
| quiescin Q6 isoform a | 13325075 |
| receptor expression enhancing protein 3 | 47679089 |
| receptor for egg jelly-like protein precursor | 5174633 |
| reelin isoform b | 27436940 |
| regeneration associated muscle protease isoform b | 50659100 |
| retbindin | 13899247 |
| reticulon 3 isoform b | 41393608 |
| reticulon 4 isoform A | 24431935 |
| reticulon 4 receptor-like 1 | 30425553 |
| Rho-associated, coiled-coil containing protein kinase 2 | 41872583 |
| rhodopsin kinase | 4506529 |
| rhomboid family 1 | 190341097 |
| rhomboid, veinlet-like 6 isoform 1 | 93352556 |
| ribophorin II precursor | 35493916 |
| E3 ubiquitin-protein ligase RNF149 precursor | 284447287 |
| roundabout homolog 4, magic roundabout | 17511435 |
| roundabout, axon guidance receptor, homolog 2 | 61888896 |
| R-spondin family, member 2 | 222446611 |
| ryanodine receptor 1 (skeletal) | 113204615 |
| ryanodine receptor 2 | 112799847 |
| ryanodine receptor 3 | 126032338 |
| RYK receptor-like tyrosine kinase isoform 1 | 54607020 |
| S100 calcium-binding protein A12 | 5032059 |
| S100 calcium-binding protein A4 | 4506765 |
| S100 calcium-binding protein A9 | 4506773 |
| Sad1 and UNC84 domain containing 1 | 71834868 |
| sarcoglycan, beta (43kDa dystrophin-associated glycoprotein) | 4506913 |
| sarcoma antigen NY-SAR-41 | 62243484 |
| scavenger receptor class B, member 2 | 5031631 |
| secreted modular calcium-binding protein 1 isoform 2 | 11545873 |
| secretin preproprotein | 11345450 |
| secretoglobin, family 1A, member 1 (uteroglobin) | 4507809 |
| seizure related 6 homolog | 148839280 |
| seizure related 6 homolog (mouse)-like precursor | 32261332 |
| semaphorin 3E | 6912650 |
| semaphorin 6B isoform 1 precursor | 19718778 |
| semenogelin I isoform a preproprotein | 4506883 |
| semenogelin II precursor | 4506885 |
| serpin peptidase inhibitor, clade A (alpha-1 antiproteinase, antitrypsin), member 1 | 50363221 |
| serpin peptidase inhibitor, clade A (alpha-1 antiproteinase, antitrypsin), member 12 | 27777657 |
| serpin peptidase inhibitor, clade A (alpha-1 antiproteinase, antitrypsin), member 10 | 7705879 |
| serpin peptidase inhibitor, clade A (alpha-1 antiproteinase, antitrypsin), member 9 | 110225347 |
| serpin peptidase inhibitor, clade C (antithrombin), member 1 | 4502261 |
| serine palmitoyltransferase subunit 1 isoform a | 5454084 |
| serine peptidase inhibitor, Kazal type 5 precursor | 74027261 |
| serum amyloid P component precursor | 4502133 |
| seven transmembrane domain protein | 14249166 |
| SHP2-interacting transmembrane adaptor protein | 7657577 |
| sialic acid binding immunoglobulin-like lectin-like protein 1 | 16506828 |
| sialyltransferase 6 isoform j | 5454060 |
| sidekick homolog 1 | 119220552 |
| signal peptide peptidase-like 2B isoform 2 | 41281782 |
| signal peptide, CUB domain, EGF-like 3 | 31377568 |
| single Ig IL-1R-related molecule | 205277445 |
| slit and trk like 1 protein | 40217817 |
| slit and trk like 3 protein | 40217820 |
| slit and trk like 4 protein | 27436867 |
| slit homolog 3 | 11321571 |
| small conductance calcium-activated potassium channel protein | 25777647 |
| sodium bicarbonate transporter 4 isoform a | 125987596 |
| sodium channel, voltage-gated, type I, alpha | 115583677 |
| sodium channel, voltage-gated, type III, alpha | 126362949 |
| sodium channel, voltage-gated, type IX, alpha | 4506813 |
| soluble adenylyl cyclase | 209976994 |
| soluble liver antigen/liver pancreas antigen isoform 2 | 267844904 |
| solute carrier family 10 (sodium/bile acid cotransporter family), member 5 | 58219066 |
| solute carrier family 10, member 3 | 9790143 |
| solute carrier family 12 (potassium/chloride transporters), member 9 | 31881740 |
| solute carrier family 12 (sodium/potassium/chloride transporters), member 2 | 4506975 |
| solute carrier family 13 (sodium-dependent citrate transporter), member 5 | 29171306 |
| solute carrier family 15 (oligopeptide transporter), member 1 | 4827008 |
| solute carrier family 16, member 10 | 18699730 |
| solute carrier family 16, member 5 | 4759116 |
| solute carrier family 17 (sodium phosphate), member 2 | 5031955 |
| solute carrier family 17 (sodium-dependent inorganic phosphate cotransporter), member 8 | 21322234 |
| solute carrier family 19 member 1 isoform a | 34808710 |
| solute carrier family 22 member 11 | 8923870 |
| solute carrier family 22 member 3 | 11415038 |
| solute carrier family 22 member 8 | 24497499 |
| solute carrier family 22, member 16 | 31542327 |
| solute carrier family 25 (mitochondrial thiamine pyrophosphate carrier), member 19 | 186928856 |
| solute carrier family 25, member 25 isoform c | 56699407 |
| solute carrier family 27 (fatty acid transporter), member 2 | 227499619 |
| solute carrier family 27 (fatty acid transporter), member 4 | 40807357 |
| solute carrier family 29 (nucleoside transporters), member 1 | 4826716 |
| solute carrier family 3, member 1 | 187423904 |
| sodium-dependent phosphate transport protein 2A isoform 1 | 156627569 |
| solute carrier family 35 (CMP-sialic acid transporter), member A1 | 5453621 |
| solute carrier family 35, member E1 | 164607128 |
| solute carrier family 35, member F3 | 27735127 |
| solute carrier family 36 (proton/amino acid symporter), member 4 | 40807351 |
| solute carrier family 37 (glycerol-3-phosphate transporter), member 2 | 38093649 |
| solute carrier family 37 member 1 | 49619231 |
| solute carrier family 38, member 2 | 21361602 |
| solute carrier family 38, member 4 | 18482385 |
| solute carrier family 39 (zinc transporter), member 13 | 40255101 |
| solute carrier family 4, anion exchanger, member 3 isoform 2 | 157671953 |
| solute carrier family 5 (inositol transporters), member 3 | 110835708 |
| solute carrier family 6, member 15 isoform 1 | 33354281 |
| solute carrier family 8 member 3 isoform C precursor | 33946311 |
| solute carrier family 9 (sodium/hydrogen exchanger), isoform 2 | 15529998 |
| solute carrier family 9 (sodium/hydrogen exchanger), isoform 5 | 4759144 |
| solute carrier family 9 (sodium/hydrogen exchanger), isoform 9 | 27734935 |
| solute carrier organic anion transporter family, member 4C1 | 38679890 |
| somatostatin receptor 2 | 4557859 |
| sortilin-related receptor containing LDLR class A repeats preproptein | 4507157 |
| sparc/osteonectin, cwcv and kazal-like domains proteoglycan | 4759164 |
| SPARC-like 1 | 190341024 |
| sperm associated antigen 9 isoform 1 | 27436920 |
| spermatogenesis associated 9 isoform a | 50659066 |
| SPPL3 protein | 33413418 |
| squalene monooxygenase | 62865635 |
| ST8 alpha-N-acetyl-neuraminide alpha-2,8-sialyltransferase 6 | 54234057 |
| stabilin 1 precursor | 61743978 |
| stabilin 2 precursor | 61743980 |
| stearoyl-CoA desaturase | 53759151 |
| sterile alpha motif domain containing 1 | 39930517 |
| sterolin 2 | 11967971 |
| steryl-sulfatase precursor | 53831991 |
| sulfatase 1 | 189571636 |
| sulfatase 2 isoform b precursor | 240255478 |
| suppressin | 38016945 |
| suppression of tumorigenicity 7 isoform b | 11761626 |
| surfeit 1 | 4507319 |
| sushi-repeat-containing protein, X-linked 2 | 7657619 |
| synaptotagmin-like 4 (granuphilin-a) | 193804860 |
| syntaxin 1A (brain) | 4759182 |
| syntaxin 4A (placental) | 20149560 |
| syntaxin 6 | 5032131 |
| TAO kinase 2 isoform 2 | 45505130 |
| taste receptor, type 1, member 2 | 112789566 |
| taste receptor, type 2, member 50 | 154937357 |
| taste receptor, type 2, member 7 | 12965176 |
| tectorin alpha precursor | 134268640 |
| TEK tyrosine kinase, endothelial precursor | 88758596 |
| tenascin N | 62988324 |
| testes development-related NYD-SP21 | 119226217 |
| testis enhanced gene transcript (BAX inhibitor 1) | 148746209 |
| testis expressed sequence 2 | 38679909 |
| thioredoxin domain containing 13 | 40254947 |
| thrombospondin 2 precursor | 40317628 |
| thrombospondin, type I, domain containing 6 | 226442878 |
| thymopoietin isoform beta | 73760405 |
| thyroglobulin | 55770862 |
| TLC domain containing 1 | 19923999 |
| toll-like receptor 1 | 41350337 |
| toll-like receptor 3 | 4507531 |
| toll-like receptor 4 precursor | 19924149 |
| tolloid-like 1 | 22547221 |
| TPA regulated locus | 32189371 |
| transferrin | 4557871 |
| transferrin receptor | 189458817 |
| transforming growth factor, beta receptor II isoform B precursor | 67782324 |
| transient receptor potential 4 | 7706747 |
| transient receptor potential cation channel, subfamily M, member 6 | 18921093 |
| transient receptor potential cation channel, subfamily M, member 7 | 148612863 |
| transient receptor potential cation channel, subfamily M, member 4 | 21314671 |
| transient receptor potential cation channel, subfamily V, member 5 | 17505200 |
| translocation protein 1 | 4507525 |
| transmembrane 4 superfamily member 6 | 4507541 |
| transmembrane 6 superfamily member 1 | 222136596 |
| transmembrane 7 superfamily member 3 | 7706575 |
| transmembrane anchor protein 1 isoform 1 | 56549131 |
| transmembrane and immunoglobulin domain containing | 45592949 |
| transmembrane channel-like 4 | 21389589 |
| transmembrane cochlear-expressed protein 2 | 94536852 |
| transmembrane emp24 domain containing 8 | 47106067 |
| transmembrane protease, serine 11B | 187761337 |
| transmembrane protease, serine 9 | 33667063 |
| transmembrane protein 10 isoform c | 91932793 |
| transmembrane protein 132B | 89111953 |
| transmembrane protein 132E | 46560555 |
| transmembrane protein 146 | 91598777 |
| transmembrane protein 15 | 7662482 |
| anoctamin-1 | 194306539 |
| anoctamin-3 | 156766084 |
| transmembrane protein 16G isoform NGEP long | 145207958 |
| transmembrane protein 57 | 31542661 |
| transmembrane protein 63B | 55769589 |
| transmembrane protein 63C | 190341095 |
| transmembrane protein 8 (five membrane-spanning domains) | 157676334 |
| transporter 1, ATP-binding cassette, sub-family B | 9665248 |
| triadin | 104526627 |
| trem-like transcript 2 protein precursor | 170932501 |
| tryptophan rich basic protein | 21536428 |
| tumor endothelial marker 8 isoform 1 precursor | 14149904 |
| tumor necrosis factor alpha | 25952111 |
| tumor necrosis factor receptor superfamily, member 10a | 259906438 |
| tumor necrosis factor receptor superfamily, member 8 isoform 11 | 68348711 |
| type 1 tumor necrosis factor receptor shedding aminopeptidase | 94818891 |
| type I hair keratin 1 | 14917115 |
| type IV alpha 6 collagen isoform A precursor | 148536823 |
| tyrosinase (oculocutaneous albinism IA) | 4507753 |
| tyrosylprotein sulfotransferase 1 | 4507665 |
| tyrosylprotein sulfotransferase 2 | 56699465 |
| ubiquitin protein ligase E3 component n-recognin 1 | 28372497 |
| ubiquitin specific protease 9, Y-linked | 74319833 |
| ubiquitin-activating enzyme E1 | 23510338 |
| UDP glycosyltransferase 2 family, polypeptide B17 | 4507821 |
| UDP glycosyltransferase 2 family, polypeptide B28 | 16596680 |
| UDP glycosyltransferase 3 family, polypeptide A2 | 270132412 |
| UDP-Gal:betaGlcNAc beta 1,4- galactosyltransferase 2 | 53759113 |
| UDP-GlcNAc:betaGal beta-1,3-N-acetylglucosaminyltransferase 2 | 9845238 |
| UDP-GlcNAc:betaGal beta-1,3-N-acetylglucosaminyltransferase 7 | 21687139 |
| UDP-GlcNAc:betaGal beta-1,3-N-acetylglucosaminyltransferase 8 | 38348376 |
| UDP-N-acetyl-alpha-D-galactosamine:polypeptide N-acetylgalactosaminyltransferase 12 (GalNAc-T12) | 112807221 |
| UDP-N-acetyl-alpha-D-galactosamine:polypeptide N-acetylgalactosaminyltransferase 5 (GalNAc-T5) | 32698686 |
| UDP-N-acetyl-alpha-D-galactosamine:polypeptide N-acetylgalactosaminyltransferase-like 1 | 270265820 |
| UDP-N-acetyl-alpha-D-galactosamine:polypeptide N-acetylgalactosaminyltransferase 5 (GalNAc-T5) | 32698686 |
| UDP-N-acetyl-alpha-D-galactosamine:polypeptide N-acetylgalactosaminyltransferase-like 5 | 281485547 |
| UNC13 (C. elegans)-like | 110611226 |
| unc-5 homolog B | 32261318 |
| uronyl-2-sulfotransferase | 5032219 |
| usherin isoform B | 219842266 |
| uveal autoantigen with coiled-coil domains and ankyrin repeats | 59850762 |
| VAMP-associated protein B/C | 4759302 |
| vasoactive intestinal peptide isoform 1 preproprotein | 4507897 |
| vasoactive intestinal peptide receptor 2 | 21361557 |
| vasohibin 1 | 7662454 |
| v-erb-a erythroblastic leukemia viral oncogene homolog 4 | 4885215 |
| very large G-protein coupled receptor 1 | 113722120 |
| vitamin D-binding protein precursor | 32483410 |
| vitelliform macular dystrophy 2-like 1 | 119703742 |
| v-myb myeloblastosis viral oncogene homolog | 46361980 |
| voltage-dependent calcium channel alpha 1G subunit isoform 7 | 38505274 |
| voltage-gated calcium channel alpha(2)delta-4 subunit isoform | 132566536 |
| voltage-gated potassium channel, subfamily H, member 2 | 26051273 |
| voltage-gated sodium channel beta-3 subunit precursor | 9055238 |
| von Willebrand factor preproprotein | 89191868 |
| VPS10 domain receptor protein SORCS 2 | 170014689 |
| wingless-type MMTV integration site family, member 3A | 14916475 |
| wingless-type MMTV integration site family, member 8B precurso | 110735437 |
| wingless-type MMTV integration site family, member 9B precurso | 17017976 |
| X-linked ectodysplasin receptor | 11140823 |
| X-linked juvenile retinoschisis protein | 10835083 |
| X-linked phosphate regulating endopeptidase homolog | 90403592 |
| X-prolyl aminopeptidase 2, membrane-bound | 93141226 |
| xylosyltransferase I | 28269693 |
| Yip1 interacting factor homolog | 170932464 |
| zona pellucida binding protein | 229577313 |
| zona pellucida glycoprotein 1 | 66348148 |
| zonadhesin isoform 6 | 27881494 |
